# Lymphocyte count is a universal predictor of health outcomes in COVID-19 patients before mass vaccination: A meta-analytical study

**DOI:** 10.7189/jogh.12.05041

**Published:** 2022-09-17

**Authors:** Kuan-Lang Lai, Fu-Chang Hu, Fang-Yu Wen, Ju-Ju Chen

**Affiliations:** 1 Graduate Institute of Public Health, School of Public Health, National Defense Medical Center, Taipei, Taiwan; 2 CJ Consulting-Expert Co., Ltd., Taipei, Taiwan; 3 Graduate Institute of Clinical Medicine and School of Nursing, College of Medicine, National Taiwan University, Taipei, Taiwan; 4 Statistical Consulting Clinic, International-Harvard (I-H) Statistical Consulting Company, Taipei, Taiwan

## Abstract

**Background:**

Several laboratory data have been identified as predictors of disease severity or mortality in COVID-19 patients. However, the relative strength of laboratory data for the prediction of health outcomes in COVID-19 patients has not been fully explored. This meta-analytical study aimed to evaluate the prediction capabilities of laboratory data on the prognosis of COVID-19 patients during 2020 while mass vaccination has not started yet.

**Methods:**

Two electronic databases, MEDLINE and EMBASE, from inception to October 10, 2020 were searched. Observational studies of laboratory-confirmed COVID-19 patients with well-defined severity or survival status, and with the desired laboratory data at initial hospital administrations, were selected. Meta-regression analysis with the generalized estimating equations (GEE) method for clustered data was performed sequentially. Primary outcome measures were to compare the level of laboratory data and their impact on different health outcomes (severe vs non-severe, critically severe vs non-critically severe, and dead vs alive).

**Results:**

Meta-data of 13 clinical laboratory items at initial hospital presentations were extracted from 76 selected studies with a total of 26 627 COVID-19 patients in 16 countries. After adjusting for the effect of age, 1.03 <lymphocyte count mean or median ( × 10^9^/L) ≤2.06 (estimated odds ratio (OR) = 0.0216; 95% confidence interval (CI) = 0.0041-0.1131; *P* < 0.0001), higher lymphocyte count mean or median ( × 10^9^/L) (OR <0.0001; 95% CI: <0.0001-0.0386; *P* = 0.0284), and lymphocyte count mean or median ( × 10^9^/L) >0.87 (OR = 0.0576; 95% CI = 0.0043-0.4726; *P* = 0.0079) had a much lower risk of severity, critical severity, and mortality from COVID-19, respectively.

**Conclusions:**

Lymphocyte count was the most powerful predictor among the 13 common laboratory variables explored from COVID-19 patients to differentiate disease severity and to predict mortality. Lymphocyte count should be monitored for the prognoses of COVID-19 patients in clinical settings in particular for patients not fully vaccinated.

Although numerous treatment options and vaccines are authorized for COVID-19 [1,2], the situation of a global pandemic is continuing. Over 3.5 million new cases a week are reported at the time of May 2022 [3]. COVID-19, the disease caused by SARS-CoV-2 [4] infection has spread since December 2019 from Wuhan, China, and has accumulated more than 519 million cases and more than 6 million deaths worldwide [5]. During the pandemic period, the medical care system started to overwhelm communities in groups, regardless of economically developed or underdeveloped regions [6-10]. How to use simple tools to differentiate and classify patients is crucial.

Several laboratory data have been identified as predictors of disease severity or mortality in COVID-19 patients [11-13]. However, since many studies were conducted in same region during a short period of time, potential bias of subject duplication cannot be ruled out for the following meta-analysis [14-19]. Furthermore, the relative strength of laboratory data of a broad spectrum for their prediction ability has not been explored head-to-head. Thus, this study aimed to investigate whether laboratory data at hospital presentation play a role in distinguishing severity or predicting mortality for patients with COVID-19 before mass vaccination and to explore the relative importance of these predictors.

## METHODS

### Literature search

We used the search terms “COVID-19”, “2019-nCoV”, and “coronavirus” in the search field “title / abstract” in two electronic databases: MEDLINE and EMBASE. The searches were completed on October 10, 2020.

### Inclusion and exclusion criteria

The eligibility criteria for the inclusion of literature in the meta-analysis were the following: (1) the literature is the original research; (2) the literature was a study with laboratory-confirmed COVID-19 patients; (3) the literature was published in English with the full text available; (4) the source of subjects and the recruitment situation were clearly stated. The literature was excluded from the meta-analysis when (1) the severity or survival status of the disease was not well defined; (2) a paediatric study or a particular subject group, for example, a specific disease apart from COVID-19; (3) the 13 laboratory data desired at initial hospital administrations for COVID-19 were not available, which included white blood cells (WBC), neutrophil, lymphocyte, neutrophil-to-cell ratio (NLR), platelet, alanine aminotransferase (ALT), aspartate aminotransferase (AST), creatinine, D-dimer, C-reactive protein (CRP), procalcitonin (PCT), lactate dehydrogenase (LDH), and hypersensitive troponin I (hs-cTnI); (4) subject number below thirty. The eligible articles after the above evaluation would go through (5) the assessment of concerns about duplicate subjects for studies with COVID-19 subjects recruited from the same hospitals during an overlapping period. Under condition (5), only one study provided the most information by calculating (the number of study subjects) × (number of laboratory data items) was selected.

**Figure 1:**
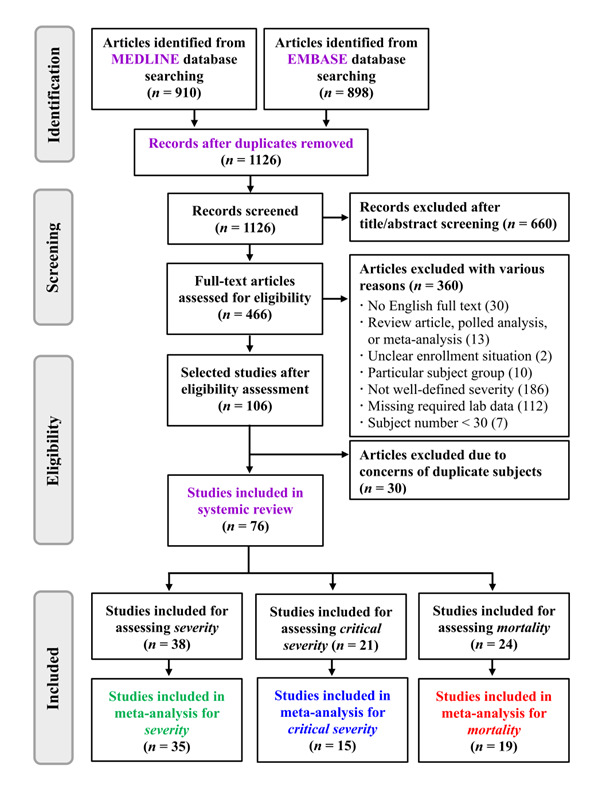
Flow diagram of the literature search and study selection.

As shown in **Figure 1**, 1126 records were identified from the MEDLINE and EMBASE databases after the duplicates were removed and 660 articles were excluded after the titles and abstracts were reviewed. The 466 remaining articles were carefully and thoroughly evaluated. Finally, 360 articles were excluded for various reasons and another 30 articles were excluded solely due to concerns of duplication of the subject.

### Data extraction

JJC and KLL extracted the following data from the qualified studies by hand searching: first author, year/month of publication, location (city, country), hospital name, definition of disease severity, recruitment period, subject number, number of COVID-19 patients in each health status, age, male to female ratio, vital signs, clinical characteristics (12 symptoms), comorbidities (any; 8 main diseases), and 13 desired laboratory items (see Tables S1-S4 in the **Online Supplementary Document**). Laboratory data at initial hospital presentations were classified as blood routine, blood biochemistry, coagulation functions, inflammatory markers, and markers of myocardial injury (see Tables S2-S4 in the **Online Supplementary Document**). The authors followed the Meta-analyses Of Observational Studies in Epidemiology (MOOSE) in study selection. The quality of included studies was assessed using a methodological index for non-randomized studies (MINORS) (data not shown). Several authors of selected papers were contacted by email to clarify data issues.

Primary outcome measures were to compare the level of laboratory data and their impact on different health outcomes (severe vs non-severe, critically severe vs non-critically severe, and dead vs alive) after adjusting the effects of other covariates.

### Statistical analysis

Statistical analysis was performed using the R 4.1.2 software (R Foundation for Statistical Computing, Vienna, Austria). The two-sided *P*-value ≤0.05 was considered statistically significant. We chose the outcome groups in the collected studies as the analysis unit, instead of the collected studies themselves, in this meta-analytical study. The distributional properties of continuous variables were expressed by mean ± standard deviation (SD), median, interquartile range (IQR), and categorical variables were presented by frequency and percentage (%). In the univariate analysis, the unadjusted effect of each potential risk factor, prognostic factor, or predictor of the three binary outcome variables (ie, severe vs non-severe, critically severe vs non-critically severe, and dead vs alive) was examined, respectively, using the Wilcoxon rank-sum test, χ^2^ test, and Fisher exact test as appropriate for the type of data. In addition, the 39 forest plots of mean/median differences in the 13 laboratory variables considered were drawn between the COVID-19 patients with severe vs non-severe statuses, critically severe vs non-critically severe statuses, and dead vs alive outcomes, respectively. The weighted mean of each status/outcome group was calculated using the same weights as those used in the calculation of the pooled mean/median difference.

Next, multivariate analysis was performed by fitting logistic regression models to estimate the adjusted effects of potential risk factors, prognostic factors, or predictors on the three binary outcome variables (ie, severe vs non-severe, critically severe vs non-critically severe, and dead vs alive), respectively, with the generalized estimating equations (GEE) method. The GEE method was used to account for the correlation between the two outcome groups within a collected study [20]. Computationally, we used the geeglm function (with the specified “exchangeable” correlation structure and the default robust estimator of standard error) of the geepack package [21,22] to fit GEE logistic regression models for the three sets of correlated binary responses (ie, severe vs non-severe, critically severe vs non-critically severe, and dead vs alive) in R, respectively.

### Model-fitting techniques

To ensure good quality of analysis, the model-fitting techniques for (1) variable selection, (2) goodness-of-fit (GOF) assessment, and (3) regression diagnostics and remedies were used in our GEE logistic regression analyses. All relevant univariate significant and non-significant covariates (listed in Table S2 in the **Online Supplementary Document**) were placed on the variable list to be selected. However, each of the collected studies selectively reported the potential risk factor, prognostic factor, or predictor of the three binary outcome variables. If we wanted to simultaneously assess the effects of all relevant covariates (listed in Table S2 in the **Online Supplementary Document**), then the number of studies without missing values would be very few. Thus, our meta-regression analysis was performed by fitting a series of simple GEE logistic regression models and then dropping the worst one at a time to maximally use all the available information. Then, a final multiple GEE logistic regression model was obtained for each of the three outcome variables. Any discrepancy between the results of univariate analysis and multivariate analysis was probably due to the variation in the number of studies without missing values or the confounding effects of uncontrolled covariates in univariate analysis.

The GOF measures, including the estimated area under the curve (AUC) of the Receiver Operating Characteristic (ROC) (also called the *c *statistic) and the adjusted generalized *R*^2^, and the Hosmer-Lemeshow GOF test were examined to assess the GOF of the fitted GEE logistic regression model. The value of the *c* statistic (0 ≤ *c* ≤ 1) ≥ 0.7 suggests an acceptable level of discrimination power. Larger *P* values of the Hosmer-Lemeshow GOF test imply better fits of the logistic regression model.

Simple and multiple generalized additive models (GAMs) were fitted to draw the GAM plots for detecting nonlinear effects of continuous covariates and then for identifying the appropriate cut-off point(s) to discretize continuous covariates, if necessary, during the above variable selection procedure. Computationally, we used the vgam function of the VGAM package with the default values of the smoothing parameters (eg, s(age, df = 4, spar = 0) for the cubic smoothing splines) to fit the GAMs for our binary responses, and then used the plotvgam function of the same package to draw the GAM plots for visualizing the linear or nonlinear effects of continuous covariates in R [21,23,24]. If a separation or high discrimination problem occurred in logistic regression analysis, we fitted the Firth’s bias-reduced logistic regression model using the logistf function of the logistf package in R [25]. Finally, the statistical tools of regression diagnostics for residual analysis, detection of influential cases, and check of multicollinearity were applied to discover any model or data problems. The values of the variance inflating factor (VIF)  ≥10 in continuous covariates or VIF ≥2.5 in categorical covariates indicate the occurrence of the multicollinearity problem among some of the covariates in the fitted logistic regression model.

## RESULTS

### Profile of the collected studies

As shown in **Figure 1** and Table S1 in the **Online Supplementary Document**, 76 articles with 26 627 COVID-19 patients were included in this meta-analytical study. Specifically, the number of studies (patients) included for the analysis of severity, critical severity, and mortality was 38 studies (9764 patients), 21 studies (4792 patients), and 24 studies (14 825 patients). Finally, a total of 35 studies, 15 studies, and 19 studies were presented in three separate meta-regression analyses.

Summary statistics of patient demographics, clinical characteristics, comorbidities, and laboratory data of COVID-19 patients at initial hospital presentations for the evaluation of severity, critical severity, and mortality are shown in Tables S2-S4 in the **Online Supplementary Document**, respectively. All 76 selected studies were published in 2020. The number of patients in each study ranged from 38 to 4035 subjects, who were recruited between December 1, 2019 and June 27, 2020, in 16 countries. The definition of severity was based on the WHO interim guidance [26] or a national guidance modified from the WHO principles [27] in most of the 55 studies (49, 89.1%), followed by the American Thoracic Society Guideline [28] (5, 9.1%) and the International Guideline for Community-Acquired Pneumonia (1, 1.8%). Since the content of the above guidelines was similar, they were all included in the meta-analysis. In general, the severity of the disease is classified into four types: mild, moderate, severe, and critically severe. In particular, severe and critically severe were defined below:

- Severe: meet any of the following criteria. (1) Shortness of breath (i.e., respiratory rate (RR) >30 times per minute); (2) in room air, oxygen saturation by pulse oximetry (SpO^2^) <93%; (3) partial pressure of oxygen (PaO^2^)/fraction of inspired oxygen (FiO^2^) ≤300 mmHg; or (4) computed tomography (CT) chest imaging shows that lung damage develops significantly within 24 to 48 hours.
- Critically severe: meet any of the following criteria. (1) Respiratory failure that requires mechanical ventilation; (2) signs of septic shock; or (3) multiple organ failure that requires admission to the ICU.

For the comparisons in this study, subjects with mild and moderate conditions were grouped into the non-severe group, and those with mild, moderate, and severe conditions were grouped into the non-critically severe group. Thus, this study examined three binary outcome variables with two levels each: severe vs non-severe, critically severe vs non-critically severe, dead vs alive.

### Forest plots

The 3 forest plots of mean/median differences in lymphocyte counts between the COVID-19 patients with severe vs non-severe statuses, critically severe vs non-critically severe statuses, and dead vs alive outcomes are shown in **Figures 2-4**. We found that the weighted group means of lymphocyte counts for being severe, critically severe, and dead decreased from 0.815, 0.746, to 0.703 as expected. The 36 forest plots of mean/median differences for the other 12 laboratory variables between the groups of severe vs non-severe, critically severe vs non-critically severe, and dead vs alive are shown in Figures S1-S24 and Figures S25-S36 in the **Online Supplementary Document**. Many investigators did not examine some of the laboratory variables such as NLR and hs-cTnI.

**Figure 2:**
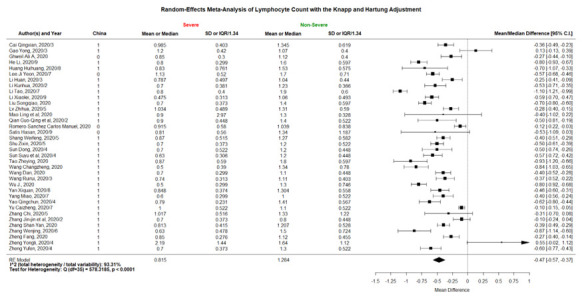
Forest plot of mean/median differences in lymphocyte count ( × 10^9^/L) between COVID-19 patients with severe or non-severe status (the numbers of 0.815 and 1.284 at the bottom were the weighted means using the same weights as “mean/median difference”).

**Figure 3:**
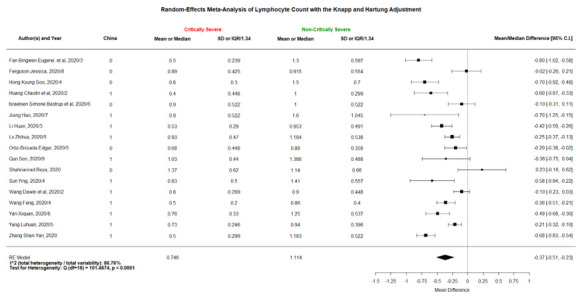
Forest plot of mean/median differences in lymphocyte count ( × 10^9^/L) between COVID-19 patients with critically severe or non-critically severe status (the numbers of 0.746 and 1.114 at the bottom were the weighted means using the same weights as “mean/median difference”).

**Figure 4:**
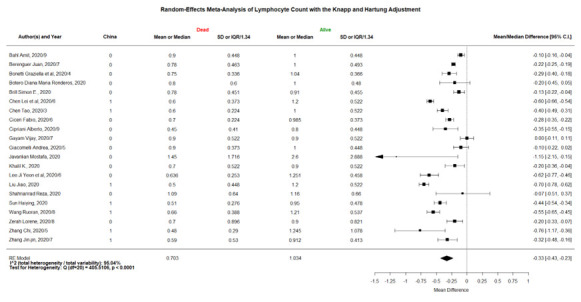
Forest plot of mean/median differences in lymphocyte count ( × 10^9^/L) between COVID-19 patients with dead vs alive outcome (the numbers of 0.703 and 1.034 at the bottom were the weighted means using the same weights as “mean/median difference”).

### Predictors of severity (severe vs non-severe)

The results of a series of simple logistic regression analyses (with the GEE method for clustered data) to identify predictors of severity (severe vs non-severe) are listed in **Table 1**. As less effective laboratory items were discarded according to the order of the AUC of the ROC, more arms (*m*) were recruited in the meta-regression analysis from *m* = 14 to *m* = 70 in the different runs. In the final run (seventh, *m* = 70), only lymphocyte count ( × 10^9^/L) (AUC = 0.938) or lymphocyte count ( × 10^9^/L) ≤ 1.03 or > 2.06 (AUC = 0.929) and age (years) (AUC = 0.855) or age (years) >55.02 (AUC = 0.800) existed in the final univariate analyses. Then, the result of the final multiple logistic regression analysis for the prediction of severity (severe vs non-severe) was listed in **Table 2**. We found that age mean or median (years) >55.02 had a high risk of severity (estimated odds ratio (OR) = 5.7921; 95% confidence interval (CI) = 1.6638-20.1634; *P* = 0.0058) while 1.03 < lymphocyte count mean or median ( × 10^9^/L) ≤2.06 showed a strong protection against severity (OR = 0.0216; 95% CI = 0.0041-0.1131; *P* < 0.0001) with the AUC = 0.968.

**Table 1:** Univariate analyses of the predictors of severity (severe vs non-severe) by fitting a series of simple logistic regression models with the generalized estimating equations (GEE) method (assuming an “exchangeable” working correlation structure)

Order*	Covariate	AUC of ROC	95% CI of AUC of ROC	Residual Deviance	Nagelkerke’s *R*^2^
	**Run 1: *m* = 10**				
1	CRP mean or median (mg/L)	1.000	1-1	0.0000	1.0000
2	D-dimer mean or median (mg/L or μg/mL)	1.000	1-1	0.0000	1.0000
3	Lymphocyte count mean or median ( × 10^9^/L)	1.000	1-1	0.0000	1.0000
4	Neutrophil count mean or median ≤2.50 or >3.74 ( × 10^9^/L)	1.000	1-1	0.0000	1.0000
5	Platelet count mean or median ≤194.99 or >232.94 ( × 10^9^/L)	1.000	1-1	0.0000	1.0000
6	Age mean or median (years)	0.920	0.736-1	6.8134	0.6745
7	LDH mean or median >214.99 (U/L)	0.900	0.704-1	5.4067	0.7609
8	PCT mean or median >5.48 ( × 100 ng/mL)	0.900	0.704-1	5.4067	0.7609
9	White blood cell count mean or median ≤4.21 or >5.72 ( × 10^9^/L)	0.900	0.704-1	5.4067	0.7609
10	Age mean or median >56.50 (years)	0.800	0.560-1	8.3758	0.5631
11	Percentage of male gender	0.560	0.153-0.967	13.7379	0.0166
	**Run 2: *m* = 14**				
1	CRP mean or median (mg/L)	1.000	1-1	0.0000	1.0000
2	D-dimer mean or median (mg/L or μg/mL)	1.000	1-1	0.0000	1.0000
3	Lymphocyte count mean or median ( × 10^9^/L)	1.000	1-1	0.0000	1.0000
4	Neutrophil count mean or median ≤2.39 or >3.70( × 10^9^/L)	1.000	1-1	0.0000	1.0000
5	Age mean or median (years)	0.939	0.821-1	8.6161	0.7165
6	Age mean or median >56.05 (years)	0.857	0.676-1	- ^2^	0.5670
7	LDH mean or median >224.20 (U/L)	0.857	0.659-1	11.4833	0.5763
8	Platelet count mean or median ≤197.80 or >236.14 ( × 10^9^/L)	0.786	0.557-1	14.4041	0.4007
	**Run 3: *m* = 16**				
1	CRP mean or median (mg/L)	1.000	1-1	0.0000	1.0000
2	Lymphocyte count mean or median ( × 10^9^/L)	1.000	1-1	0.0000	1.0000
3	D-dimer mean or median (mg/L or μg/mL)	0.984	0.941-1	4.3995	0.8945
4	Age mean or median (years)	0.953	0.862-1	8.8730	0.7529
5	LDH mean or median (U/L)	0.953	0.851-1	10.2592	0.7004
6	Age mean or median >55.64 (years)	0.875	0.715-1	10.0080	0.7103
7	Neutrophil count mean or median >3.71 ( × 10^9^/L)	0.875	0.702-1	12.0566	0.6252
	**Run 4: *m* = 18**				
1	CRP mean or median (mg/L)	1.000	1-1	0.0000	1.0000
2	Lymphocyte count mean or median ( × 10^9^/L)	1.000	1-1	0.0000	1.0000
3	D-dimer mean or median (mg/L or μg/mL)	0.988	0.953-1	4.4012	0.9077
4	LDH mean or median (U/L)	0.963	0.882-1	10.5782	0.7334
5	Age mean or median (years)	0.938	0.834-1	10.4480	0.7377
6	Age mean or median >55.26 (years)	0.889	0.745-1	10.4311	0.7383
	**Run 5: *m* = 28**				
1	Lymphocyte count mean or median ( × 10^9^/L)	0.929	0.820-1	19.4244	0.6663
2	Age mean or median (years)	0.888	0.766-1	25.0481	0.5179
3	CRP mean or median >27.15 (mg/L)	0.821	0.675-0.968	26.1745	0.4844
4	Age mean or median >57.33 (years)	0.750	0.584-0.916	31.4428	0.3087
5	D-dimer mean or median >0.58 (mg/L or μg/mL)	0.750	0.584-0.916	31.4428	0.3087
	**Run 6: *m* = 56**				
1	Lymphocyte count mean or median ( × 10^9^/L)	0.952	0.897-1	30.6650	0.7570
2	CRP mean or median (mg/L)	0.921	0.842-1	48.7910	0.5367
3	Age mean or median (years)	0.874	0.778-0.970	51.0981	0.5032
4	CRP mean or median >26.52 (mg/L)	0.839	0.741-0.937	49.3143	0.5292
5	Age mean or median >56.06† (years)	0.804	0.698-0.909	55.4443	0.4362
	**Run 7: *m* = 70**				
1	Lymphocyte count mean or median ( × 10^9^/L)	0.938	0.872-1	55.4496	0.5973
2	Lymphocyte count mean or median ≤1.03 or >2.06 ( × 10^9^/L)	0.929	0.867-0.990	35.8650	0.7769
3	Age mean or median (years)	0.855	0.762-0.948	66.2054	0.4750
4	Age mean or median >55.02 (years)	0.800	0.705-0.895	69.9269	0.4282

AUC of ROC – area under the curve of the receiver operating characteristic, CI – confidence interval, CRP – C-reactive protein, PCT – procalcitonin, LDH – lactate dehydrogenase

*Simple logistic regression models were listed in order of the values of AUC of ROC.

†The 5 groups with “age mean or median ≤56.06 years” were all non-severe so that the separation or high discrimination problem occurred in fitting the simple logistic regression model with the generalized estimating equations (GEE) method (assuming an “exchangeable” working correlation structure). Then, the logistf() function of the logistf package was used to fit the Firth’s bias-reduced logistic regression model with the profile penalized log-likelihood method in R. Since the “residual deviance” of the Firth’s bias-reduced logistic regression model was not compatible with the others, it was not computed and listed.

**Table 2:** Multivariate analysis of the predictors of severity (severe vs non-severe) by fitting a multiple logistic regression model with the generalized estimating equations (GEE) method (assuming an ‘exchangeable’ working correlation structure)*

Covariate	Estimated regression coefficient	Standard error	χ^2^ value	*P*-value	Estimated OR	95% CI of OR
Intercept	1.1346	0.6238	3.3085	0.0689	3.1101	0.9157-10.5628
1.03 × 10^9^/L<Lymphocyte count mean or median ≤2.06 × 10^9^/L	-3.8343	0.8442	20.6299	<0.0001	0.0216	0.0041-0.1131
Age mean or median†>55.02 years	1.7565	0.6364	7.6174	0.0058	5.7921	1.6638-20.1634

OR – odds ratio, CI – confidence interval

*Goodness-of-fit assessment – number of studies n = 35, number of arms *m* = 70 (cluster size = 2), the estimated area under the receiver operating characteristic (ROC) curve = 0.968 > 0.7 with 95% CI = 0.928-1 (DeLong).

†Although “age mean or median” beat “age mean or median >55.02 years” in Run 7 of **Table 1** (AUC of ROC = 0.855 vs 0.800), we chose “age mean or median >55.02 years” in this multiple logistic regression model because the GAM plot of “age mean or median” indicated that its effect on logit (probability of being severe) flattened for age’s mean or median ≤55.02 years.

### Predictors of critical severity (critically severe vs non-critically severe)

Results of univariate analyses of the predictors for critical severity (critically severe vs non-critically severe) are shown **Table 3**. In the last run (seventh, *m* = 30) only lymphocyte count (AUC = 0.933) and age (years) (AUC = 0.829) or age (years) > 59.82 (AUC = 0.767) existed in the final univariate analyses. The result of multivariate analysis for the predictors of critical severity (critically severe vs non-critically severe) is listed in **Table 4**. We found that the higher lymphocyte count mean or median had an extremely lower risk of critical severity (OR <0.0001; 95% CI = <0.0001-0.0386; *P* = 0.0284) while the age mean or median (years) >59.82 had a higher risk of critical severity (OR = 307.6130; 95% CI = 10.4237-9077.9402; *P* = 0.0009).

**Table 3:** Univariate analyses of the predictors of critical severity (critically severe vs non-critically severe) by fitting a series of simple logistic regression models with the generalized estimating equations (GEE) method (assuming an “exchangeable” working correlation structure)

Order*	Covariate	AUC of ROC	95% CI of AUC of ROC	Residual Deviance	Nagelkerke’s *R*^2^
	**Run 1: *m* = 6**				
1	Age mean or median (years)	1.000	1-1	0.0000	1.0000
2	LDH mean or median (U/L)	1.000	1-1	0.0000	1.0000
3	Lymphocyte count mean or median ( × 10^9^/L)	1.000	1-1	0.0000	1.0000
4	Neutrophil count mean or median ( × 10^9^/L)	1.000	1-1	0.0000	1.0000
5	CRP mean or median (mg/L)	0.889	0.581-1	6.1823	0.3993
6	D-dimer mean or median (mg/L or μg/mL)	0.889	0.581-1	4.2344	0.6582
7	White blood cell count mean or median ( × 10^9^/L)	0.889	0.581-1	3.7726	0.7082
8	Platelet count mean or median ( × 10^9^/L)	0.667	0.013-1	8.2068	0.0244
	**Run 2: *m* = 8**				
1	Lymphocyte count mean or median ( × 10^9^/L)	1.000	1-1	0.0000	1.0000
2	Neutrophil count mean or median ( × 10^9^/L)	1.000	1-1	0.0000	1.0000
3	Age mean or median (years)	0.938	0.764-1	4.8792	0.7199
4	LDH mean or median (U/L)	0.938	0.764-1	3.7758	0.7990
5	White blood cell count mean or median ( × 10^9^/L)	0.875	0.592-1	4.8638	0.7211
6	CRP mean or median (mg/L)	0.812	0.481-1	8.2593	0.3974
7	D-dimer mean or median (mg/L or μg/mL)	0.750	0.350-1	9.8188	0.1959
	**Run 3: *m* = 12**				
1	Lymphocyte count mean or median ( × 10^9^/L)	1.000	1-1	0.0000	1.0000
2	Neutrophil count mean or median ( × 10^9^/L)	1.000	1-1	0.0000	1.0000
3	Age mean or median (years)	0.944	0.816-1	5.8139	0.7922
4	White blood cell count mean or median ( × 10^9^/L)	0.917	0.738-1	6.3748	0.7663
5	CRP mean or median (mg/L)	0.833	0.595-1	11.3907	0.4721
6	LDH mean or median (U/L)	0.806	0.515-1	12.7967	0.3650
	**Run 4: *m* = 16**				
1	Age mean or median (years)	0.938	0.807-1	10.4603	0.6924
2	Lymphocyte count mean or median ( × 10^9^/L)	0.938	0.824-1	9.1502	0.7428
3	Neutrophil count mean or median ( × 10^9^/L)	0.938	0.807-1	10.9031	0.6744
4	White blood cell count mean or median ( × 10^9^/L)	0.844	0.624-1	13.9161	0.5379
5	CRP mean or median (mg/L)	0.781	0.543-1	17.3436	0.3479
	**Run 5: *m* = 24**				
1	Lymphocyte count mean or median ( × 10^9^/L)	0.927	0.828-1	15.3659	0.7010
2	Neutrophil count mean or median ( × 10^9^/L)	0.903	0.784-1	17.4015	0.6450
3	Age mean or median (years)	0.878	0.735-1	20.8519	0.5386
4	White blood cell count mean or median ( × 10^9^/L)	0.868	0.715-1	20.6280	0.5460
5	Age mean or median >60.73 (years)	0.833	0.682-0.985	21.0499	0.5320
	**Run 6: *m* = 24**				
1	Lymphocyte count mean or median ( × 10^9^/L)	0.927	0.828-1	15.3659	0.7010
2	Neutrophil count mean or median ( × 10^9^/L)	0.903	0.784-1	17.4015	0.6450
3	Age mean or median (years)	0.878	0.735-1	20.8519	0.5386
4	Age mean or median >60.72 (years)	0.833	0.682-0.985	21.0499	0.5320
	**Run 7: *m* = 30**				
1	Lymphocyte count mean or median ( × 10^9^/L)	0.933	0.850-1	18.2773	0.7203
2	Age mean or median (years)	0.829	0.668-0.990	31.0932	0.3936
3	Age mean or median >59.82 (years)	0.767	0.610-0.923	32.5430	0.3471

AUC of ROC – area under the curve of the receiver operating characteristic, CI – confidence interval, CRP – C-reactive protein, PCT – procalcitonin, LDH – lactate dehydrogenase

*Simple logistic regression models were listed in order of AUC of ROC.

**Table 4:** Multivariate analysis of the predictors of critical severity (critically severe vs non-critically severe) by fitting a multiple logistic regression model with the generalized estimating equations (GEE) method (assuming an “exchangeable” working correlation structure)*

Covariate	Estimated regression coefficient	Standard error	χ^2^ value	*P*-value	Estimated OR	95% CI of OR
Intercept	24.5729	11.5788	4.5038	0.0338	>1000	6.5426->9999
Lymphocyte count mean or median ( × 10^9^/L)	-30.7807	14.0442	4.8035	0.0284	<0.0001	<0.0001-0.0386
Age mean or median†>59.82 years	5.7288	1.7269	11.0050	0.0009	307.6130	10.4237-9077.9402

OR – odds ratio, CI – confidence interval

*Goodness-of-fit assessment – number of studies n = 15, number of arms *m* = 30 (cluster size = 2), the estimated area under the receiver operating characteristic (ROC) curve = 0.991 > 0.7 with 95% CI = 0.970-1 (DeLong).

†Although “age mean or median” beat “age mean or median >59.82 years” in Run 7 of **Table 3** (AUC of ROC: 0.829 vs 0.767), we chose “age mean or median >59.82 years” in this multiple logistic regression model because the GAM plot of “age mean or median” indicated that its effect on logit (probability of being severe) flattened for age’s mean or median between 50 and 60 years and decreased for age’s mean or median >67.5 years.

### Predictors of mortality (dead vs alive)

Results of univariate analyses of the predictors for mortality (dead vs alive) are shown in **Table 5**. In the last run (seventh, *m* = 38) only lymphocyte count ( × 10^9^/L) (AUC = 0.935) or lymphocyte count ( × 10^9^/L) ≤0.87 (AUC = 0.895) and age (years) (AUC = 0.913) or age (years) > 67.28 (AUC = 0.895) existed in the final univariate analyses. The result of multivariate analysis of the predictors of mortality is listed in **Table 6**. Older age mean or median (years) >67.28 had a higher risk of mortality (OR = 17.3756; 95% CI = 2.1157-232.7834; *P* = 0.0079), while lymphocyte count mean or median ( × 10^9^/L) >0.87 had a lower risk of mortality (OR = 0.0576; 95% CI = 0.0043-0.4726; *P* = 0.0079).

**Table 5:** Univariate analyses of the predictors of mortality (dead vs alive) by fitting a series of simple logistic regression models with the generalized estimating equations (GEE) method (assuming an “exchangeable” working correlation structure)

Order*	Covariate	AUC of ROC	95% CI of AUC of ROC	Residual Deviance	Nagelkerke’s *R*^2^
	**Run 1: *m* = 12**				
1	Age mean or median (years)	1.000	1-1	0.0000	1.0000
2	Lymphocyte count mean or median ( × 10^9^/L)	0.986	0.948-1	2.7726	0.9134
3	CRP mean or median (mg/L)	0.972	0.895-1	3.6475	0.8816
4	LDH mean or median (U/L)	0.944	0.816-1	7.1707	0.7274
5	Neutrophil count mean or median ( × 10^9^/L)	0.944	0.816-1	5.0828	0.8242
6	White blood cell count mean or median ( × 10^9^/L)	0.944	0.816-1	6.2940	0.7701
7	D-dimer mean or median (mg/L or μg/mL)	0.917	0.738-1	8.4516	0.6592
8	D-dimer mean or median >1.94 (mg/L or μg/mL)	0.917	0.753-1	5.7416	0.7955
9	LDH mean or median >443.26 (U/L)	0.917	0.753-1	5.7416	0.7955
10	Platelet count mean or median ( × 10^9^/L)	0.861	0.625-1	9.9462	0.5698
11	Platelet count mean or median ≤183.69 ( × 10^9^/L)	0.833	0.602-1	10.8135	0.5125
	**Run 2: *m* = 14**				
1	Lymphocyte count mean or median ( × 10^9^/L)	0.990	0.962-1	2.7726	0.9270
2	Age mean or median (years)	0.959	0.869-1	6.3268	0.8096
3	Neutrophil count mean or median ( × 10^9^/L)	0.959	0.864-1	5.1213	0.8528
4	White blood cell count mean or median ( × 10^9^/L)	0.959	0.864-1	6.3653	0.8081
5	LDH mean or median (U/L)	0.939	0.806-1	10.8264	0.6110
6	CRP mean or median (mg/L)	0.878	0.667-1	13.2424	0.4750
7	CRP mean or median >85.76 (mg/L)	0.857	0.659-1	11.4833	0.5763
8	LDH mean or median >503.93 (U/L)	0.857	0.659-1	11.4833	0.5763
	**Run 3: *m* = 16**				
1	Lymphocyte count mean or median ( × 10^9^/L)	0.992	0.971-1	2.7726	0.9369
2	LDH mean or median (U/L)	0.938	0.807-1	12.4176	0.6090
3	Age mean or median (years)	0.922	0.794-1	10.8942	0.6748
4	Neutrophil count mean or median ( × 10^9^/L)	0.906	0.756-1	10.2175	0.7021
5	White blood cell count mean or median ( × 10^9^/L)	0.906	0.756-1	11.8361	0.6349
6	LDH mean or median >501.70 (U/L)	0.875	0.702-1	12.0566	0.6252
7	White blood cell count mean or median >6.74 ( × 10^9^/L)	0.812	0.611-1	15.2763	0.4673
	**Run 4: *m* = 20**				
1	Lymphocyte count mean or median ( × 10^9^/L)	0.990	0.966-1	3.8191	0.9299
2	Age mean or median (years)	0.930	0.821-1	12.1678	0.7208
3	Neutrophil count mean or median ( × 10^9^/L)	0.920	0.797-1	12.6233	0.7067
4	LDH mean or median (U/L)	0.910	0.783-1	16.9125	0.5569
5	LDH mean or median >479.84 (U/L)	0.850	0.687-1	16.7100	0.5647
	**Run 5: *m* = 28**				
1	Lymphocyte count mean or median ( × 10^9^/L)	0.995	0.983-1	3.8191	0.9513
2	Age mean or median (years)	0.954	0.882-1	13.6223	0.7911
3	Neutrophil count mean or median ( × 10^9^/L)	0.913	0.811-1	19.5828	0.6625
	**Run 6: *m* = 38**				
1	Lymphocyte count mean or median ( × 10^9^/L)	0.935	0.836-1	31.0924	0.5778
2	Age mean or median (years)	0.913	0.807-1	30.6503	0.5866
3	Age mean or median >67.28 (years)	0.895	0.796-0.994	24.8313	0.6926
4	Lymphocyte count mean or median ≤0.87 ( × 10^9^/L)	0.895	0.796-0.994	24.8313	0.6926

AUC of ROC – area under the curve of the receiver operating characteristic, CI – confidence interval, CRP – C-reactive protein, PCT – procalcitonin, LDH – lactate dehydrogenase

*Simple logistic regression models were listed in order of AUC of ROC.

**Table 6:** Multivariate analysis of the predictors of mortality (dead vs alive) by fitting a multiple logistic regression model with the generalized estimating equations (GEE) method (assuming an “independence” working correlation structure)*

Covariate	Estimated regression coefficient	Standard error	χ^2^ value	*P*-value	Estimated OR	95% CI of OR
Intercept	<0.0001	1.3367	<0.0001	1.0000	1.0000	0.0725-13.7862
Lymphocyte count mean or median >0.87 × 10^9^/L	-2.8551	1.2055	7.0560	0.0079	0.0576	0.0043-0.4726
Age mean or median†>67.28 years	2.8551	1.2055	7.0560	0.0079	17.3756	2.1157-232.7834

OR – odds ratio, CI – confidence interval

*Goodness-of-fit assessment – number of studies n = 19, number of arms *m* = 38 (cluster size = 2), the estimated area under the receiver operating characteristic (ROC) curve = 0.954 > 0.7 with 95% CI = 0.888-1 (DeLong). Yet, the regression coefficients and the standard errors were estimated by fitting the corresponding Firth’s bias-reduced logistic regression model with the logistf function of the logistf package in R due to the separation (or high discrimination) problem.

†Although “age mean or median” beat “age mean or median >67.28 years” in Run 6 of **Table 5** (AUC of ROC: 0.913 vs 0.895), we chose “age mean or median >67.28 years” in this multiple logistic regression model because the GAM plot of “age mean or median” indicated that its effect on logit (probability of being dead) decreased for age’s mean or median >77.5 years.

## DISCUSSION

The results of this study provide several imperative insights. After extensive comparisons, lymphocyte count was the predictor with the highest discrimination power among the 13 laboratory items explored for COVID-19 patients in 2020, while the mass vaccination program was not yet started. A single laboratory variable, lymphocyte count in initial hospital presentations, together with age, can be remarkable indicators to discriminate health consequences. To the best of our knowledge, this was the first meta-analytical study in which the potential bias of subject duplication of COVID-19 patients between studies had been eliminated before meta-analysis.

The SARS-CoV2 infection triggers multiple defensive mechanisms of the human body, including the immune responses (eg, WBC, lymphocytes, and neutrophils), inflammatory cataracts (eg, CRP and PCT), and activation of coagulation cascades (eg, platelet count and D-dimer) [29-32]. As the virus invades tissues, which starts early, the inflammation situation intensifies [31,33], and thus the values of inflammatory indicators will increase dramatically [11,34,35]. The widely distributed COVID-19 receptors, such as the angiotensin-converting enzyme-2 (ACE2) receptors, are abundantly expressed in a variety of cells residing in many organs, and they can exaggerate systemic failure due to direct organ injury [36,37]. Organ damage indicators, such as ALT, AST, total bilirubin, LDH, and hypersensitive troponin I, reflect the impairment situation accordingly [15,38,39]. Our study confirmed again that several laboratory variables are profound predictors of disease severity or mortality for patients with COVID-19, although they were not so intense as compared with lymphocyte count.

It is no surprise that lymphocyte count played such an important role in SARS-CoV-2 defence in patients with COVID-19 [14,30,33]. Adaptive immune cells, such as lymphocytes, are essential for virus clearance and recovery from the disease [30,33,40]. The interaction between SARS-CoV-2 and the immune system of an individual results in a variety of clinical manifestations [33]. This meta-analytical study revealed that lymphocyte count is a critical defensive characteristic in patients with COVID-19. An extremely lower level (ie, ≤0.87 × 10^9^/L) of lymphocyte counts (**Table 6**) implies immune weakness, and it would worsen the prognosis of patients with COVID-19. The immunopathological role of lymphocytes in the COVID-19 has been studied through diverse approaches, including a series of testing [33,41], subsets study [42], and meta-analysis [14]. It is essential to understand more about the interaction of SARS-CoV-2 with the host immune system and its subsequent contribution to disease progression and organ dysfunction. How to maintain or improve good immunity levels in daily life is also crucial for stakeholders facing life-threatening infectious diseases such as COVID-19. After all there is a certain portion of people refused to take vaccination or not fully vaccinated due to various reasons.

Many tools (eg, demographics, symptoms, vital signs, comorbidities, laboratory tests, imaging examinations, etc.) have been explored to determine their ability to predict disease prognoses for patients with COVID-19 [11-13,34,35,38,39]. Routine laboratory tests have several unique advantages. They quickly reveal the whole-body situation of a COVID-19 patient, whose physical and mental functions can change dramatically in a short period [43]. And, they are easy to access comparatively, repeatable, self-explanatory, and relatively inexpensive, and thus can be a cost-effective tool for monitoring infected patients in pandemic circumstance. Current criteria for judging severity and triaging or referring patients with COVID-19 are based on imaging, demographics, comorbidities, and vital signs or symptoms [44,45]. According to the results of this study, the lymphocyte count at administration plus age can be very useful indicators for those purposes.

The impact would be great if the 30 articles with concerns of duplicate subjects were not eliminated prior to the meta-analysis. In all, only 76 articles were included in the final analysis. Calculating (the number of study subjects) × (number of laboratory data items reported) to maximize the desired information seemed a good way to deal with this situation. The AUC of ROC decreased (**Table 1**, **Table 3** and **Table 5**) while more available studies participated in sequential runs of simple logistic regression analysis. The reason to explain was that more studies included in an analysis increased the diversity of the pool. Regions, ethnic groups, time periods, patient groups, and even virus strains varied between all studies.

### Limitations

Interpretation of results should be cautious due to the lack of unpublished articles, non-English articles, and paediatric studies. Ideally, all desired laboratory data should be collected and analysed. However, it is not realistic in the real world. We suggest collecting essential data through a standardized list, while clinical presentation, medical history, imaging information, comprehensive laboratory data, and other valuable factors can be assembled and analysed to accelerate knowledge accumulation, especially under global pandemics. Retrospective observational studies were conducted at the hospital or community level so that the characteristics of individual patients could not be retrieved. In addition, the dynamic relationship among various laboratory data, functions, and feelings of the patient had not been explored due to the lack of data. More extensive and large-scale studies of individual patients are still required to verify the findings of this study.

## CONCLUSIONS

This meta-analytical study involving 26 627 laboratory-confirmed COVID-19 patients from 16 countries before mass vaccination, provided shreds of evidence on the defence of SARS-CoV2 infection during the ongoing COVID-19 pandemic. We found that lymphocyte count is the most important biomarker for monitoring disease severity and mortality. Lymphocyte count should be monitored for the prognoses of COVID-19 patients in clinical settings in particular for patients not fully vaccinated.

## Additional material


Online Supplementary Document
