# Supplementary material for: Lymphocyte count is a universal predictor of health outcomes in COVID-19 patients before mass vaccination: A meta-analytical study
Source: J Glob Health. 2022 Sep 17;12:05041. doi: 10.7189/jogh.12.05041 (PMC9480861; doi:10.7189/jogh.12.05041)
Supplement: Online Supplementary Document [file jogh-12-05041-s001.pdf]

Supplementary Document

Table S1. The profile of the collected 76 studies with a total of 26,627 laboratory-confirmed COVID-19 patients

Study	Country	Recruitment Period†	Total	Non-Severe	Severe	Non-Critical Severe	Critical Severe	Alive	Dead
Almazeedi Sulaiman et al. [1]*	Kuwait	Feb 24-Apr 20	1096	–	–	1054	42	1077	19
Bahl Amit et al. [2]	USA	Mar 1-Mar 31	1461	–	–	–	–	1134	327
Berenguer Juan et al. [3]	Spain	Feb-Mar 17	4035	–	–	–	–	2904	1131
Bonetti Graziella et al. [4]	Italy	Mar 1-Mar 30	144	–	–	–	–	74	70
Botero Diana Maria Ronderos et al. [5]	USA	Mar 15-Apr 23	157	–	–	–	–	132	25
Brill Simon E et al. [6]	UK	Mar 10-Apr 8	410	–	–	–	–	237	173
Cai Qingxian et al. [7]	China	Jan 11-Feb 6	298	240	58	–	–	–	–
Chen Lei et al. [8]	China	Jan 20-Apr 4	1859	–	–	–	–	1651	208
Chen Tao et al. [9]	China	Jan 13-Feb 12	274	–	–	–	–	161	113
Ciceri Fabio et al. [10]	Italy	Feb 25-Mar 24	410	–	–	–	–	315	95
Cipriani Alberto et al. [11]	Italy	Feb 26-Mar 31	109	–	–	–	–	89	20
Fan Bingwen Eugene et al. [12]	Singapore	Jan 23-Feb 4	67	–	–	58	9	–	–
Ferguson Jessica et al. [13]	USA	Mar 13-Apr 11	72	–	–	51	21	–	–
Gao Yong et al. [14]	China	Jan 23-Feb 2	43	28	15	–	–	–	–
Gayam Vijay et al. [15]	USA	Mar 1-Apr 9	408	–	–	–	–	276	132
Ghweil Ali A et al. [16]	Egypt	Mar-Apr	66	36	30	–	–	–	–
Giacomelli Andrea et al. [17]	Italy	Feb 21-Mar 19	233	–	–	–	–	185	48
He Li et al. [18]	China	Feb 10-Apr 9	174	120	54	–	–	–	–
Hong Kyung Soo et al. [19]	South Korea	–	98	–	–	85	13	–	–
Huang Chaolin et al. [20]	China	Dec 16 '19-Jan 2	41	–	–	28	13	–	–
Huang Huihuang et al. [21]	China	Jan 13-Mar 10	64	43	21	–	–	–	–
Israelsen Simone Bastrup et al. [22]	Denmark	Mar 10-Apr 23	175	–	–	148	27	–	–
Javannian Mostafa et al. [23]	Iran	Feb 25-Mar 12	100	–	–	–	–	81	19
Jiang Hao et al. [24]	China	Feb-Mar	59	–	–	15	44	–	–

Khalil K et al. [24]	UK	Mar 7-Apr 7	220	–	–	–	–	162	58
Khamis Faryal et al. [26]	Oman	Feb 24-Apr 24	63	–	–	39	24	–	–
Lee Ji Yeon et al. [27]	South Korea	Feb 18-Mar 4	98	–	–	–	–	78	20
Lee Ji Yeon et al. [28]	South Korea	Feb 21-Apr 2	694	557	137	–	–	–	–
Lendorf Maria Elisabeth et al. [29]	Denmark	Mar 1-May 4	111	–	–	91	20	–	–
Li Huan et al. [30]	China	Jan 18-Feb 26	132	60	72	116	16	–	–
Li Kunhua et al. [31]	China	Jan -Feb	83	58	25	–	–	–	–
Li Tao et al. [32]	China	Feb 1-Mar 31	312	207	105	–	–	–	–
Li Xiaolei et al. [33]	China	Jan 24-Mar 12	215	159	56	–	–	–	–
Liu Jiao et al. [34]	China	Dec 29 ‘19-Feb 28	1190	–	–	–	–	1033	157
Liu Songqiao et al. [35]	China	Jan 10-Mar 15	625	561	64	–	–	–	–
Lv Zhihua et al. [36]	China	Feb 4-Feb 28	354	115	239	270	84	–	–
Mao Ling et al. [37]	China	Jan 16-Feb 19	214	126	88	–	–	–	–
Ortiz-Brizuela Edgar et al. [38]	Mexico	Feb 26-Apr 11	140	–	–	111	29	–	–
Qian Guo-Qing et al. [39]	China	Jan 20-Feb 11	91	82	9	–	–	–	–
Qun Sen et al. [40]	China	Feb 2-Mar 17	230	–	–	225	5	–	–
Romero-Sanchez Carlos M et al. [41]	Spain	Mar 1-Apr 1	841	512	329	–	–	–	–
Satış Hasan et al. [42]	Turkey	–	38	27	11	–	–	–	–
Shahriarirad Reza et al. [43]	Iran	Feb 20-Mar 20	113	–	–	102	11	104	9
Shang Weifeng et al. [44]	China	Jan 16-Feb 28	443	304	139	–	–	–	–
Shu Zixin et al. [45]	China	Jan 15-Mar 2	293	207	86	–	–	–	–
Sun Dong et al. [46]	China	Jan 23-Feb 19	84	61	23	–	–	–	–
Sun Haiying et al. [47]	China	Jan 29-Mar 5	244	–	–	–	–	123	121
Sun Suyu et al. [48]	China	Jan 19-Feb 20	116	89	27	–	–	–	–
Sun Ying et al. [49]	China	Jan-Apr	63	–	–	54	9	–	–
Taghiloo Saeid et al. [50]	Iran	Mar-Apr	61	39	22	–	–	–	–
Tao Zheying et al. [51]	China	Dec 1 ‘19-Mar	222	202	20	–	–	–	–
Uchida Yoshihito et al. [52]	Japan	Mar-Jun	35	27	8	–	–	–	–
Vena Antonio et al. [53]	Italy	Feb 25-Mar 25	275	–	–	–	–	155	120
Wang Changzheng et al. [54]	China	Jan 23-Feb 13	161	131	30	–	–	–	–
Wang Dan et al. [55]	China	Jan 15-Feb 28	143	72	71	–	–	–	–
Wang Dawei et al. [56]	China	Jan 1-Jan 28	138	–	–	102	36	–	–
Wang Feng et al. [57]	China	–	65	–	–	50	15	–	–
Wang Ruirui et al. [58]	China	Jan 22-Feb 18	125	100	25	–	–	–	–

Wang Ruoran et al. [59]	China	Jan 30-Feb 24	450	–	–	–	–	372	78
Wu J et al. [60]	China	Jan 20-Feb 19	280	197	83	–	–	–	–
Yan Xiquan et al. [61]	China	Jan 21-Jun 27	194	156	38	180	14	–	–
Yang Luhuan et al. [62]	China	Jan 30-Feb 8	200	–	–	171	29	–	–
Yang Miao et al. [63]	China	Jan 17-Mar 12	108	84	24	–	–	–	–
Yao Qingchun et al. [64]	China	Jan 30-Feb 11	96	83	13	–	–	–	–
Ye Wenjing et al. [65]	China	Jan 1-Mar 16	349	–	–	–	–	297	52
Yu Caizheng et al. [66]	China	Jan 14-Feb 28	1663	799	864	–	–	–	–
Zerah Lorene et al. [67]	France	Mar 13-Apr 15	821	–	–	–	–	571	250
Zhang Chi et al. [68]	China	Jan-Feb	80	56	21	–	–	77	3
Zhang Jin-Jin et al. [68]	China	Jan 16-Feb 3	140	82	58	–	–	–	–
Zhang Jin-Jin et al. [70]	China	Dec 29 '19-Feb 16	289	–	–	–	–	240	49
Zhang Shan-Yan et al. [71]	China	Jan 17-Feb 12	788	710	78	771	17	–	–
Zhang Wenjing et al. [72]	China	Jan-Mar	90	70	20	–	–	–	–
Zhao Zirun et al. [73]	USA	Mar 9-Apr 20	593	–	–	398	195	–	–
Zheng Fang et al. [74]	China	Jan 17-Feb 7	161	131	30	–	–	–	–
Zheng Yongli et al. [75]	China	Jan 16-Feb 20	99	67	32	–	–	–	–
Zheng Yufen et al. [76]	China	Jan 17-Feb 26	141	112	29	–	–	–	–
**Sub-total**			**–**	**9,764**		**4,792**		**14,825**	
**Total**			**26,627**	**6,710**	**3,054**	**4,119**	**673**	**11,528**	**3,297**

* The details can be found in the online *References for Supplementary Table S1*.
† The subjects were recruited during the year 2020 if not specified.

References for Supplementary Table S1

1. Almazeedi S, Al-Youha S, Jamal M, et al. Characteristics, risk factors and outcomes among the first consecutive 1096 patients diagnosed with COVID-19 in Kuwait. EClinicalMedicine 2020;24:100448. https://doi.org/10.1016/j.eclinm.2020.100448
2. Bahl A, Van Baalen MN, Ortiz L, et al. Early predictors of in-hospital mortality in patients with COVID-19 in a large American cohort. Intern Emerg Med 2020;15(8):1485-1499. doi:10.1007/s11739-020-02509-7

3. Berenguer J, Ryan P, Rodríguez-Baño J, et al. Characteristics and predictors of death among 4035 consecutively hospitalized patients with COVID-19 in Spain. Clin Microbiol Infect 2020;26(11):1525-1536. doi:10.1016/j.cmi.2020.07.024
4. Bonetti G, Manelli F, Patroni A, et al. Laboratory predictors of death from coronavirus disease 2019 (COVID-19) in the area of Valcamonica, Italy. Clin Chem Lab Med 2020;58(7):1100-1105. doi:10.1515/cclm-2020-0459
5. Botero DMR, Omar AMS, Sun HK, et al. Covid-19 in the healthy patient population demographic and clinical phenotypic characterization and predictors of in-hospital outcomes. Arterioscler Thromb Vasc Biol 2020;40(11):2764-2775. doi:10.1161/ATVBAHA.120.314845
6. Brill SE, Jarvis HC, Ozcan E, et al. COVID-19: A retrospective cohort study with focus on the over-80s and hospital-onset disease. BMC Med 2020;18(1). doi:10.1186/s12916-020-01665-z
7. Cai Q, Huang D, Ou P, et al. COVID-19 in a designated infectious diseases hospital outside Hubei Province, China. Allergy Eur J Allergy Clin Immunol 2020;75(7):1742-1752. doi:10.1111/all.14309
8. Chen L, Yu J, He W, et al. Risk factors for death in 1859 subjects with COVID-19. Leukemia 2020;34:2173–2183. https://doi.org/10.1038/s41375-020-0911-0
9. Chen T, Wu D, Chen H, et al. Clinical characteristics of 113 deceased patients with coronavirus disease 2019: Retrospective study. BMJ 2020;368:m1091. doi:10.1136/bmj.m1091
10. Ciceri F, Castagna A, Rovere-Querini P, et al. Early predictors of clinical outcomes of COVID-19 outbreak in Milan, Italy. Clin Immunol 2020;217:108509. doi:10.1016/j.clim.2020.108509.
11. Cipriani A, Capone F, Donato F, et al. Cardiac injury and mortality in patients with Coronavirus disease 2019 (COVID-19): Insights from a mediation analysis. Intern Emerg Med 2021;16(2):419-427. doi:10.1007/s11739-020-02495-w
12. Fan BE, Chong VCL, Chan SSW, et al. Hematologic parameters in patients with COVID-19 infection. Am J Hematol 2020;95(6):E131-E134. doi:10.1002/ajh.25774

13. Ferguson J, Rosser J, Quintero O, et al. Characteristics and outcomes of coronavirus disease patients under nonsurge conditions, Northern California, USA, March–April 2020. Emerg Infect Dis 2020;26(8):1679-1685. doi:10.3201/eid2608.201776
14. Gao Y, Li T, Han M, et al. Diagnostic utility of clinical laboratory data determinations for patients with the severe COVID-19. J Med Virol 2020;92(7):791-796. doi:10.1002/jmv.25770
15. Gayam VR, Ditah C, Lamichhane S. Clinical characteristics and predictors of mortality in African-Americans with COVID-19 from an inner-city community teaching hospital in New York. Artic J Med Virol 2020;93(2):812-819. doi:10.1002/jmv.26306
16. Ghweil AA, Hassan MH, Khodeary A, et al. Characteristics, outcomes and indicators of severity for covid-19 among sample of ESNA quarantine hospital's patients, Egypt: A retrospective study. Infect Drug Resist 2020;13:2375-2383. doi:10.2147/IDR.S263489
17. Giacomelli A, Ridolfo A, Milazzo L, et al. 30-day mortality in patients hospitalized with COVID-19 during the first wave of the Italian epidemic: A prospective cohort study. Pharmacol Res 2020;158:104931. doi:10.1016/j.phrs.2020.104931
18. He L, Zhang Q, Li Z, et al. Incorporation of urinary neutrophil gelatinase-associated lipocalin and computed tomography quantification to predict acute kidney injury and in-hospital death in COVID-19 patients. Kidney Dis 2021;7:120-130. https://doi.org/10.1159/000511403
19. Hong K, Lee K, Chung J, et al. Clinical features and outcomes of 98 patients hospitalized with SARS-CoV-2 infection in Daegu, South Korea: A brief descriptive study. Yonsei Med J 2020;61(5):431-437. doi:10.3349/ymj.2020.61.5.431
20. Huang C, Wang Y, Li X, et al. Clinical features of patients infected with 2019 novel coronavirus in Wuhan, China. Lancet 2020;395(10223):497-506. https://doi.org/10.1016/S0140-6736(20)30183-5
21. Huang H, Song B, Xu Z, et al. Predictors of coronavirus disease 2019 severity: A retrospective study of 64 cases. Jpn J Infect Dis 2021;74(1):54-60. doi:10.7883/yoken.JJID.2020.298
22. Israelsen S, Kristiansen K, Hindsberger B, et al. Characteristics of patients with COVID-19 pneumonia at Hvidovre Hospital, March−April 2020. Dan Med J 2020;67(6):A05200313. PMID: 32448405

23. Javanian M, Bayani M, Shokri M, et al. Clinical and laboratory findings from patients with COVID-19 pneumonia in Babol North of Iran: A retrospective cohort study. Rom J Intern Med 2020;58(3):161-167. doi:10.2478/rjim-2020-0013
24. Jiang H, Guo W, Shi Z, et al. Clinical imaging characteristics of inpatients with coronavirus disease-2019 in Heilongjiang Province, China: A retrospective study. Aging (Albany, NY) 2020;12(14):13860-13868. doi:10.18632/aging.103633
25. Khalil K, Agbontaen K, McNally D, et al. Clinical characteristics and 28-day mortality of medical patients admitted with COVID-19 to a central London teaching hospital. J Infect 2020;81(3):e85-e89. doi:10.1016/j.jinf.2020.06.027
26. Khamis F, Al-Zakwani I, Al Naamani H, et al. Clinical characteristics and outcomes of the first 63 adult patients hospitalized with COVID-19: An experience from Oman. J Infect Public Health 2020;13(7):906-913. doi:10.1016/j.jiph.2020.06.002
27. Lee JY, Kim HA, Huh K, Hyun M, Rhee JY, Jang S, Kim JY, Peck KR, Chang HH. Risk factors for mortality and respiratory support in elderly patients hospitalized with COVID-19 in Korea. J Korean Med Sci 2020;35(23):e223. https://doi.org/10.3346/jkms.2020.35.e223
28. Lee JY, Hong SW, Hyun M, Park JS, Lee JH, Suh YS, Kim DH, Han SW, Cho CH, Kim HA. Epidemiological and clinical characteristics of coronavirus disease 2019 in Daegu, South Korea. Int J Infectious Disease 2020;98:462-466. https://doi.org/10.1016/j.ijid.2020.07.017
29. Lendorf ME, Boisen MK, Kristensen PL, et al. Characteristics and early outcomes of patients hospitalised for COVID-19 in North Zealand, Denmark. Dan Med J 2020;67(9):A06200428. PMID: 32800073
30. Li H, Xiang X, Ren H, et al. Serum amyloid A is a biomarker of severe coronavirus disease and poor prognosis. J Infect 2020;80(6):646-655. doi:10.1016/j.jinf.2020.03.035
31. Li K, Wu J, Wu F, et al. The clinical and chest CT features associated with severe and critical COVID-19 pneumonia. Invest Radiol 2020;55(6):327-331. doi:10.1097/RLI.0000000000000672
32. Li T, Lu L, Zhang W, et al. Clinical characteristics of 312 hospitalized older patients with COVID-19 in Wuhan, China. Arch Gerontol Geriatr 2020;91:104185. doi:10.1016/j.archger.2020.104185

33. Li X, Liu Y, Li J, et al. Immune characteristics distinguish patients with severe disease associated with SARS-CoV-2. Immunol Res 2020;68(6):398-404. doi:10.1007/s12026-020-09156-2
34. Liu J, Zhang S, Wu Z, et al. Clinical outcomes of COVID-19 in Wuhan, China: A large cohort study. Ann Intensive Care 2020;10(1):1-21. doi:10.1186/S13613-020-00706-3
35. Liu S, Luo H, Wang Y, et al. Clinical characteristics and risk factors of patients with severe COVID-19 in Jiangsu Province, China: A retrospective multicentre cohort study. BMC Infect Dis 2020;20(1):584. doi:10.1186/s12879-020-05314-x
36. Lv Z, Cheng S, Le J, et al. Clinical characteristics and co-infections of 354 hospitalized patients with COVID-19 in Wuhan, China: A retrospective cohort study. Microbes Infect 2020;22(4-5):195-199. doi:10.1016/j.micinf.2020.05.007
37. Mao L, Jin H, Wang M, et al. Neurologic manifestations of hospitalized patients with coronavirus disease 2019 in Wuhan, China. JAMA Neurol 2020;77(6):683-690. doi:10.1001/jamaneurol.2020.1127
38. Ortiz-Brizuela E, Villanueva-Reza M, González-Lara MF, et al. Clinical and epidemiological characteristics of patients diagnosed with COVID-19 in a tertiary care center in Mexico City: A prospective cohort study. Rev Invest Clin 2020;72(3):165-177. doi:10.24875/RIC.20000211
39. Qian GQ, Yang NB, Ding F, et al. Epidemiologic and clinical characteristics of 91 hospitalized patients with COVID-19 in Zhejiang, China: A retrospective, multi-centre case series. QJM 2020;113(7):474-481. doi:10.1093/qjmed/hcaa089
40. Qun S, Wang Y, Chen J, et al. Neutrophil-to-lymphocyte ratios are closely associated with the severity and course of non-mild COVID-19. Front Immunol 2020;11:2160. doi:10.3389/fimmu.2020.02160
41. Romero-Sánchez CM, Díaz-Maroto I, Fernández-Díaz E, et al. Neurologic manifestations in hospitalized patients with COVID-19: The ALBACOVID registry. Neurology 2020;95(8):e1060-e1070. doi:10.1212/WNL.0000000000009937
42. Satış H, Özger HS, Aysert Yıldız P, et al. Prognostic value of interleukin-18 and its association with other inflammatory markers and disease severity in COVID-19. Cytokine 2021;137:155302. doi:10.1016/j.cyto.2020.155302

43. Shahriarirad R, Shahriarirad R, Khodamoradi Z, et al. Epidemiological and clinical features of 2019 novel coronavirus diseases (COVID-19) in the South of Iran. BMC Infect Dis 2020;20(1):427. doi:10.1186/s12879-020-05128-x
44. Shang W, Dong J, Ren Y, et al. The value of clinical parameters in predicting the severity of COVID-19. J Med Virol 2020;92(10):2188-2192. doi:10.1002/jmv.26031
45. Shu Z, Zhou Y, Chang K, et al. Clinical features and the traditional Chinese medicine therapeutic characteristics of 293 COVID-19 inpatient cases. Front Med 2020;14(6):760-775. doi:10.1007/s11684-020-0803-8
46. Sun D, Li X, Guo D, et al. CT quantitative analysis and its relationship with clinical features for assessing the severity of patients with COVID-19. Korean J Radiol 2020;21(7):859-868. doi:10.3348/kjr.2020.0293
47. Sun H, Ning R, Tao Y, et al. Risk factors for mortality in 244 older adults with COVID-19 in Wuhan, China: A retrospective study. J Am Geriatr Soc 2020;68(6):E19-E23. doi:10.1111/jgs.16533
48. Sun S, Cai X, Wang H, et al. Abnormalities of peripheral blood system in patients with COVID-19 in Wenzhou, China. Clin Chim Acta 2020;507:174-180. doi:10.1016/j.cca.2020.04.024
49. Sun Y, Dong Y, Wang L, et al. Characteristics and prognostic factors of disease severity in patients with COVID-19: The Beijing experience. J Autoimmun 2020;112:102473. doi:10.1016/j.jaut.2020.102473
50. Taghiloo S, Aliyali M, Abedi S, et al. Apoptosis and immunophenotyping of peripheral blood lymphocytes in Iranian COVID-19 patients: Clinical and laboratory characteristics. J Med Virol 2021;93(3):1589-1598. doi:10.1002/jmv.26505
51. Tao Z, Xu J, Chen W, et al. Anemia is associated with severe illness in COVID-19: A retrospective cohort study. J Med Virol 2021;93(3):1478-1488. doi:10.1002/jmv.26444
52. Uchida Y, Uemura H, Yamaba S, et al. Significance of liver dysfunction associated with decreased hepatic CT attenuation values in Japanese patients with severe COVID-19. J Gastroenterol 2020;55(11):1098-1106. doi:10.1007/s00535-020-01717-4
53. Vena A, Giacobbe DR, Di Biagio A, et al. Clinical characteristics, management and in-hospital mortality of patients with coronavirus disease 2019 in Genoa, Italy. Clin Microbiol Infect 2020;26(11):1537-1544. doi:10.1016/j.cmi.2020.07.049

54. Wang C, Deng R, Gou L, et al. Preliminary study to identify severe from moderate cases of COVID-19 using combined hematology parameters. Ann Transl Med 2020;8(9):593-593. doi:10.21037/atm-20-3391
55. Wang D, Li R, Wang J, et al. Correlation analysis between disease severity and clinical and biochemical characteristics of 143 cases of COVID-19 in Wuhan, China: A descriptive study. BMC Infect Dis 2020;20(1):519. doi:10.1186/s12879-020-05242-w
56. Wang D, Hu B, Hu C, et al. Clinical characteristics of 138 hospitalized patients with 2019 novel coronavirus-infected pneumonia in Wuhan, China. JAMA 2020;323(11):1061-1069. doi:10.1001/jama.2020.1585
57. Wang F, Hou H, Luo Y, et al. The laboratory tests and host immunity of COVID-19 patients with different severity of illness. JCI Insight 2020;5(10). doi:10.1172/JCI.INSIGHT.137799
58. Wang R, Pan M, Zhang X, et al. Epidemiological and clinical features of 125 hospitalized patients with COVID-19 in Fuyang, Anhui, China. Int J Infect Dis 2020;95:421-428. doi:10.1016/j.ijid.2020.03.070
59. Wang R, He M, Yin W, et al. The prognostic nutritional index is associated with mortality of COVID-19 patients in Wuhan, China. J Clin Lab Anal 2020;34(10):e23566. doi:10.1002/jcla.23566
60. Wu J, Li W, Shi X, et al. Early antiviral treatment contributes to alleviate the severity and improve the prognosis of patients with novel coronavirus disease (COVID-19). J Intern Med 2020;288(1):128-138. doi:10.1111/joim.13063
61. Yan X, Han X, Peng D, et al. Clinical characteristics and prognosis of 218 patients with COVID-19: A retrospective study based on clinical classification. Front Med 2020;7:485. doi:10.3389/fmed.2020.00485
62. Yang L, Liu J, Zhang R, et al. Epidemiological and clinical features of 200 hospitalized patients with corona virus disease 2019 outside Wuhan, China: A descriptive study. J Clin Virol 2020;129:104475. doi:10.1016/j.jcv.2020.104475
63. Yang M, Chen X, Xu Y. A retrospective study of the C-reactive protein to lymphocyte ratio and disease severity in 108 patients with early COVID-19 Pneumonia from January to March 2020 in Wuhan, China. Med Sci Monit 2020;26:e926393-1. doi:10.12659/MSM.926393

64. Yao Q, Wang P, Wang X, et al. A retrospective study of risk factors for severe acute respiratory syndrome coronavirus 2 infections in hospitalized adult patients. Polish Arch Intern Med 2020;130(5):390-399. doi:10.20452/pamw.15312
65. Ye W, Chen G, Li X, et al. Dynamic changes of D-dimer and neutrophil-lymphocyte count ratio as prognostic biomarkers in COVID-19. Respir Res 2020;21(1):169. doi:10.1186/s12931-020-01428-7
66. Yu C, Lei Q, Li W, Wang X, Li W, Liu W. Epidemiological and clinical characteristics of 1663 hospitalized patients infected with COVID-19 in Wuhan, China: A single-center experience. J Infect Public Health 2020;13(9):1202-1209. doi:10.1016/j.jiph.2020.07.002
67. Zerah L, Baudouin É, Pépin M, et al. Clinical characteristics and outcomes of 821 older patients with SARS-Cov-2 infection admitted to acute care geriatric wards. J Gerontol A Biol Sci Med Sci 2021;76(3):e4-e12. doi:10.1093/gerona/glaa210
68. Zhang C, Qin L, Li K, et al. A novel scoring system for prediction of disease severity in COVID-19. Front Cell Infect Microbiol 2020;10:318. doi:10.3389/fcimb.2020.00318
69. Zhang JJ, Dong X, Cao YY, et al. Clinical characteristics of 140 patients infected with SARS-CoV-2 in Wuhan, China. Allergy Eur J Allergy Clin Immunol. 2020;75(7):1730-1741. doi:10.1111/all.14238
70. Zhang JJ, Cao YY, Tan G, et al. Clinical, radiological, and laboratory characteristics and risk factors for severity and mortality of 289 hospitalized COVID-19 patients. Allergy Eur J Allergy Clin Immunol 2021;76(2):533-550. doi:10.1111/all.14496
71. Zhang SY, Lian JS, Hu JH, et al. Clinical characteristics of different subtypes and risk factors for the severity of illness in patients with COVID-19 in Zhejiang, China. Infect Dis Poverty 2020;9(1):85. doi:10.1186/s40249-020-00710-6
72. Zhang W, Li L, Liu J, et al. The characteristics and predictive role of lymphocyte subsets in COVID-19 patients. Int J Infect Dis 2020;99:92-99. doi:10.1016/j.ijid.2020.06.079
73. Zhao Z, Chen A, Hou W, et al. Prediction model and risk scores of ICU admission and mortality in COVID-19. PLoS One 2020;15(7):e0236618. doi:10.1371/journal.pone.0236618

74. Zheng F, Tang W, Li H, et al. Clinical characteristics of 161 cases of corona virus disease 2019 (COVID-19) in Changsha. Eur Rev Med Pharmacol Sci 2020;24(6):3404-3410. doi:10.26355/eurrev_202003_20711
75. Zheng Y, Xu H, Yang M, et al. Epidemiological characteristics and clinical features of 32 critical and 67 noncritical cases of COVID-19 in Chengdu. J Clin Virol 2020;127:104366. doi:10.1016/j.jcv.2020.104366
76. Zheng Y, Zhang Y, Chi H, Chen S, Peng M, Luo L, Chen L, Li J, Shen B, Wang D. The hemocyte counts as a potential biomarker for predicting disease progression in COVID-19: A retrospective study. Clin Chem Lab Med 2020;58(7):1106-1115. doi:10.1515/cclm-2020-0377

Table S2. The summary statistics of the demographics, clinical characteristics, comorbidities, and laboratory data of the COVID-19 patients on initial hospital presentations from the collected 38 studies for the assessment of severity

Variable	Number of Studies* (*n*)	Non-Severe			Severe			*P* Value†
		Median	Minimum	Maximum	Median	Minimum	Maximum	
**Demographics**								
Age (years)	38	46.50	37.55	67.10	62.55	39.47	75.00	<0.0001
Male (%)	35	50.40	27.80	77.80	60.00	38.10	75.00	0.0018
Current smoker (%)	7	6.58	0	36.10	8.98	0	30.00	0.5649
**Vital signs**								
Heart rate (beats/min)	4	86.42	82.00	89.00	89.99	86.00	92.00	0.1465
Respiratory rate (breaths/min)	3	20.00	18.87	20.00	20.98	20.00	21.00	0.1642
Systolic pressure (mmHg)	2	108.15	93.30	123.00	111.95	91.00	132.90	1
**Clinical features**								
Fever (%)	26	73.36	16.80	97.20	89.75	38.30	100	0.0030
Cough (%)	26	58.97	31.50	81.50	69.60	15.00	100	0.0570
Fatigue (%)	17	25.71	4.17	76.10	40.00	5.17	87.50	0.3394
Myalgia (%)	15	14.21	2.00	43.50	23.53	0	51.81	0.2290
Dyspnea (%)	11	11.90	1.65	44.40	30.00	2.15	80.00	0.0417
Sputum production (%)	14	23.70	6.90	88.10	37.55	9.00	96.20	0.1636
Headache (%)	17	10.30	1.20	25.60	13.30	0	38.55	0.4484
Sore throat (%)	12	14.11	0.83	26.10	10.84	0	46.70	0.7949
Diarrhea (%)	19	8.21	0.51	28.20	7.62	0	50.00	0.9070

Shortness of breath (%)	5	22.86	6.90	45.80	25.00	8.00	100	0.5476
Chest congestion / Chest tightness (%)	5	29.00	4.50	40.30	45.30	0	50.00	0.4206
Chest pain (%)	6	2.10	1.02	15.80	13.42	2.15	20.00	0.0931
**Comorbidities**								
Any (%)	14	32.10	6.90	70.50	58.27	31.38	90.50	0.0033
Hypertension (%)	27	14.80	2.90	48.00	37.90	4.30	82.90	<0.0001
Diabetes (%)	28	8.30	0	34.80	16.30	6.70	46.70	<0.0001
Cardiovascular disease (%)	26	4.68	0	22.70	12.75	0	47.37	0.0014
Chronic obstructive pulmonary disease (%)	21	1.74	0	7.73	6.70	0	24.00	0.0003
Malignancy (%)	16	1.63	0	7.40	4.46	0	10.30	0.0328
Cerebrovascular disease (%)	11	2.80	0.94	7.50	7.90	0	25.91	0.0086
Chronic liver disease (%)	15	3.10	0	8.33	4.50	0	20.80	0.2444
Chronic renal disease (%)	16	0.84	0	6.70	4.61	1.70	13.00	0.0006
**Blood routine**								
White blood cell count ($\times 10^9$/L)	36	5.00	3.60	8.59	5.75	3.40	12.10	0.0047
Neutrophil count ($\times 10^9$/L)	28	3.22	2.40	6.69	4.43	1.65	10.51	0.0011
Lymphocyte count ($\times 10^9$/L)	36	1.30	0.80	1.90	0.81	0.48	2.19	<0.0001
Neutrophil/Lymphocyte ratio (NLR)	4	2.49	2.38	7.93	8.15	4.75	29.90	0.1143
Platelet count ($\times 10^9$/L)	26	206.00	156.00	240.46	176.00	86.00	265.00	0.0077
**Blood biochemistry**								
Alanine aminotransferase (ALT) (U/L)	25	23.00	18.00	40.53	28.54	19.90	54.00	0.0016
Aspartate aminotransferase (AST) (U/L)	23	25.00	16.00	42.60	34.00	26.00	61.00	0.0003
Total bilirubin (μmol/L)	15	9.80	6.60	13.70	11.30	6.70	21.20	0.0128
Lactate dehydrogenase (LDH) (U/L)	17	209.20	150.40	489.00	287.00	220.10	695.19	0.0001

**Coagulation function**								
D-dimer (mg/L or μg/mL)	18	0.40	0.20	1.90	0.93	0.30	7.93	0.0011
**Inflammatory markers**								
C-reactive protein (CRP) (mg/L)	29	9.70	1.70	106.66	53.80	19.50	291.00	<0.0001
Procalcitonin (PCT) (×100 ng/mL)	22	4.65	0.13	162.00	1.00	0	331.00	0.0280
**Myocardial injury marker**								
Hypersensitive troponin I (hs-cTnI) (×100 ng/mL)	3	0.75	0.40	1.00	1.53	0.70	5.00	0.4000

* It was the number of studies without missing values among the collected 38 studies.

† The listed *P* values were calculated using the Wilcoxon rank-sum test based on the available number of studies.

Table S3. The summary statistics of the demographics, clinical characteristics, and biomarkers of the COVID-19 patients on initial hospital presentations from the collected 21 studies for the assessment of critical severity

Variable	Number of Studies* (*n*)	Non-Critically Severe			Critically Severe			*P* Value†
		Median	Minimum	Maximum	Median	Minimum	Maximum	
**Demographics**								
Age (years)	19	52.00	37.10	73.00	63.20	49.00	71.00	0.0030
Male (%)	18	53.78	37.60	82.00	63.80	46.20	88.00	0.0095
Current smoker (%)	5	10.96	6.71	21.90	11.80	0	26.70	1
**Vital signs**								
Heart rate (beats/min)	5	87.00	84.90	98.00	89.00	83.50	100	0.6742
Respiratory rate (breaths/min)	6	20.45	20.00	22.00	22.50	20.00	30.00	0.1832
Systolic pressure (mmHg)	6	125.15	90.00	130.00	126.05	91.00	145.00	0.6304
**Clinical features**								
Fever (%)	15	73.93	51.00	98.00	77.80	45.00	100	0.6630
Cough (%)	15	64.03	27.60	90.10	66.70	45.00	100	1
Fatigue (%)	11	26.00	3.30	65.70	35.30	7.10	90.90	0.4779
Myalgia (%)	14	24.95	6.70	75.50	25.25	9.00	79.30	0.9100
Dyspnea (%)	8	35.00	4.10	61.50	73.40	20.00	92.00	0.0086
Sputum production (%)	10	25.25	1.70	48.74	20.20	9.70	86.00	0.9698
Headache (%)	13	14.60	5.90	80.18	9.00	0	62.07	0.3048
Sore throat (%)	10	14.42	9.50	32.40	11.75	0	33.30	0.3642
Diarrhea (%)	12	9.01	1.40	33.30	10.15	0	37.90	0.6441

Shortness of breath (%)	7	21.00	1.70	74.00	66.67	28.60	95.00	0.0530
Chest congestion / Chest tightness (%)	1	15.80	15.80	15.80	15.40	15.40	15.40	1
Chest pain (%)	3	33.30	11.80	36.30	54.50	9.50	55.20	0.7000
**Comorbidities**								
Any (%)	8	31.12	26.22	67.00	58.75	33.33	100	0.0104
Hypertension (%)	14	25.50	14.00	42.70	40.60	15.00	88.20	0.0094
Diabetes (%)	15	12.70	5.90	26.10	23.50	8.00	46.00	0.0128
Cardiovascular disease (%)	16	11.30	1.32	51.40	17.00	0	60.00	0.2351
Chronic obstructive pulmonary disease (%)	14	2.68	0	9.00	5.75	0	36.00	0.1901
Malignancy (%)	12	1.90	0.74	8.00	1.20	0	11.10	0.4322
Cerebrovascular disease (%)	5	1.34	0.57	12.00	10.00	0	21.00	0.3457
Chronic liver disease (%)	10	2.15	0	6.74	1.20	0	11.80	0.6456
Chronic renal disease (%)	10	2.55	0.60	7.70	7.00	0	15.00	0.0638
**Blood routine**								
White blood cell count ($\times 10^9$/L)	20	5.74	4.30	6.77	6.82	5.10	11.30	0.0001
Neutrophil count ($\times 10^9$/L)	13	3.21	1.70	4.64	5.54	3.50	10.60	0.0009
Lymphocyte count ($\times 10^9$/L)	17	1.14	0.86	1.60	0.80	0.40	1.37	0.0001
Neutrophil/Lymphocyte ratio (NLR)	1	2.50	2.50	2.50	6.32	6.32	6.32	1
Platelet count ($\times 10^9$/L)	15	193.00	149.00	245.00	196.00	142.00	253.00	0.9504
**Blood biochemistry**								
Alanine aminotransferase (ALT) (U/L)	15	30.10	23.00	73.02	37.80	24.76	89.10	0.0084
Aspartate aminotransferase (AST) (U/L)	11	31.00	25.04	47.00	49.00	26.50	100.30	0.0104
Total bilirubin (µmol/L)	11	9.55	0.69	13.68	13.00	0.64	19.50	0.0612
Lactate dehydrogenase (LDH) (U/L)	15	281.00	210.41	686.23	443.50	320.50	1684.00	0.0009

**Coagulation function**								
D-dimer (mg/L or μg/mL)	13	0.50	<0.01	4.10	1.76	0.36	11.97	0.0333
**Inflammatory markers**								
C-reactive protein (CRP) (mg/L)	16	39.32	8.00	68.88	103.22	9.90	177.00	0.0005
Procalcitonin (PCT) (×100 ng/mL)	9	13.00	5.00	41.00	44.00	10.00	270.00	0.0331
**Myocardial injury marker**								
Hypersensitive troponin I (hs-cTnI) (×100 ng/mL)	4	0.46	0.35	1.00	1.03	0.33	1.10	0.3836

* It was the number of studies without missing values among the collected 21 studies.

† The listed *P* values were calculated using the Wilcoxon rank-sum test based on the available number of studies.

Table S4. The summary statistics of the demographics, clinical characteristics, and biomarkers of the COVID-19 patients on initial hospital presentations from the collected 24 studies for the assessment of mortality

Variable	Number of Studies* (*n*)	Alive			Dead			*P* Value†
		Median	Minimum	Maximum	Median	Minimum	Maximum	
**Demographics**								
Age (years)	22	61.42	38.70	86.00	72.50	55.00	87.00	<0.0001
Male (%)	23	55.00	38.50	89.00	64.30	20.00	84.21	0.0588
Current smoker (%)	7	3.00	2.00	6.30	7.00	2.00	21.21	0.1098
**Vital signs**								
Heart rate (beats/min)	12	86.10	80.00	98.00	91.00	80.00	101.00	0.0990
Respiratory rate (breaths/min)	12	20.00	20.00	23.00	23.70	20.00	26.00	0.0020
Systolic pressure (mmHg)	9	125.20	110.70	138.10	130.50	114.00	140.50	0.0771
**Clinical features**								
Fever (%)	15	76.54	11.96	94.20	66.67	33.30	92.00	1
Cough (%)	16	63.34	27.90	81.48	63.18	38.00	84.21	0.8672
Fatigue (%)	8	40.50	3.30	76.54	52.50	10.50	88.90	0.3282
Myalgia (%)	12	16.01	5.90	61.50	12.45	2.10	55.60	0.4776
Dyspnea (%)	9	42.60	14.38	81.48	62.00	31.20	84.21	0.1331
Sputum production (%)	8	30.59	2.00	46.91	39.67	0.80	66.67	0.3282
Headache (%)	10	7.10	5.00	61.72	4.10	0	66.70	0.0892
Sore throat (%)	8	7.90	2.00	32.70	4.60	0	22.20	0.4005
Diarrhea (%)	14	14.11	1.30	31.00	10.86	0	29.80	0.4481

Shortness of breath (%)	5	43.20	2.00	63.04	65.80	42.10	75.00	0.0952
Chest congestion / Chest tightness (%)	2	22.19	14.38	30.00	40.83	32.65	49.00	0.3333
Chest pain (%)	6	11.10	5.00	38.50	7.15	2.60	63.15	0.4848
**Comorbidities**								
Any (%)	9	54.17	29.70	67.90	78.90	61.15	100	0.0003
Hypertension (%)	21	29.00	15.30	68.00	61.20	22.20	100	0.0001
Diabetes (%)	22	16.39	5.10	37.68	30.00	9.00	55.00	0.0007
Cardiovascular disease (%)	21	10.00	1.90	85.14	26.30	10.30	100	0.0002
Chronic obstructive pulmonary disease (%)	20	4.30	0.37	20.00	14.17	5.30	33.30	0.0001
Malignancy (%)	14	3.26	0.96	10.50	10.80	0	20.00	0.0179
Cerebrovascular disease (%)	8	4.72	0	14.00	10.56	0	25.00	0.0927
Chronic liver disease (%)	10	2.20	0.46	8.00	5.25	0	10.52	0.2261
Chronic renal disease (%)	17	3.80	0.74	35.00	15.00	0	39.00	0.0011
**Blood routine**								
White blood cell count ($\times10^9$/L)	18	5.62	4.32	8.14	8.08	4.94	15.50	<0.0001
Neutrophil count ($\times10^9$/L)	15	3.92	2.90	6.50	6.45	4.12	14.70	<0.0001
Lymphocyte count ($\times10^9$/L)	21	1.00	0.80	2.60	0.70	0.45	1.45	<0.0001
Neutrophil/Lymphocyte ratio (NLR)	7	3.58	2.82	5.31	10.34	6.60	14.96	0.0006
Platelet count ($\times10^9$/L)	18	204.00	173.61	238.00	182.25	90.50	219.30	0.0108
**Blood biochemistry**								
Alanine aminotransferase (ALT) (U/L)	17	30.00	20.00	49.70	32.00	13.00	68.00	0.2774
Aspartate aminotransferase (AST) (U/L)	15	38.57	24.00	68.00	47.00	31.00	80.30	0.0224
Total bilirubin (μmol/L)	11	9.06	0.68	13.00	11.70	0.38	24.90	0.2371
Lactate dehydrogenase (LDH) (U/L)	14	369.86	201.00	628.86	532.50	318.00	1116.50	0.0009

**Coagulation function**								
D-dimer (mg/L or μg/mL)	18	0.80	0.18	1.83	2.28	0.74	17.80	<0.0001
**Inflammatory markers**								
C-reactive protein (CRP) (mg/L)	22	42.45	2.60	120.00	116.50	10.00	189.00	<0.0001
Procalcitonin (PCT) (×100 ng/mL)	13	9.00	0.25	58.00	33.00	0.04	330.00	0.0292
**Myocardial injury marker**								
	12	1.00	0.30	13.34	4.48	2.52	27.90	0.0003

* It was the number of studies without missing values among the collected 24 studies.

† The listed *P* values were calculated using the Wilcoxon rank-sum test based on the available number of studies.

Figure S1. Forest plot of mean/median differences in white blood cell count (×10^9/L) between COVID-19 patients with severe or non-severe status

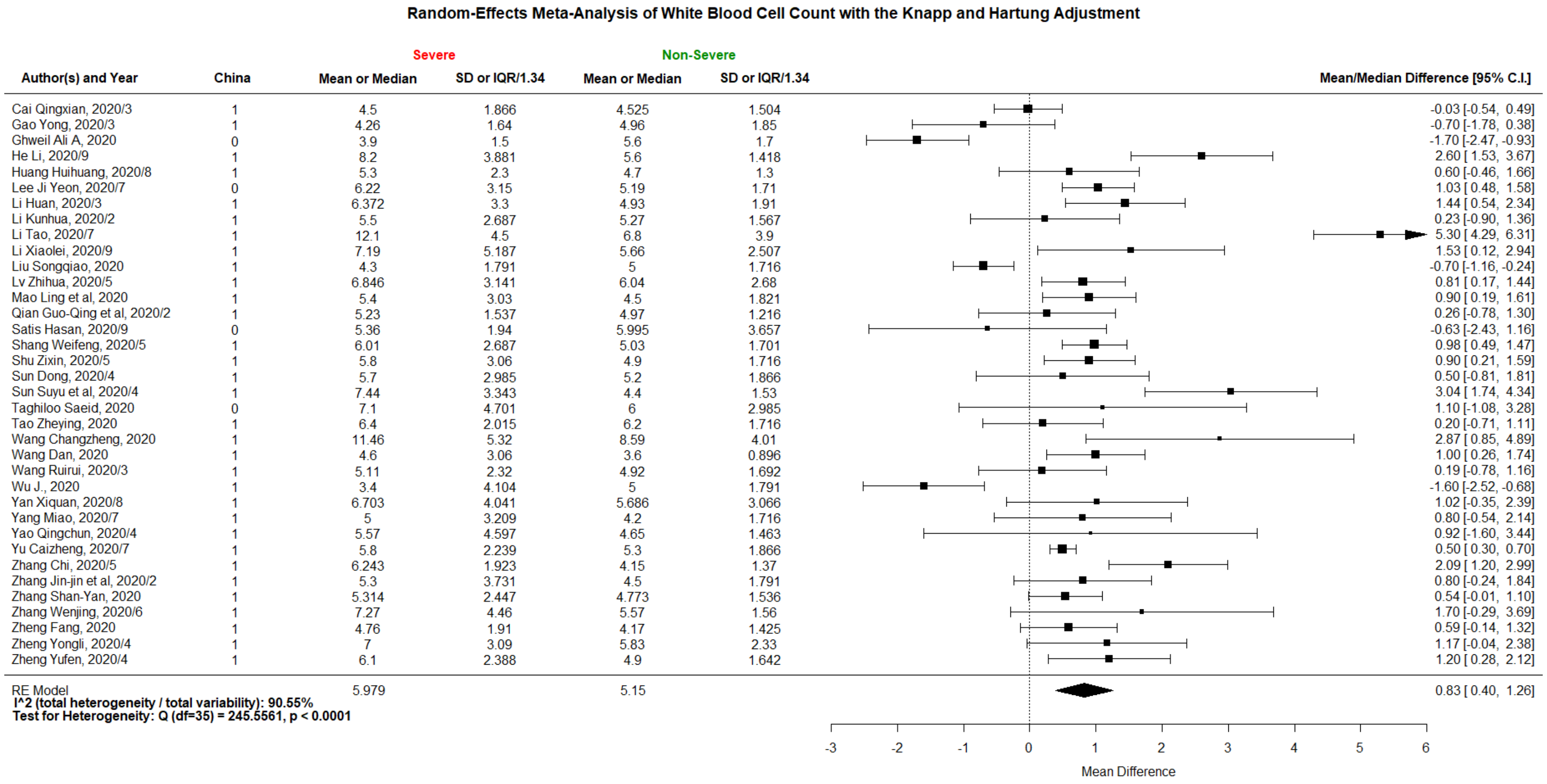

Figure S2. Forest plot of mean/median differences in neutrophil count (×10^9/L) between COVID-19 patients with severe or non-severe status

Random-Effects Meta-Analysis of Neutrophil Count with the Knapp and Hartung Adjustment

Author(s) and Year	China	Severe: Mean or Median	Severe: SD or IQR/1.34	Non-Severe: Mean or Median	Non-Severe: SD or IQR/1.34	Mean/Median Difference [95% C.I.]
Cai Qingxian, 2020/3	1	7.35	3.134	6.65	2.537	0.70 [-0.17, 1.57]
Gao Yong, 2020/3	1	1.65	1.49	3.43	1.63	-1.78 [-2.75, -0.81]
He Li, 2020/9	1	6.2	3.507	3	1.269	3.20 [2.24, 4.16]
Lee Ji Yeon, 2020/7	0	4.57	3	2.93	1.36	1.64 [1.13, 2.15]
Li Kunhua, 2020/2	1	4.36	2.694	3.5	1.358	0.86 [-0.25, 1.97]
Li Tao, 2020/7	1	10.3	5.1	5.7	3.5	4.60 [3.51, 5.69]
Li Xiaolei, 2020/9	1	6.4	4.83	3.8	1.985	2.60 [1.30, 3.90]
Liu Songqiao, 2020	1	2.9	1.791	3	1.343	-0.10 [-0.55, 0.35]
Lv Zhihua, 2020/5	1	5.257	3.089	4.15	2.67	1.11 [0.48, 1.73]
Mao Ling et al, 2020	1	3.8	2.791	2.6	1.657	1.20 [0.55, 1.85]
Qian Guo-Qing et al, 2020/2	1	3.32	2.104	2.8	0.978	0.52 [-0.87, 1.91]
Shang Weifeng, 2020/5	1	4.27	2.679	3.07	1.343	1.20 [0.73, 1.67]
Shu Zixin, 2020/5	1	4.6	2.612	3.4	1.269	1.20 [0.62, 1.78]
Sun Dong, 2020/4	1	3.4	2.164	3.5	1.493	-0.10 [-1.06, 0.86]
Sun Suyu et al, 2020/4	1	6.07	3.358	2.9	1.231	3.17 [1.88, 4.46]
Tao Zheying, 2020	1	5.2	2.687	3.6	1.343	1.60 [0.41, 2.79]
Wang Changzheng, 2020	1	10.51	5.1	6.69	3.83	3.82 [1.88, 5.76]
Wang Dan, 2020	1	4.5	3.134	3.2	1.716	1.30 [0.47, 2.13]
Wang Ruirui, 2020/3	1	3.86	2.18	3.49	1.62	0.37 [-0.54, 1.28]
Wu J., 2020	1	2.2	0.522	3.1	1.045	-0.90 [-1.08, -0.72]
Yang Miao, 2020/7	1	3.1	2.761	2.5	1.418	0.60 [-0.55, 1.75]
Yao Qingchun, 2020/4	1	3.33	2.299	2.53	1.41	0.80 [-0.49, 2.09]
Yu Caizheng, 2020/7	1	3.9	2.164	3.6	1.716	0.30 [0.11, 0.49]
Zhang Chi, 2020/5	1	5.08	2.444	2.4	1.25	2.68 [1.58, 3.78]
Zhang Shan-Yan, 2020	1	3.767	2.246	2.951	1.271	0.82 [0.31, 1.32]
Zhang Wenjing, 2020/6	1	3.88	5.381	3.24	1.276	0.64 [-1.74, 3.02]
Zheng Yongli, 2020/4	1	5.42	2.84	3.78	2.08	1.64 [0.54, 2.74]
Zheng Yufen, 2020/4	1	5.1	2.537	3.2	1.493	1.90 [0.94, 2.86]
RE Model		4.711		3.495		1.22 [0.69, 1.74]

I^2 (total heterogeneity / total variability): 94.68%
Test for Heterogeneity: Q (df= 27) = 437.5327, p < 0.0001

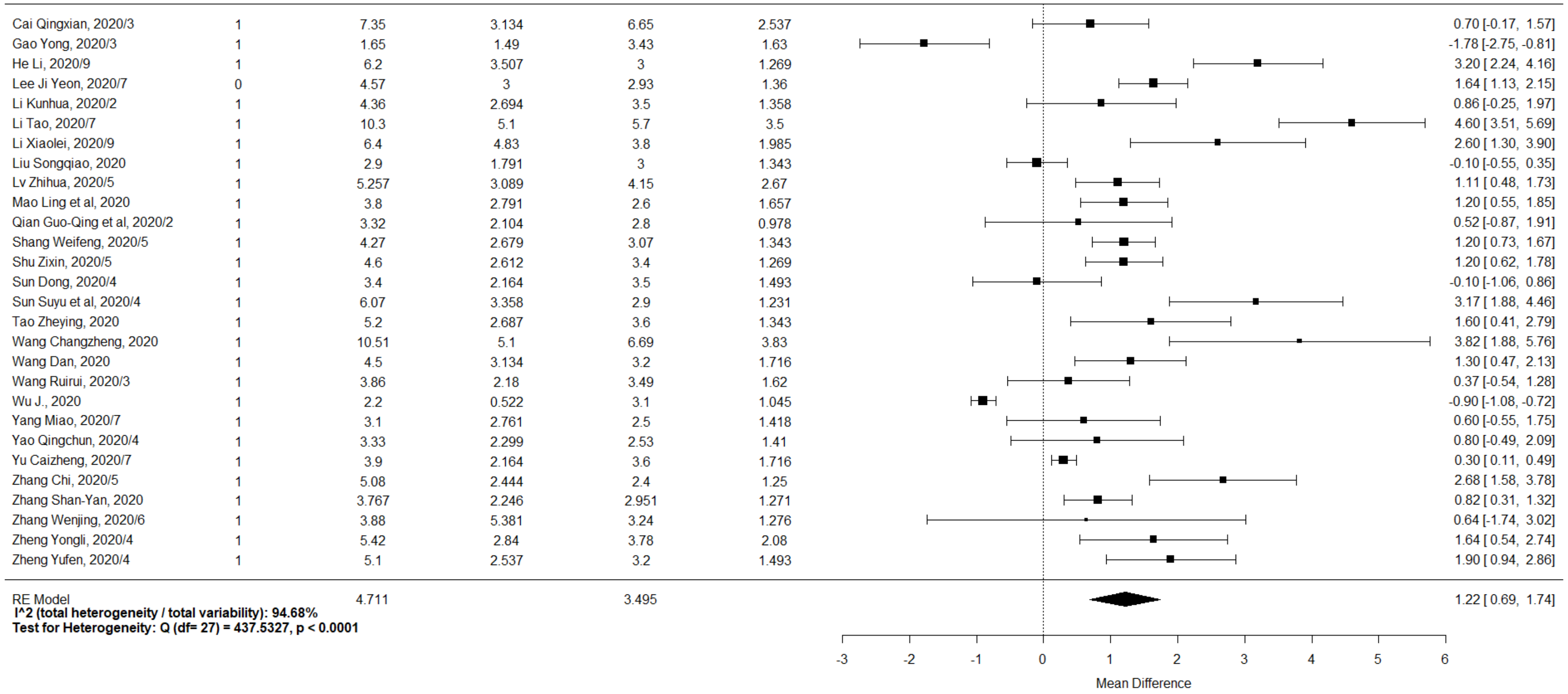

Figure S3. Forest plot of mean/median differences in neutrophil/lymphocyte ratio (NLR) between COVID-19 patients with severe or non-severe status

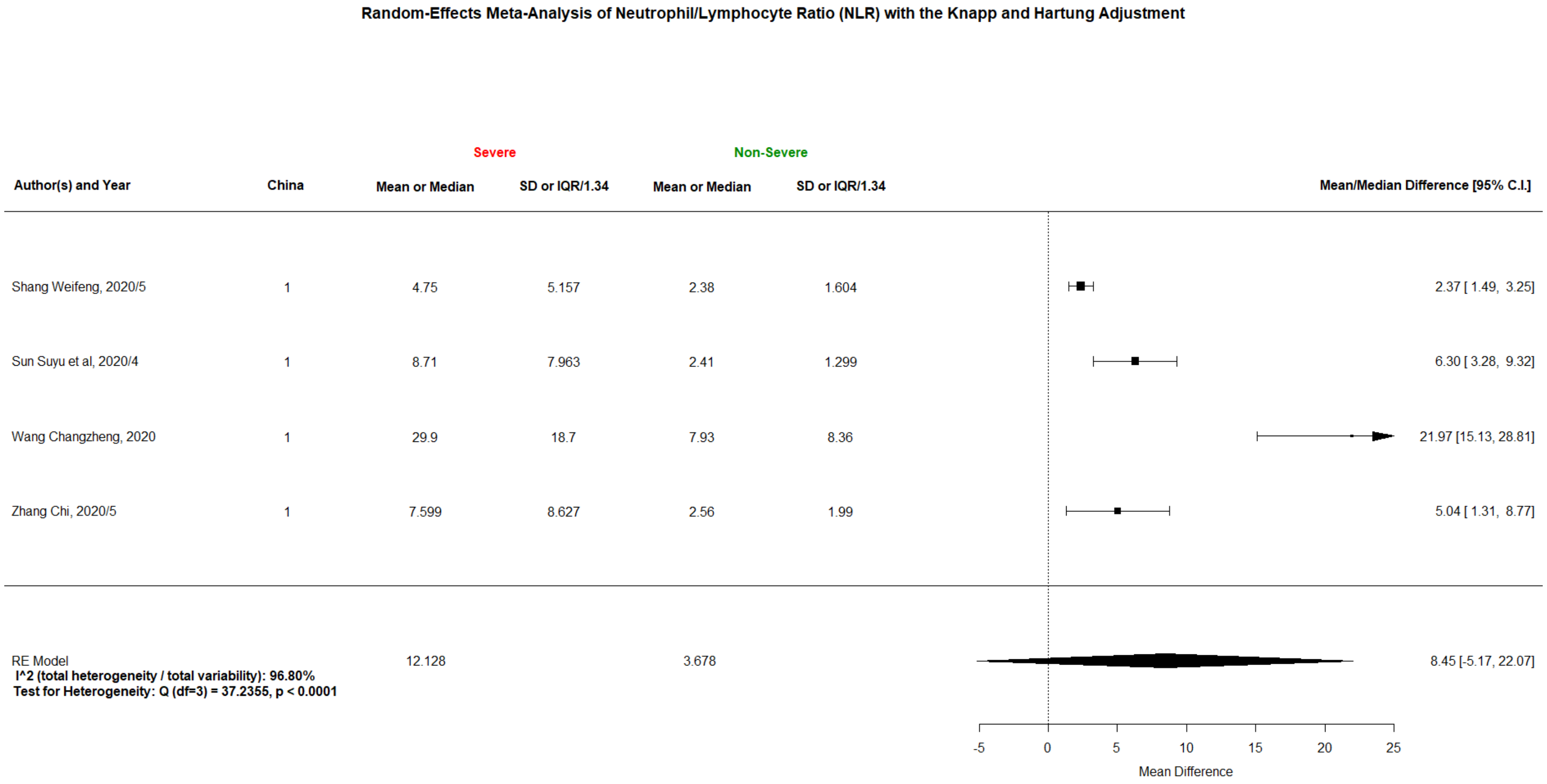

Figure S4. Forest plot of mean/median differences in platelet count (×10^9/L) between COVID-19 patients with severe or non-severe status

Random-Effects Meta-Analysis of Platelet count with the Knapp and Hartung Adjustment

		Severe		Non-Severe		
Author(s) and Year	China	Mean or Median	SD or IQR/1.34	Mean or Median	SD or IQR/1.34	Mean/Median Difference [95% C.I.]
He Li, 2020/9	1	217.5	114.403	236	64.179	-18.50 [-51.10, 14.10]
Huang Huihuang, 2020/8	1	194.1	61.9	180.5	62.7	13.60 [-18.84, 46.04]
Lee Ji Yeon, 2020/7	0	215.87	91.56	240.46	83.17	-24.59 [-41.41, -7.77]
Li Huan, 2020/3	1	199.809	75.861	214.05	91.34	-14.24 [-43.24, 14.76]
Li Tao, 2020/7	1	157.4	95.3	233.7	92.6	-76.30 [-98.47, -54.13]
Liu Songqiao, 2020	1	154	50	188.5	50.746	-34.50 [-47.45, -21.55]
Mao Ling et al, 2020	1	204.5	83.284	219	80.746	-14.50 [-36.90, 7.90]
Qian Guo-Qing et al, 2020/2	1	152	60.448	198	77.612	-46.00 [-88.92, -3.08]
Shang Weifeng, 2020/5	1	181	76.119	227.5	90.485	-46.50 [-62.74, -30.26]
Shu Zixin, 2020/5	1	190	74.627	228	71.642	-38.00 [-56.55, -19.45]
Sun Dong, 2020/4	1	265	143.284	176	76.343	89.00 [27.39, 150.61]
Sun Suyu et al, 2020/4	1	161	60.448	184	57.836	-23.00 [-48.77, 2.77]
Taghiloo Saeid, 2020	0	178	86.94	214.5	59.328	-36.50 [-77.32, 4.32]
Tao Zheying, 2020	1	193	72.985	229.5	69.254	-36.50 [-69.88, -3.12]
Wang Changzheng, 2020	1	210.6	60.43	222.7	73.01	-12.10 [-37.08, 12.88]
Wang Dan, 2020	1	154	83.582	156	74.627	-2.00 [-27.98, 23.98]
Wang Ruirui, 2020/3	1	163.4	60.263	169.5	52.239	-6.10 [-31.85, 19.65]
Wu J., 2020	1	86	15	196	59	-110.00 [-118.85, -101.15]
Yang Miao, 2020/7	1	174	82.463	181.8	63.806	-7.80 [-43.50, 27.90]
Yao Qingchun, 2020/4	1	145	61.194	195	67.91	-50.00 [-86.33, -13.67]
Yu Caizheng, 2020/7	1	237	104.478	213	96.269	24.00 [14.35, 33.65]
Zhang Chi, 2020/5	1	227.143	129.484	211.52	81.87	15.62 [-43.76, 75.01]
Zhang Shan-Yan, 2020	1	166.333	54.033	181.904	54.676	-15.57 [-28.22, -2.92]
Zhang Wenjing, 2020/6	1	165	39.552	209	74.627	-44.00 [-68.62, -19.38]
Zheng Fang, 2020	1	160	60.448	171	62.687	-11.00 [-35.15, 13.15]
Zheng Yufen, 2020/4	1	158	72.388	203	57.463	-45.00 [-73.41, -16.59]
RE Model		178.93		203.582		-24.65 [-38.45, -10.85]

I^2 (total heterogeneity / total variability): 89.71%
Test for Heterogeneity: Q (df=25) = 501.6439, p < 0.0001

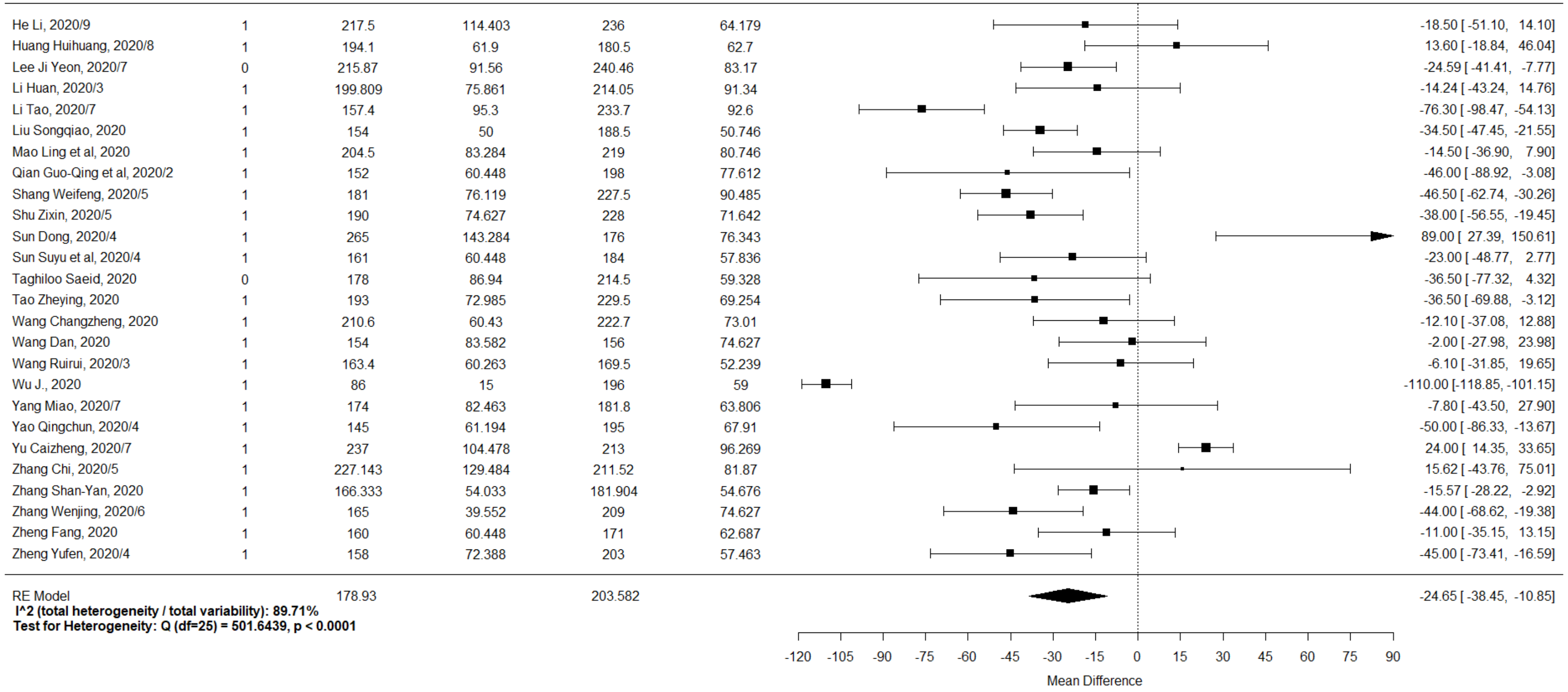

Figure S5. Forest plot of mean/median differences in alanine aminotransferase (ALT) (U/L) between COVID-19 patients with severe or non-severe status

Random-Effects Meta-Analysis of Alanine Aminotransferase (ALT) with the Knapp and Hartung Adjustment

		Severe		Non-Severe		
Author(s) and Year	China	Mean or Median	SD or IQR/1.34	Mean or Median	SD or IQR/1.34	Mean/Median Difference [95% C.I.]
Cai Qingxian, 2020/3	1	26.85	15.522	20	11.381	6.85 [2.60, 11.10]
Gao Yong, 2020/3	1	27	14.925	24.5	16.418	2.50 [-7.20, 12.20]
Ghweil Ali A, 2020	0	41.5	6.7	33.3	8.03	8.20 [4.65, 11.75]
He Li, 2020/9	1	28	25.224	19	15.522	9.00 [1.72, 16.28]
Huang Huihuang, 2020/8	1	31	23.134	22	16.418	9.00 [-2.04, 20.04]
Lee Ji Yeon, 2020/7	0	28.5	22.01	23.88	17.45	4.62 [0.66, 8.58]
Li Tao, 2020/7	1	48.2	20.1	31.6	18.9	16.60 [11.97, 21.23]
Mao Ling et al, 2020	1	32.5	287.761	23	38.06	9.50 [-50.99, 69.99]
Qian Guo-Qing et al, 2020/2	1	19.9	8.955	18	11.94	1.90 [-4.50, 8.30]
Satis Hasan, 2020/9	0	25	11.194	28.5	39.179	-3.50 [-19.69, 12.69]
Shu Zixin, 2020/5	1	33	20.149	20	7.463	13.00 [8.62, 17.38]
Taghiloo Saeid, 2020	0	54	29.104	33	11.791	21.00 [8.29, 33.71]
Tao Zheying, 2020	1	27	15.448	18	12.687	9.00 [2.01, 15.99]
Uchida Yoshihito, 2020/7	0	34	110.448	21	88.06	13.00 [-70.43, 96.43]
Wang Dan, 2020	1	50	35.821	35.8	21.194	14.20 [4.54, 23.86]
Wang Ruirui, 2020/3	1	25	14.552	24	18.47	1.00 [-5.76, 7.76]
Wu J., 2020	1	24	14.925	20	16.418	4.00 [0.05, 7.95]
Yan Xiquan, 2020/8	1	32.427	26.362	27.067	19.311	5.36 [-3.55, 14.27]
Yang Miao, 2020/7	1	33	24.627	21	10.448	12.00 [1.90, 22.10]
Yao Qingchun, 2020/4	1	23	7.239	20	11.94	3.00 [-1.70, 7.70]
Yu Caizheng, 2020/7	1	24	16.418	22	17.164	2.00 [0.38, 3.62]
Zhang Chi, 2020/5	1	36.051	20.825	40.53	41.19	-4.48 [-18.47, 9.51]
Zhang Shan-Yan, 2020	1	28.545	13.238	24.634	10.017	3.91 [0.88, 6.94]
Zheng Fang, 2020	1	23.9	13.209	19.3	2.388	4.60 [-0.14, 9.34]
Zheng Yongli, 2020/4	1	42.37	48.88	31.68	27.75	10.69 [-7.50, 28.88]
RE Model		30.489		23.926		6.56 [4.41, 8.71]

I^2 (total heterogeneity / total variability): 68.32%
Test for Heterogeneity: Q (df=24) = 75.6209, p < 0.0001

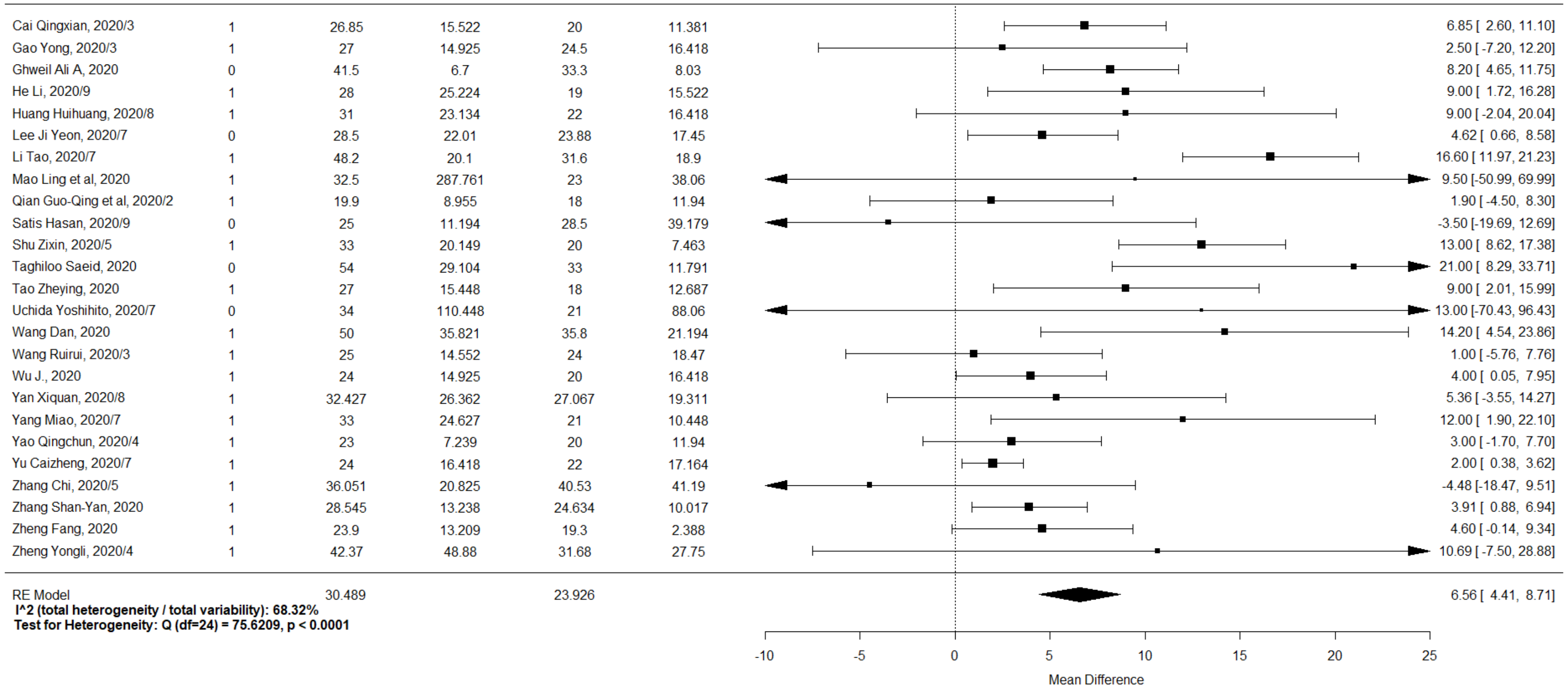

Figure S6. Forest plot of mean/median differences in aspartate aminotransferase (AST) (U/L) between COVID-19 patients with severe or non-severe status

Random-Effects Meta-Analysis of Aspartate Aminotransferase (AST) with the Knapp and Hartung Adjustment

		Severe		Non-Severe		
**Author(s) and Year**	**China**	**Mean or Median**	**SD or IQR/1.34**	**Mean or Median**	**SD or IQR/1.34**	**Mean/Median Difference [95% C.I.]**
Cai Qingxian, 2020/3	1	36	15.149	26	9.701	10.00 [5.91, 14.09]
Gao Yong, 2020/3	1	27.8	11.42	33.21	18.24	-5.41 [-14.30, 3.48]
Ghweil Ali A, 2020	0	44.5	4.5	32.8	7.2	11.70 [8.85, 14.55]
He Li, 2020/9	1	26	20.373	18.5	9.328	7.50 [1.82, 13.18]
Huang Huihuang, 2020/8	1	29	28.358	24	6.716	5.00 [-7.29, 17.29]
Lee Ji Yeon, 2020/7	0	36.63	26.6	23.87	11.62	12.76 [8.20, 17.32]
Li Tao, 2020/7	1	50.3	29.8	40.6	22.7	9.70 [3.22, 16.18]
Mao Ling et al, 2020	1	34	1221.343	23	35.075	11.00 [-244.25, 266.25]
Qian Guo-Qing et al, 2020/2	1	27	2.425	21	8.955	6.00 [3.50, 8.50]
Satis Hasan, 2020/9	0	35	22.388	24	8.582	11.00 [-2.62, 24.62]
Taghiloo Saeid, 2020	0	59.5	29.478	41.5	15.149	18.00 [4.80, 31.20]
Tao Zheying, 2020	1	31.5	22.761	22	7.463	9.50 [-0.53, 19.53]
Uchida Yoshihito, 2020/7	0	40	76.119	28	66.418	12.00 [-46.39, 70.39]
Wang Dan, 2020	1	61	32.463	42.6	19.776	18.40 [9.57, 27.23]
Wang Ruirui, 2020/3	1	29	9.701	26	10.448	3.00 [-1.32, 7.32]
Wu J., 2020	1	26	11.94	16	9.701	10.00 [7.10, 12.90]
Yan Xiquan, 2020/8	1	33.844	15.417	27.077	12.691	6.77 [1.48, 12.06]
Yang Miao, 2020/7	1	39.5	28.358	24	8.955	15.50 [3.99, 27.01]
Yu Caizheng, 2020/7	1	27	14.179	25	12.687	2.00 [0.71, 3.29]
Zhang Chi, 2020/5	1	40.954	22.231	37.67	33.71	3.28 [-9.69, 16.26]
Zhang Shan-Yan, 2020	1	28.545	13.238	24.78	10.017	3.76 [0.74, 6.79]
Zheng Fang, 2020	1	31.6	17.507	23.4	7.313	8.20 [1.81, 14.59]
Zheng Yongli, 2020/4	1	51.23	88.67	26.53	12.73	24.70 [-6.17, 55.57]
RE Model		34.329		26.459		7.87 [5.65, 10.09]

I^2 (total heterogeneity / total variability): 73.93%
Test for Heterogeneity: Q (df=22) = 98.9182, p < 0.0001

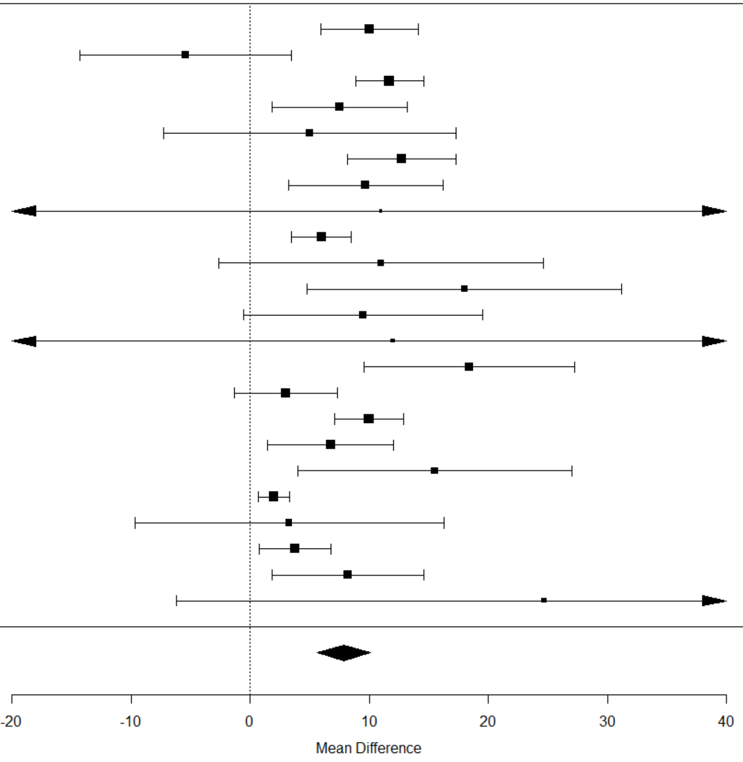

Figure S7. Forest plot of mean/median differences in total bilirubin (μmol/L) between COVID-19 patients with severe or non-severe status

Random-Effects Meta-Analysis of Total Bilirubin with the Knapp and Hartung Adjustment

Author(s) and Year	China	Severe: Mean or Median	Severe: SD or IQR/1.34	Non-Severe: Mean or Median	Non-Severe: SD or IQR/1.34	Mean/Median Difference [95% C.I.]
Cai Qingxian, 2020/3	1	11.25	7.388	10.9	5.597	0.35 [-1.68, 2.38]
Huang Huihuang, 2020/8	1	11.3	4.03	10	5.448	1.30 [-1.07, 3.67]
Li Tao, 2020/7	1	21.2	9.6	13.7	9.9	7.50 [5.22, 9.78]
Shu Zixin, 2020/5	1	10.3	4.776	7.2	2.91	3.10 [2.02, 4.18]
Taghiloo Saeid, 2020	0	20.52	8.933	13.68	5.104	6.84 [2.78, 10.90]
Tao Zheying, 2020	1	14.5	6.045	9.8	3.657	4.70 [2.00, 7.40]
Uchida Yoshihito, 2020/7	0	10.26	20.418	6.84	14.037	3.42 [-11.69, 18.53]
Wang Dan, 2020	1	16.2	6.269	13.5	6.269	2.70 [0.65, 4.75]
Wang Ruirui, 2020/3	1	10.4	8.134	9.25	5.616	1.15 [-2.22, 4.52]
Wu J., 2020	1	6.7	5.299	6.6	5.149	0.10 [-1.25, 1.45]
Yang Miao, 2020/7	1	11	6.194	9.7	3.806	1.30 [-1.31, 3.91]
Yao Qingchun, 2020/4	1	12.7	4.925	9.7	3.955	3.00 [0.19, 5.81]
Zhang Chi, 2020/5	1	12.509	6.842	9.82	4.19	2.69 [-0.44, 5.81]
Zhang Shan-Yan, 2020	1	10.613	5.626	9.449	4.514	1.16 [-0.13, 2.46]
Zheng Fang, 2020	1	12.7	5.746	10.7	5.313	2.00 [-0.25, 4.25]
RE Model		12.617		10.08		2.54 [1.34, 3.73]

I^2 (total heterogeneity / total variability): 73.82%
Test for Heterogeneity: Q (df=14) = 48.8090, p < 0.0001

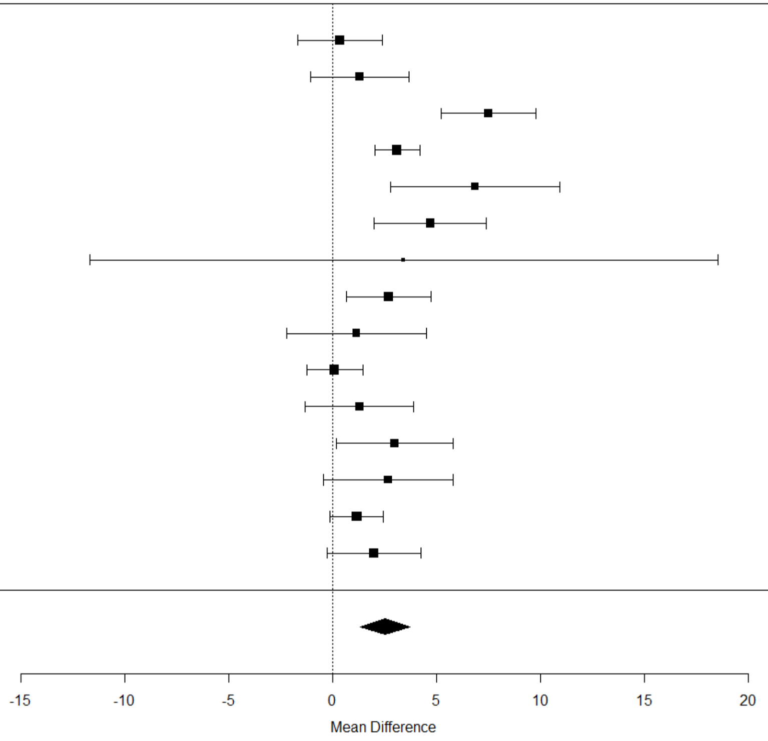

Figure S8. Forest plot of mean/median differences in lactate dehydrogenase (LDH) (U/L) between COVID-19 patients with severe or non-severe status

Random-Effects Meta-Analysis of Lactate Dehydrogenase with the Knapp and Hartung Adjustment

Author(s) and Year	China	Severe: Mean or Median	Severe: SD or IQR/1.34	Non-Severe: Mean or Median	Non-Severe: SD or IQR/1.34	Mean/Median Difference [95% C.I.]
Cai Qingxian, 2020/3	1	387	275.373	216	133.582	171.00 [98.14, 243.86]
He Li, 2020/9	1	287	150.522	179	34.701	108.00 [67.38, 148.62]
Huang Huihuang, 2020/8	1	356.8	204.6	209.2	52.2	147.60 [58.71, 236.49]
Lee Ji Yeon, 2020/7	0	695.19	455.13	447.55	124.11	247.64 [170.73, 324.55]
Li Tao, 2020/7	1	220.1	42.1	150.4	58.4	69.70 [58.38, 81.02]
Mao Ling et al, 2020	1	302	131.015	215	135.149	87.00 [50.86, 123.14]
Satis Hasan, 2020/9	0	414	232.09	245	73.881	169.00 [29.04, 308.96]
Shang Weifeng, 2020/5	1	277	181.343	206	61.567	71.00 [40.07, 101.93]
Taghiloo Saeid, 2020	0	631	146.269	489	155.97	142.00 [63.69, 220.31]
Tao Zheying, 2020	1	261	142.91	173	44.03	88.00 [25.07, 150.93]
Wang Dan, 2020	1	321	160.448	214	77.612	107.00 [65.60, 148.40]
Wu J., 2020	1	235	138.06	184	79.851	51.00 [19.27, 82.73]
Yan Xiquan, 2020/8	1	372.906	324.305	209.547	74.348	163.36 [59.59, 267.13]
Yu Caizheng, 2020/7	1	282	108.209	263	86.567	19.00 [9.61, 28.39]
Zhang Shan-Yan, 2020	1	282.571	100.922	205.12	64.616	77.45 [54.56, 100.35]
Zhang Wenjing, 2020/6	1	220.5	131.716	160	49.254	60.50 [1.63, 119.37]
Zheng Fang, 2020	1	226.2	90.746	162	55.821	64.20 [30.35, 98.05]
RE Model		317.382		221.591		95.79 [68.22, 123.36]

I^2 (total heterogeneity / total variability): 90.13%
Test for Heterogeneity: Q (df=16) = 134.0546, p < 0.0001

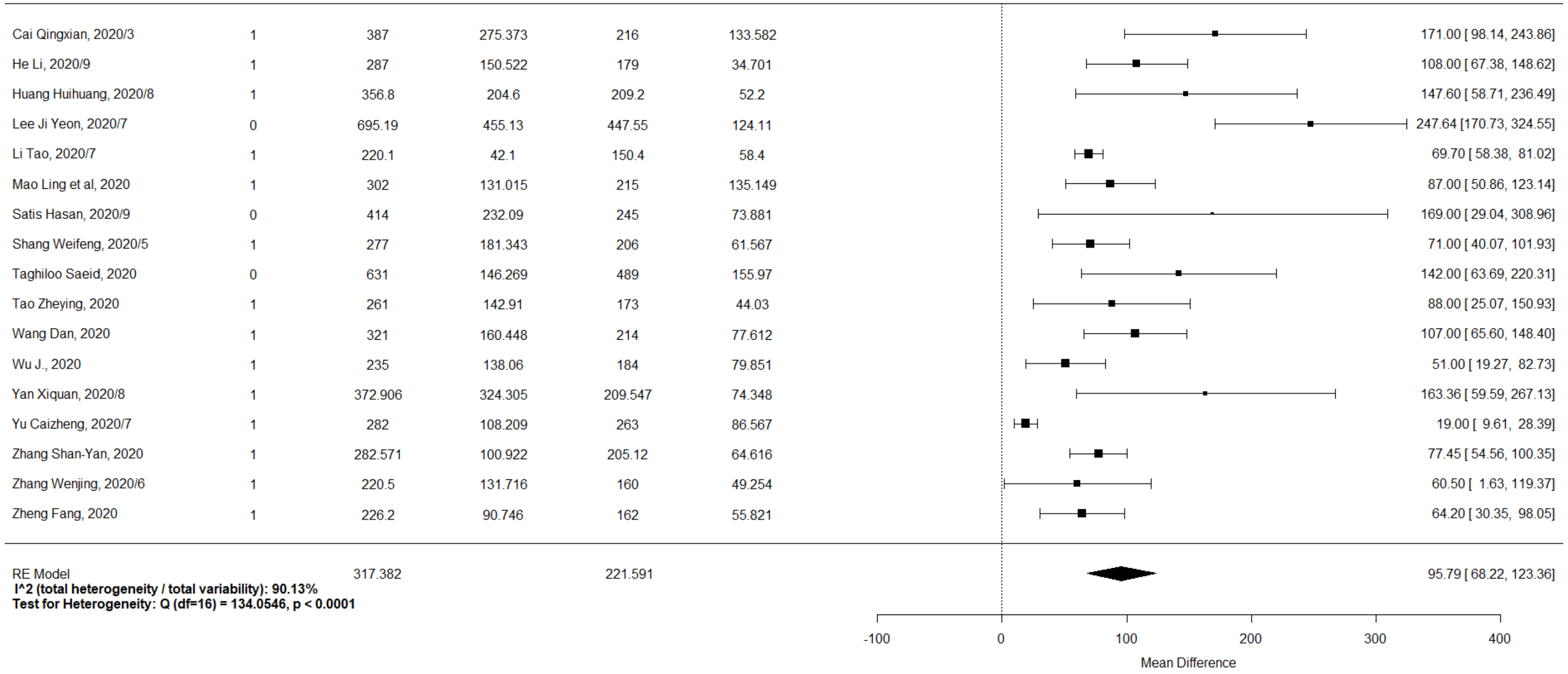

Figure S9. Forest plot of mean/median differences in D-dimer (mg/L or µg/mL) between COVID-19 patients with severe or non-severe status

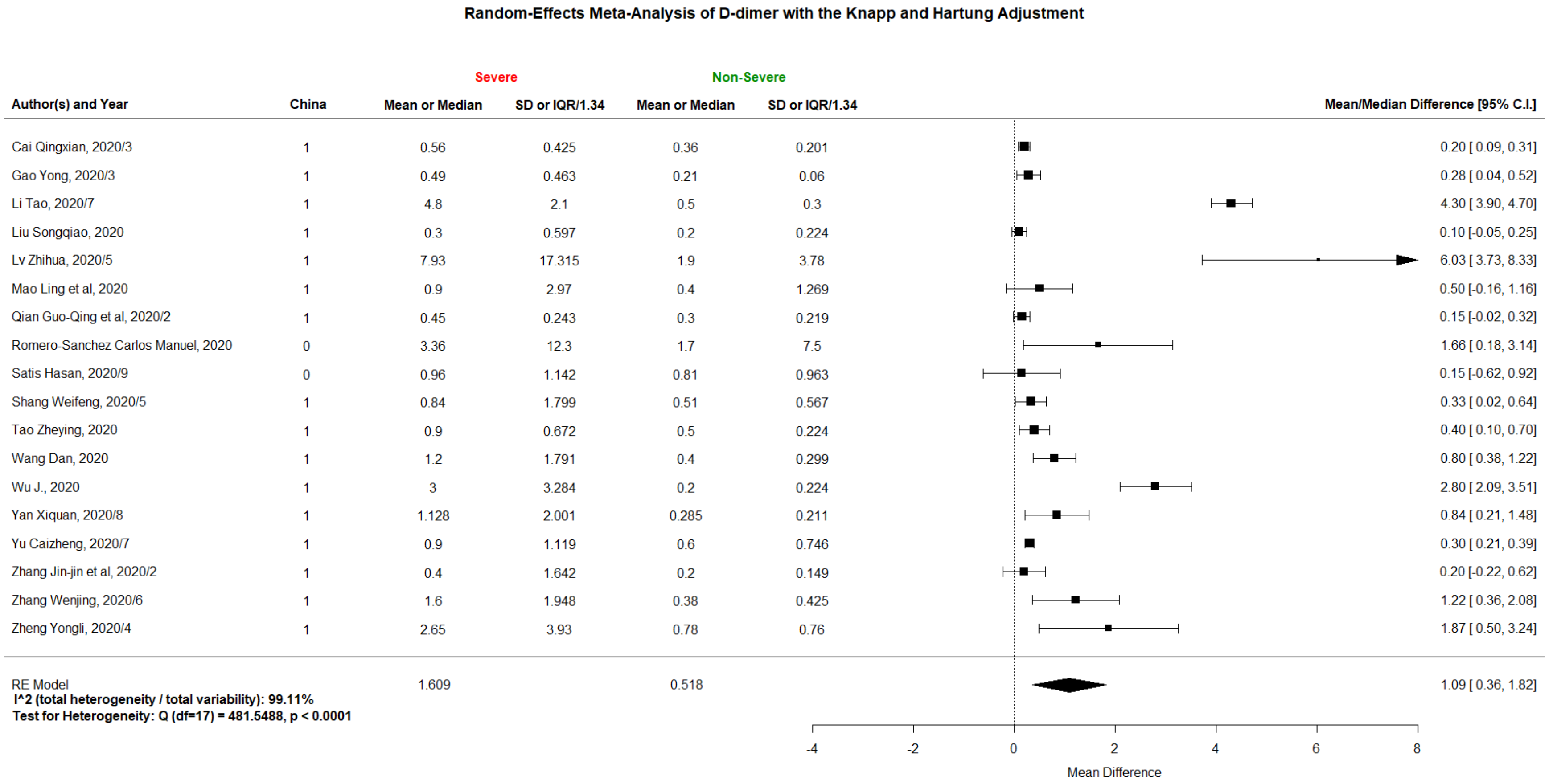

Figure S10. Forest plot of mean/median differences in C-reactive protein (CRP) (mg/L) between COVID-19 patients with severe or non-severe status

Random-Effects Meta-Analysis of C-Reactive Protein (CRP) with the Knapp and Hartung Adjustment

		Severe		Non-Severe		
Author(s) and Year	China	Mean or Median	SD or IQR/1.34	Mean or Median	SD or IQR/1.34	Mean/Median Difference [95% C.I.]
Cai Qingxian, 2020/3	1	32.235	29.687	8.18	13.597	24.05 [16.22, 31.89]
Gao Yong, 2020/3	1	39.37	27.68	18.76	22.2	20.61 [4.37, 36.85]
Ghweil Ali A, 2020	0	119.8	39	51.1	9.5	68.70 [54.40, 83.00]
He Li, 2020/9	1	29.1	48.507	0.7	1.94	28.40 [15.46, 41.34]
Huang Huihuang, 2020/8	1	19.5	23.657	6.7	6.642	12.80 [2.49, 23.11]
Lee Ji Yeon, 2020/7	0	66.6	66.9	8.6	18.5	58.00 [46.69, 69.31]
Li Huan, 2020/3	1	73.018	48.158	33.22	32.21	39.80 [26.01, 53.59]
Li Kunhua, 2020/2	1	89.2	64.746	9.59	20.761	79.61 [53.67, 105.55]
Lv Zhihua, 2020/5	1	53.971	52.903	33.22	45.08	20.75 [10.13, 31.38]
Mao Ling et al, 2020	1	37.1	31.627	9.4	18.746	27.70 [20.33, 35.07]
Qian Guo-Qing et al, 2020/2	1	30.63	67.836	5.98	7.388	24.65 [-19.70, 69.00]
Romero-Sanchez Carlos Manuel, 2020	0	189	269.05	106.66	119.26	82.34 [51.49, 113.19]
Satis Hasan, 2020/9	0	93	93.284	9.7	7.836	83.30 [28.09, 138.51]
Shang Weifeng, 2020/5	1	43.15	65.291	10.05	18.052	33.10 [22.06, 44.14]
Shu Zixin, 2020/5	1	58.3	71.269	8.1	17.239	50.20 [34.96, 65.44]
Sun Dong, 2020/4	1	83.9	71.866	12.5	19.776	71.40 [41.61, 101.19]
Tao Zheying, 2020	1	46.8	54.627	1.8	1.119	45.00 [21.06, 68.94]
Wang Dan, 2020	1	54.8	66.418	8.6	17.836	46.20 [30.21, 62.19]
Wang Ruirui, 2020/3	1	56.26	47.88	10.8	16.735	45.46 [26.41, 64.51]
Wu J., 2020	1	21.3	16.642	6.9	6.269	14.40 [10.71, 18.09]
Yan Xiquan, 2020/8	1	48.762	52.565	12.658	22.115	36.10 [19.03, 53.17]
Yang Miao, 2020/7	1	53.8	31.567	15.4	25.299	38.40 [24.66, 52.14]
Yao Qingchun, 2020/4	1	25.9	14.03	6.51	16.5	19.39 [10.98, 27.80]
Yu Caizheng, 2020/7	1	25.4	50.672	21	37.239	4.40 [0.15, 8.65]
Zhang Chi, 2020/5	1	54.489	54.123	20.3	24.99	34.19 [10.13, 58.24]
Zhang Jin-jin et al, 2020/2	1	47.6	49.627	28.7	31.791	18.90 [4.39, 33.41]
Zhang Shan-Yan, 2020	1	30.406	35.171	7.275	11.962	23.13 [15.28, 30.99]
Zhang Wenjing, 2020/6	1	62.8	59.701	1.7	6.642	61.10 [34.89, 87.31]
Zheng Fang, 2020	1	52.2	34.552	15.4	14.254	36.80 [24.20, 49.40]
RE Model		52.914		16.307		36.61 [28.87, 44.35]

I^2 (total heterogeneity / total variability): 91.54%
Test for Heterogeneity: Q (df=28) = 282.7721, p < 0.0001

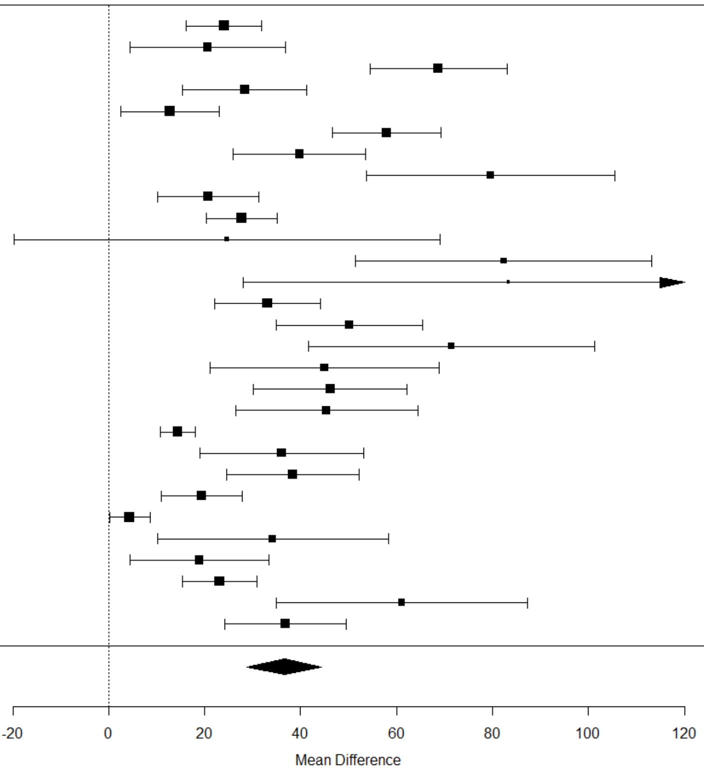

Figure S11. Forest plot of mean/median differences in procalcitonin (PCT) (×100 ng/mL) between COVID-19 patients with severe or non-severe status

Random-Effects Meta-Analysis of Procalcitonin (PCT) with the Knapp and Hartung Adjustment

		Severe		Non-Severe		
Author(s) and Year	China	Mean or Median	SD or IQR/1.34	Mean or Median	SD or IQR/1.34	Mean/Median Difference [95% C.I.]
Cai Qingxian, 2020/3	1	0.16	0.037	0.2	0.06	-0.04 [-0.05, -0.03]
Gao Yong, 2020/3	1	0.04	0.052	0.02	0.022	0.02 [-0.01, 0.05]
He Li, 2020/9	1	0.18	0.284	0.03	0.015	0.15 [0.07, 0.23]
Huang Huihuang, 2020/8	1	0.085	0.128	0.043	0.019	0.04 [-0.01, 0.10]
Lee Ji Yeon, 2020/7	0	0.033	0.034	0.016	0.022	0.02 [0.01, 0.02]
Li Huan, 2020/3	1	0.207	0.393	0.08	0.279	0.13 [0.01, 0.24]
Li Kunhua, 2020/2	1	0.086	0.12	0.038	0.03	0.05 [0.00, 0.10]
Li Tao, 2020/7	1	0.33	0.2	0.16	0.11	0.17 [0.13, 0.21]
Lv Zhihua, 2020/5	1	0.265	0.808	0.11	0.33	0.16 [0.04, 0.27]
Qian Guo-Qing et al, 2020/2	1	0	0	0.03	0.03	-0.03 [-0.04, -0.02]
Satis Hasan, 2020/9	0	0.19	0.183	0.06	0.03	0.13 [0.02, 0.24]
Shang Weifeng, 2020/5	1	0.07	0.149	0.03	0.022	0.04 [0.02, 0.06]
Sun Dong, 2020/4	1	0.08	0.056	0.04	0.028	0.04 [0.02, 0.06]
Tao Zheying, 2020	1	0.06	0.328	0.02	0.037	0.04 [-0.10, 0.18]
Wang Ruirui, 2020/3	1	0.05	0.087	0.026	0.031	0.02 [-0.01, 0.06]
Wu J., 2020	1	0.002	0.002	0.001	0.002	0.00 [-0.00, 0.00]
Yao Qingchun, 2020/4	1	0.19	0.224	0.2	0.179	-0.01 [-0.14, 0.12]
Yu Caizheng, 2020/7	1	0.06	0.045	0.05	0.037	0.01 [0.01, 0.01]
Zhang Chi, 2020/5	1	0.121	0.075	0.12	0.04	0.00 [-0.03, 0.04]
Zhang Jin-jin et al, 2020/2	1	0.1	0.179	0.05	0.052	0.05 [0.00, 0.10]
Zhang Shan-Yan, 2020	1	0.1	0.091	0.1	0.075	0.00 [-0.02, 0.02]
Zhang Wenjing, 2020/6	1	0.17	0.276	0.04	0.015	0.13 [0.01, 0.25]
RE Model		0.103		0.064		0.04 [0.01, 0.07]

I^2 (total heterogeneity / total variability): 99.21%
Test for Heterogeneity: Q (df=21) = 315.1955, p < 0.0001

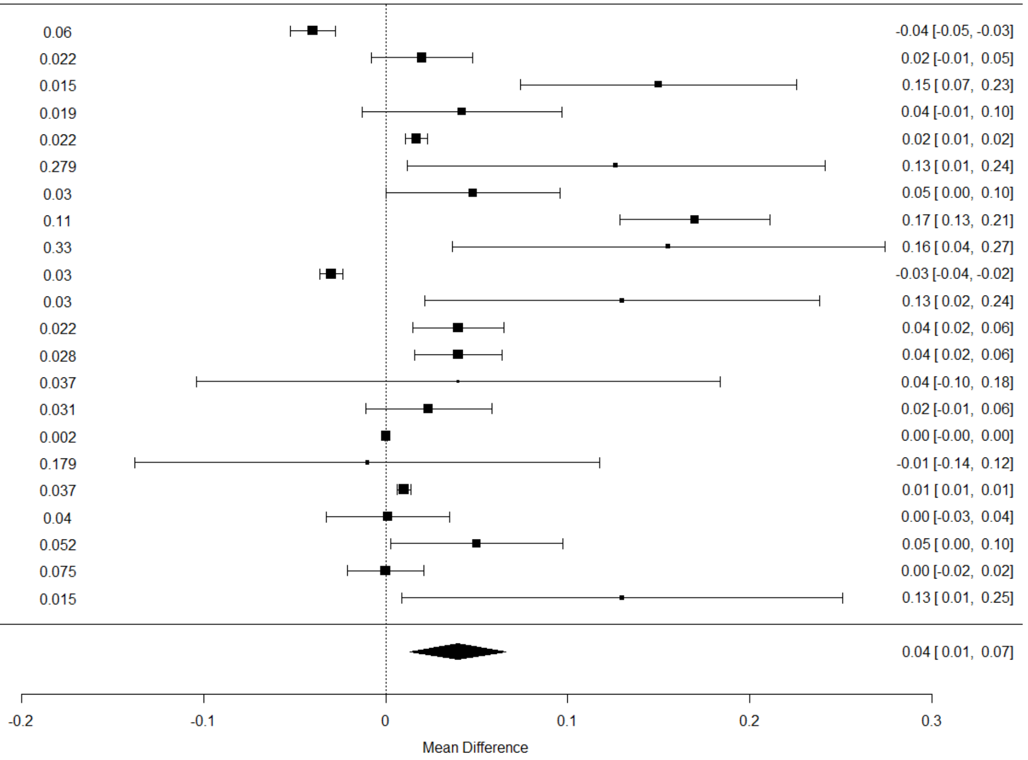

Figure S12. Forest plot of mean/median differences in hypersensitive troponin I (hs-cTnI) (×100 ng/mL) between COVID-19 patients with severe or non-severe status

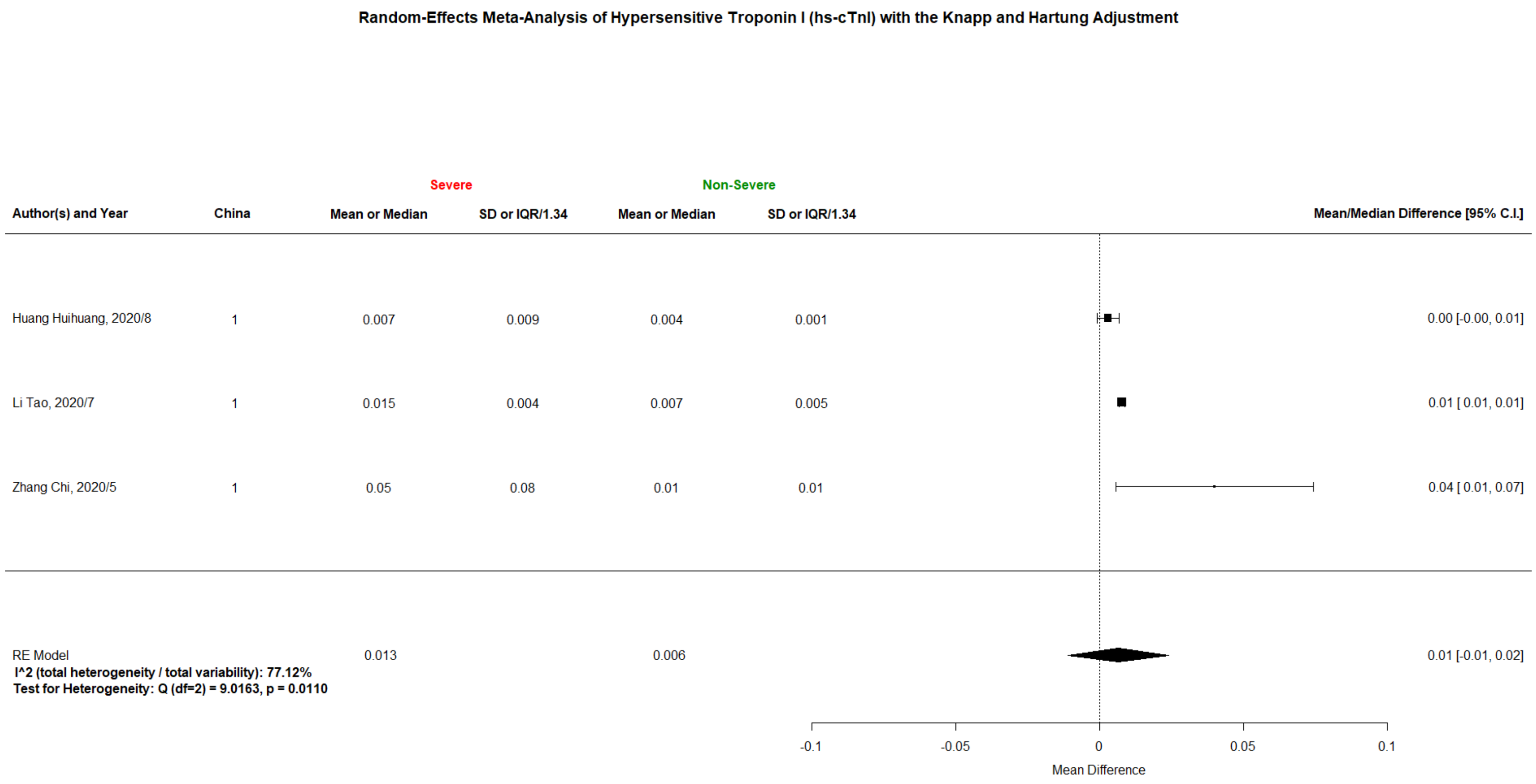

Figure S13. Forest plot of mean/median differences in white blood cell count ($\times 10^9$/L) between COVID-19 patients with critically severe or non-critically severe status

Random-Effects Meta-Analysis of White Blood Cell Count with the Knapp and Hartung Adjustment

Author(s) and Year	China	Critically Severe Mean or Median	Critically Severe SD or IQR/1.34	Non-Critically Severe Mean or Median	Non-Critically Severe SD or IQR/1.34	Mean/Median Difference [95% C.I.]
Almazeedi Sulaiman, 2020/7	0	6.61	2.7	6.1	2.1	0.51 [-0.32, 1.34]
Fan Bingwen Eugene. et al, 2020/3	0	5.1	3.507	4.7	1.343	0.40 [-1.92, 2.72]
Ferguson Jessica, 2020/8	0	5.2	2.239	5.7	2.761	-0.50 [-1.72, 0.72]
Hong Kyung Soo, 2020/4	0	8.9	3.3	6.1	3.2	2.80 [0.88, 4.72]
Huang Chaolin et al, 2020/2	1	11.3	4.701	5.7	3.358	5.60 [2.76, 8.44]
Jiang Hao, 2020/7	1	5.2	2.164	5.8	1.791	-0.60 [-1.71, 0.51]
Khamis Faryal, 2020/6	0	7.25	3.358	5	2.649	2.25 [0.67, 3.83]
Lendorf Maria Elisabeth, 2020	0	6.5	4.254	5.9	2.164	0.60 [-1.32, 2.52]
Li Huan, 2020/3	1	8.34	4.74	5.355	2.37	2.99 [0.62, 5.35]
Lv Zhihua, 2020/5	1	7.17	3.4	6.402	2.864	0.77 [-0.03, 1.57]
Ortiz-Brizuela Edgar, 2020/5	0	8.1	4.254	6	2.575	2.10 [0.48, 3.72]
Qun Sen, 2020/9	1	6.72	7.701	5.332	1.694	1.39 [-5.37, 8.14]
Shahriarirad Reza, 2020	0	6.62	2.68	6	2.49	0.62 [-1.04, 2.28]
Sun Ying, 2020/4	1	6.84	3.57	4.845	1.334	2.00 [-0.36, 4.35]
Wang Dawei et al, 2020/2	1	6.6	4.627	4.3	1.567	2.30 [0.76, 3.84]
Wang Feng, 2020/4	1	8.7	4.1	5.88	3.032	2.82 [0.58, 5.06]
Yan Xiquan, 2020/8	1	7.24	4.3	5.78	3.176	1.46 [-0.84, 3.76]
Yang Luhuan, 2020/5	1	5.76	2.806	4.37	1.5	1.39 [0.34, 2.44]
Zhang Shan-Yan, 2020	1	6.8	3.731	4.783	1.58	2.02 [0.24, 3.79]
Zhao Zirun, 2020/7	0	7.52	3.239	6.77	2.694	0.75 [0.22, 1.28]
RE Model		6.943		5.623		1.32 [0.72, 1.92]

I^2 (total heterogeneity / total variability): 65.03%
Test for Heterogeneity: Q (df=19) = 45.1340, p = 0.0007

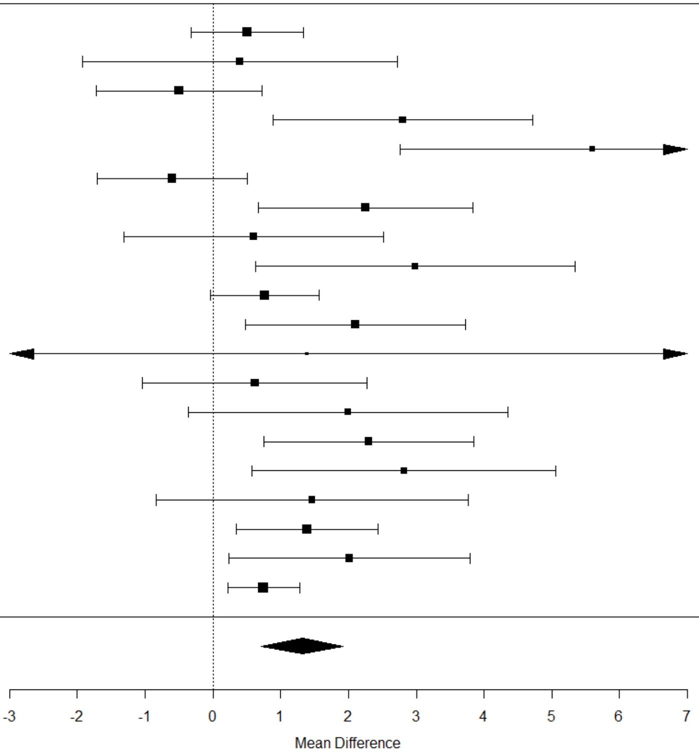

Figure S14. Forest plot of mean/median differences in neutrophil count ($\times 10^9$/L) between COVID-19 patients with critically severe or non-critically severe status

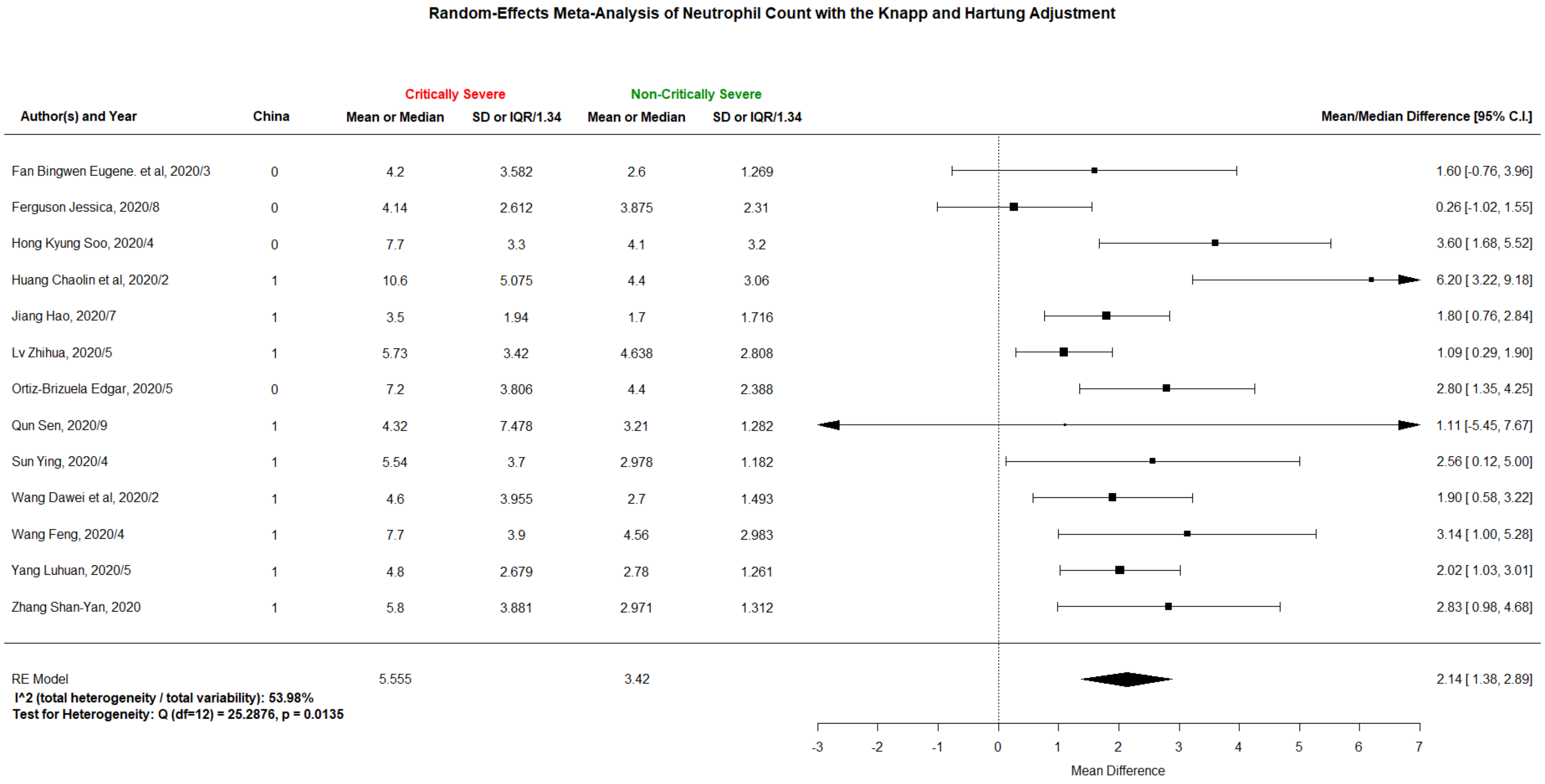

Figure S15. Forest plot of mean/median differences in neutrophil/lymphocyte ratio (NLR) between COVID-19 patients with critically severe or non-critically severe status

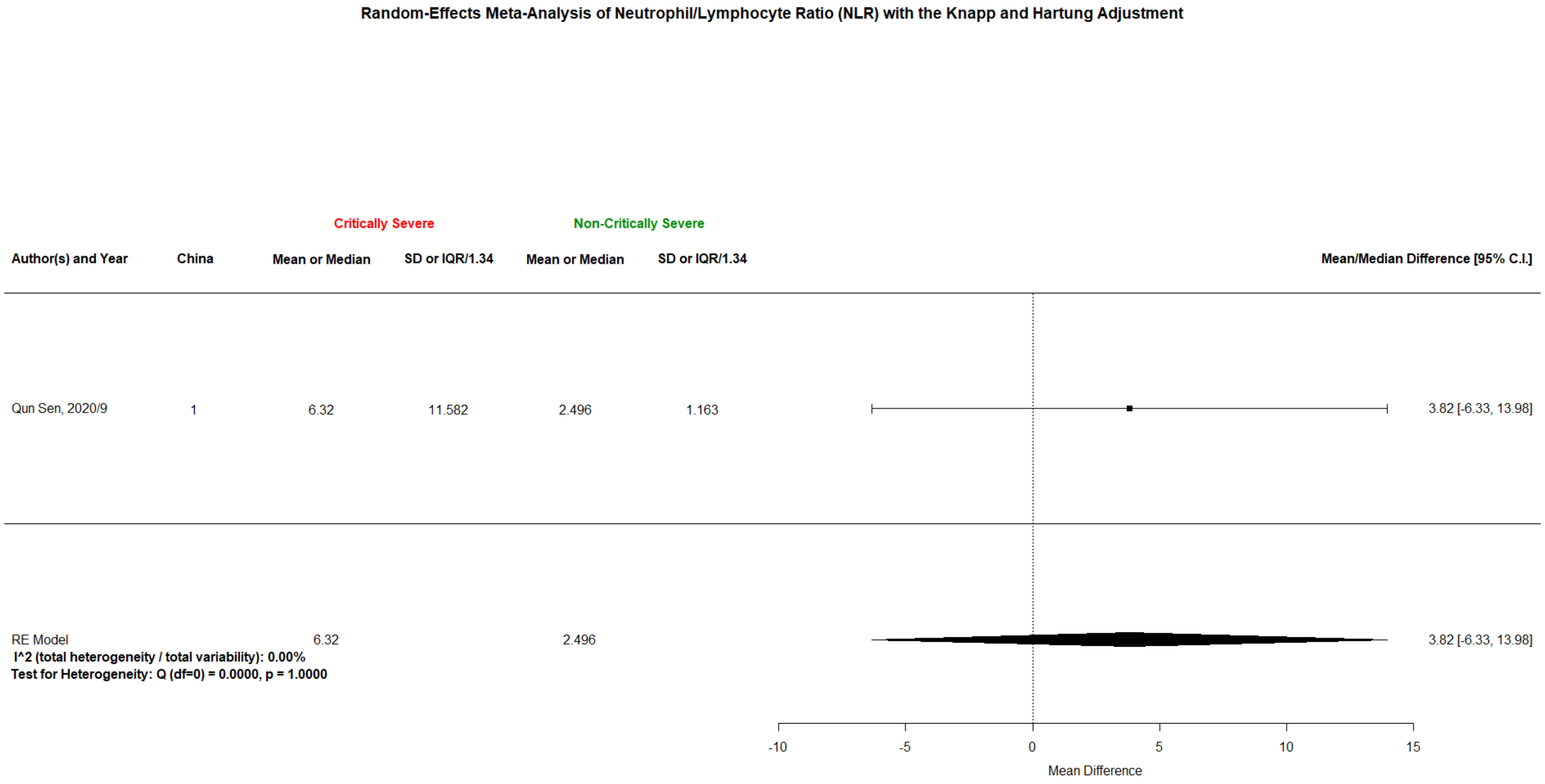

Figure S16. Forest plot of mean/median differences in platelet count (×10^9/L) between COVID-19 patients with critically severe or non-critically severe status

Random-Effects Meta-Analysis of Platelet Count with the Knapp and Hartung Adjustment

Author(s) and Year	China	Critically Severe Mean or Median	Critically Severe SD or IQR/1.34	Non-Critically Severe Mean or Median	Non-Critically Severe SD or IQR/1.34	Mean/Median Difference [95% C.I.]
Almazeedi Sulaiman, 2020/7	0	201	79	239	75	-38.00 [-62.32, -13.68]
Fan Bingwen Eugene. et al, 2020/3	0	217	109.701	201	79.104	16.00 [-58.51, 90.51]
Ferguson Jessica, 2020/8	0	183	69.403	198	76.866	-15.00 [-51.42, 21.42]
Hong Kyung Soo, 2020/4	0	186.7	64.9	245	107.5	-58.30 [-100.33, -16.27]
Huang Chaolin et al, 2020/2	1	196	73.134	149	98.507	47.00 [-6.96, 100.96]
Israelsen Simone Bastrup et al, 2020/6	0	197	54.478	187	69.403	10.00 [-13.39, 33.39]
Jiang Hao, 2020/7	1	194.5	91.418	189	76.866	5.50 [-41.86, 52.86]
Khamis Faryal, 2020/6	0	253	81.343	215	70.896	38.00 [-1.42, 77.42]
Li Huan, 2020/3	1	202.5	85.19	206.804	83.046	-4.30 [-48.70, 40.09]
Ortiz-Brizuela Edgar, 2020/5	0	213	80.597	193	52.239	20.00 [-10.90, 50.90]
Shahriarirad Reza, 2020	0	220	108.82	220.98	105.87	-0.98 [-68.49, 66.53]
Sun Ying, 2020/4	1	193.56	75.86	184.448	58.825	9.11 [-42.87, 61.10]
Wang Dawei et al, 2020/2	1	142	61.94	165	47.015	-23.00 [-45.20, -0.80]
Yang Luhuan, 2020/5	1	145	42.164	155	50	-10.00 [-27.08, 7.08]
Zhang Shan-Yan, 2020	1	146	44.403	181.121	54.837	-35.12 [-56.58, -13.66]
RE Model		186.452		193.037		-6.58 [-21.99, 8.82]

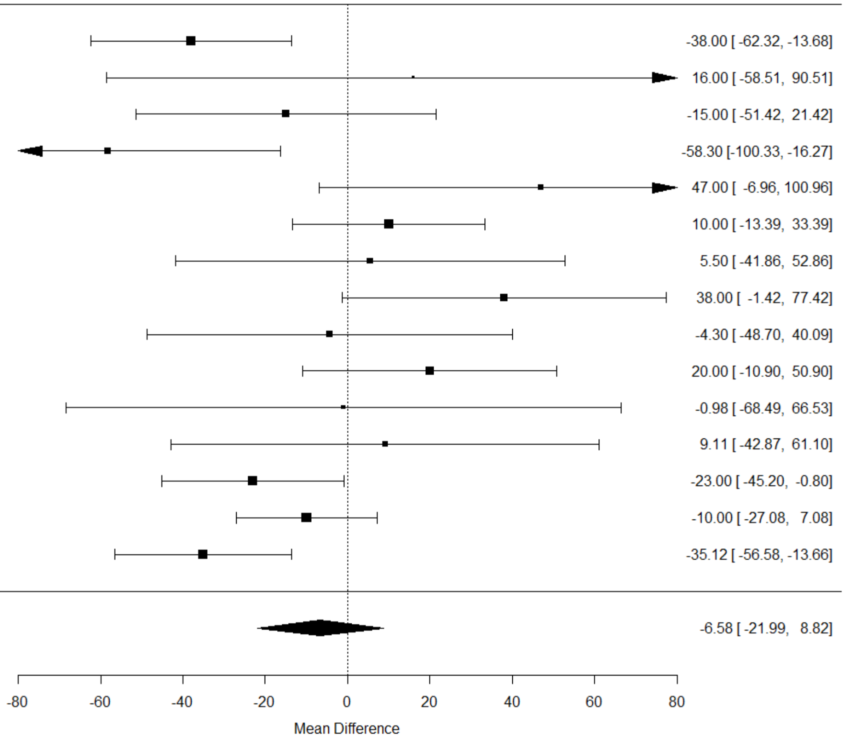

I^2 (total heterogeneity / total variability): 63.19%
Test for Heterogeneity: Q (df=14) = 34.6872, p = 0.0016

Figure S17. Forest plot of mean/median differences in alanine aminotransferase (ALT) (U/L) between COVID-19 patients with critically severe or non-critically severe status

Random-Effects Meta-Analysis of Alanine Aminotransferase (ALT) with the Knapp and Hartung Adjustment

		Critically Severe		Non-Critically Severe		
Author(s) and Year	China	Mean or Median	SD or IQR/1.34	Mean or Median	SD or IQR/1.34	Mean/Median Difference [95% C.I.]
Almazeedi Sulaiman, 2020/7	0	52	73	33	32	19.00 [-3.16, 41.16]
Ferguson Jessica, 2020/8	0	49	17.91	35	21.642	14.00 [4.31, 23.69]
Hong Kyung Soo, 2020/4	0	58.8	93.6	30.1	26.3	28.70 [-22.49, 79.89]
Huang Chaolin et al, 2020/2	1	49	64.179	27	15.299	22.00 [-13.34, 57.34]
Israelsen Simone Bastrup et al, 2020/6	0	36	37.313	29	19.403	7.00 [-7.42, 21.42]
Jiang Hao, 2020/7	1	37.8	15.522	36.7	4.701	1.10 [-4.07, 6.27]
Ortiz-Brizuela Edgar, 2020/5	0	42	20.149	34	23.993	8.00 [-0.58, 16.58]
Shahriarirad Reza, 2020	0	32	0	36.42	39.36	-4.42 [-12.06, 3.22]
Sun Ying, 2020/4	1	83.11	140.49	31.705	26.857	51.40 [-40.66, 143.47]
Wang Dawei et al, 2020/2	1	35	28.358	23	15.672	12.00 [2.25, 21.75]
Wang Feng, 2020/4	1	89.1	35.7	73.02	35.999	16.08 [-4.56, 36.72]
Yan Xiquan, 2020/8	1	24.76	11.76	28.378	21.387	-3.62 [-10.53, 3.29]
Yang Luhuan, 2020/5	1	30	41.791	26	12.687	4.00 [-11.33, 19.33]
Zhang Shan-Yan, 2020	1	30.5	12.537	24.9	10.287	5.60 [-0.40, 11.60]
Zhao Zirun, 2020/7	0	37	23.134	29	24.627	8.00 [3.95, 12.05]
RE Model		37.607		31.811		5.80 [1.79, 9.80]

I^2 (total heterogeneity / total variability): 53.53%
Test for Heterogeneity: Q (df=14) = 27.3356, p = 0.0174

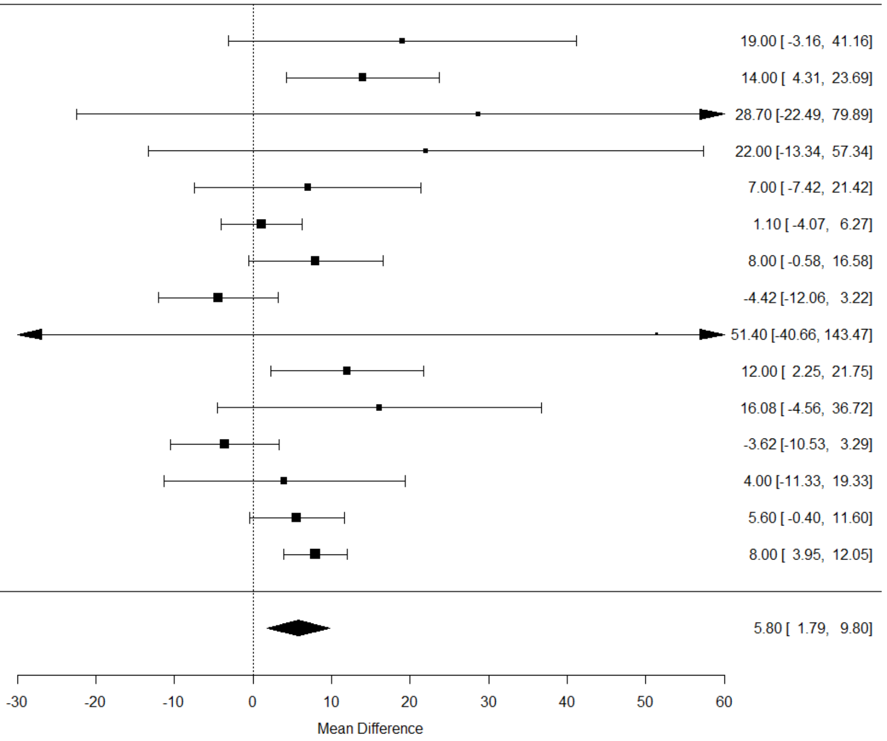

Figure S18. Forest plot of mean/median differences in aspartate aminotransferase (AST) (U/L) between COVID-19 patients with critically severe or non-critically severe status

Random-Effects Meta-Analysis of Aspartate Aminotransferase (AST) with the Knapp and Hartung Adjustment

		Critically Severe		Non-Critically Severe		
Author(s) and Year	China	Mean or Median	SD or IQR/1.34	Mean or Median	SD or IQR/1.34	Mean/Median Difference [95% C.I.]
Almazeedi Sulaiman, 2020/7	0	65	92	30	20	35.00 [7.15, 62.85]
Hong Kyung Soo, 2020/4	0	100.3	97	37.5	26.6	62.80 [9.77, 115.83]
Huang Chaolin et al, 2020/2	1	44	29.851	34	12.313	10.00 [-6.86, 26.86]
Jiang Hao, 2020/7	1	26.5	11.716	26.1	7.09	0.40 [-4.59, 5.39]
Ortiz-Brizuela Edgar, 2020/5	0	56	25.373	34	19.776	22.00 [12.06, 31.94]
Shahriarirad Reza, 2020	0	49	0	47	33.33	2.00 [-4.47, 8.47]
Sun Ying, 2020/4	1	95.67	153.65	31.759	18.262	63.91 [-36.59, 164.41]
Wang Dawei et al, 2020/2	1	52	29.851	29	12.687	23.00 [12.94, 33.06]
Yan Xiquan, 2020/8	1	33.44	11.23	28.011	13.381	5.43 [-0.77, 11.63]
Yang Luhuan, 2020/5	1	41	23.134	31	10.448	10.00 [1.44, 18.56]
Zhang Shan-Yan, 2020	1	30.5	12.537	25.035	10.287	5.46 [-0.54, 11.47]
RE Model		42.615		31.602		11.01 [2.94, 19.08]

I^2 (total heterogeneity / total variability): 77.54%
Test for Heterogeneity: Q (df=10) = 37.9053, p < .0001

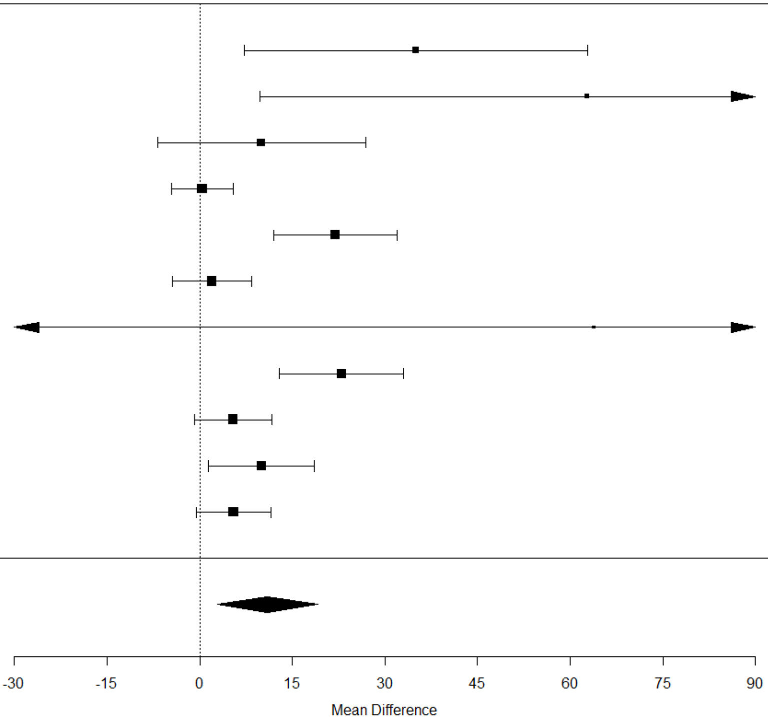

Figure S19. Forest plot of mean/median differences in total bilirubin (µmol/L) between COVID-19 patients with critically severe or non-critically severe status

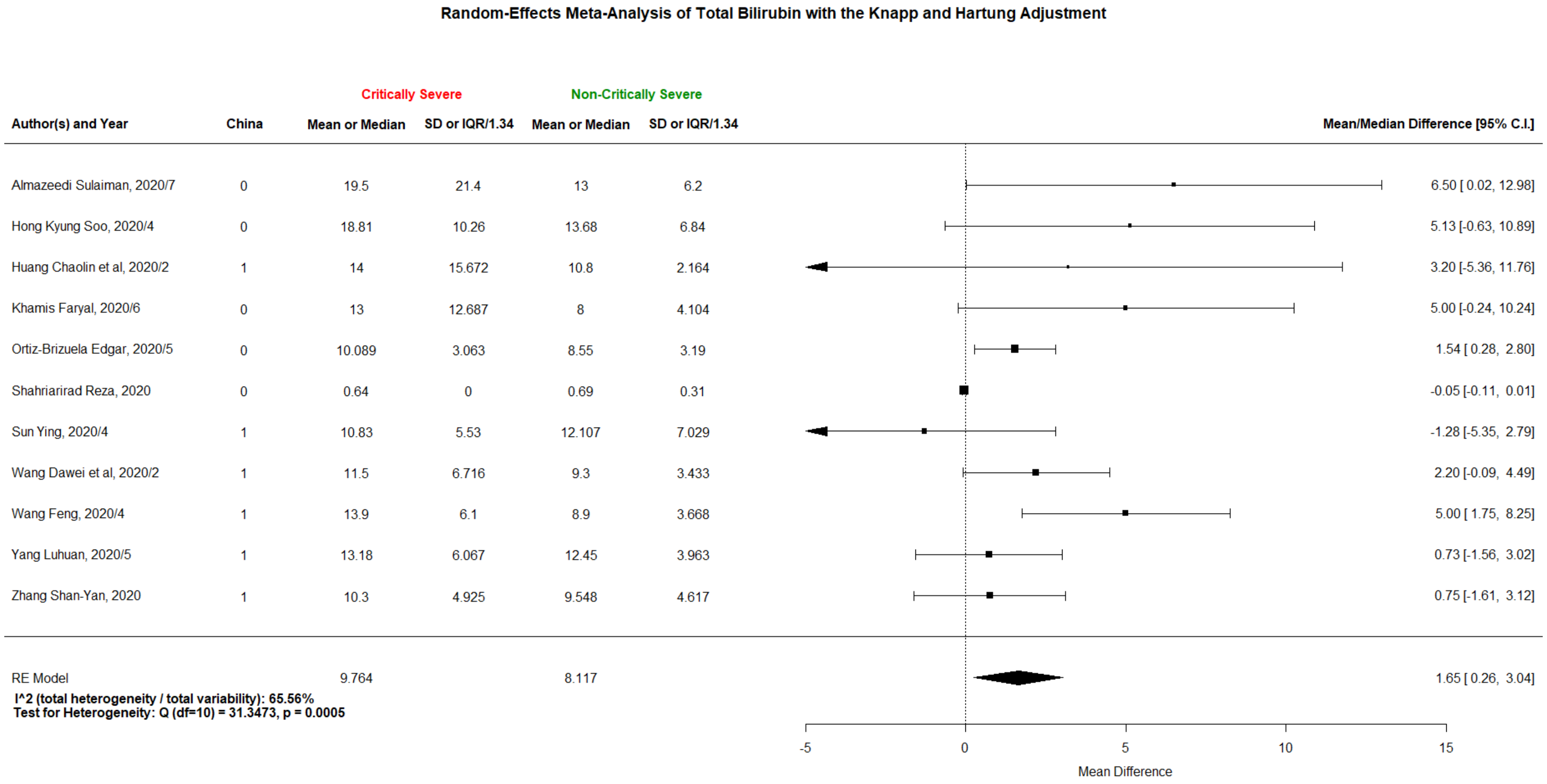

Figure S20. Forest plot of mean/median differences in lactate dehydrogenase (LDH) (U/L) between COVID-19 patients with critically severe or non-critically severe status

Random-Effects Meta-Analysis of Lactate Dehydrogenase with the Knapp and Hartung Adjustment

		Critically Severe		Non-Critically Severe		
Author(s) and Year	China	Mean or Median	SD or IQR/1.34	Mean or Median	SD or IQR/1.34	Mean/Median Difference [95% C.I.]
Fan Bingwen Eugene. et al, 2020/3	0	1684	744.776	401	120.149	1283.00 [795.44, 1770.56]
Ferguson Jessica, 2020/8	0	430	166.418	344	143.284	86.00 [4.68, 167.32]
Hong Kyung Soo, 2020/4	0	1272.6	542.1	555.5	184	717.10 [419.83, 1014.37]
Huang Chaolin et al, 2020/2	1	400	190.299	281	92.537	119.00 [10.02, 227.98]
Israelsen Simone Bastrup et al, 2020/6	0	443	235.821	280	111.194	163.00 [72.26, 253.74]
Khamis Faryal, 2020/6	0	444	205.97	293	92.537	151.00 [63.63, 238.37]
Ortiz-Brizuela Edgar, 2020/5	0	488	188.06	266.5	99.254	221.50 [150.61, 292.39]
Sun Ying, 2020/4	1	376.89	161.55	231.11	71.576	145.78 [38.52, 253.04]
Wang Dawei et al, 2020/2	1	435	219.403	212	89.552	223.00 [149.25, 296.75]
Wang Feng, 2020/4	1	542.5	220.9	353	130.24	189.50 [72.03, 306.97]
Yan Xiquan, 2020/8	1	510.06	733.24	220.661	75.87	289.40 [-94.85, 673.65]
Yang Luhuan, 2020/5	1	342	155.224	217	67.91	125.00 [67.60, 182.40]
Zhang Shan-Yan, 2020	1	320.5	74.776	210.411	68.065	110.09 [74.22, 145.96]
Zhao Zirun, 2020/7	0	451	199.254	316	112.687	135.00 [104.92, 165.08]
RE Model		433.064		271.892		161.17 [97.86, 224.48]

I^2 (total heterogeneity / total variability): 57.03%
Test for Heterogeneity: Q (df=13) = 51.8972, p < 0.0001

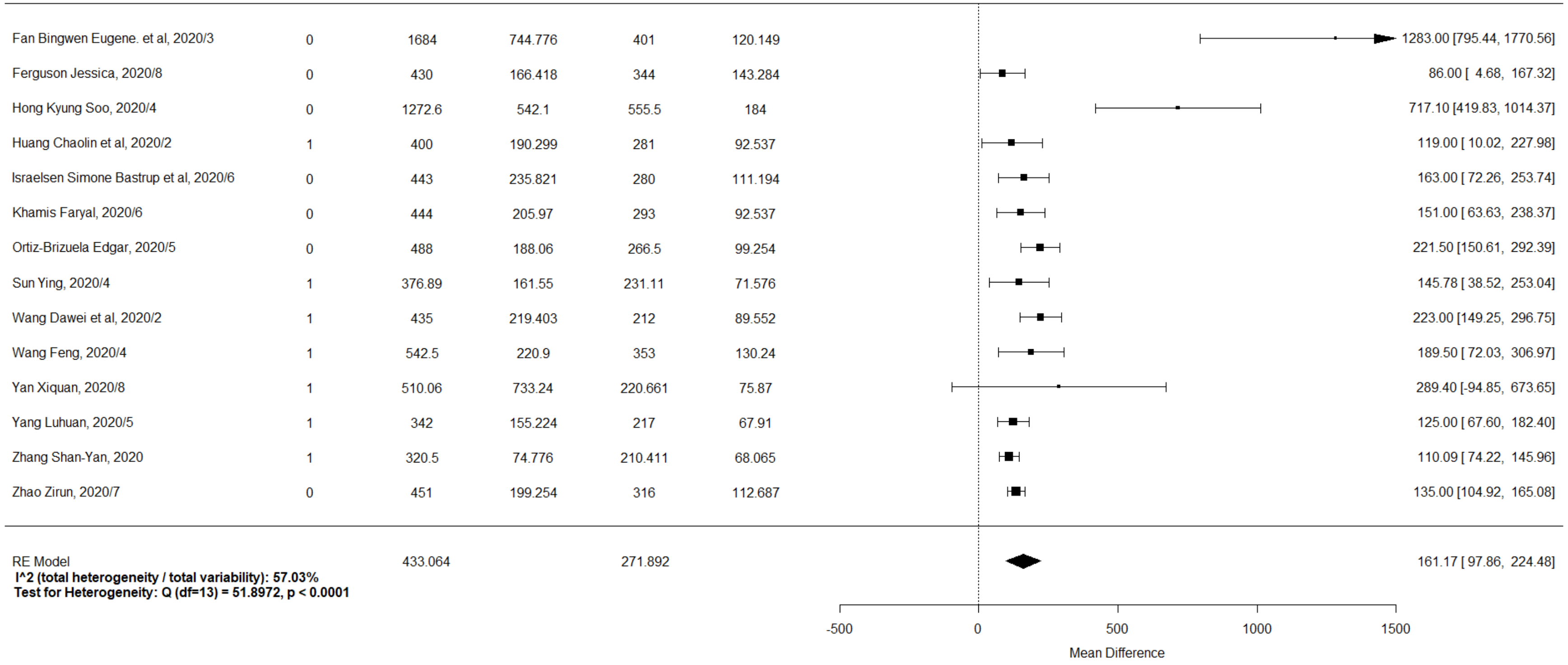

Figure S21. Forest plot of mean/median differences in D-dimer (mg/L or µg/mL) between COVID-19 patients with critically severe or non-critically severe status

Random-Effects Meta-Analysis of D-dimer with the Knapp and Hartung Adjustment

Author(s) and Year	China	Critically Severe: Mean or Median	Critically Severe: SD or IQR/1.34	Non-Critically Severe: Mean or Median	Non-Critically Severe: SD or IQR/1.34	Mean/Median Difference [95% C.I.]
Almazeedi Sulaiman, 2020/7	0	1.357	1.392	0.259	0.206	1.10 [0.68, 1.52]
Huang Chaolin et al, 2020/2	1	2.4	10.299	0.5	0.373	1.90 [-3.70, 7.50]
Jiang Hao, 2020/7	1	0.365	1.098	0	0.005	0.36 [0.04, 0.69]
Khamis Faryal, 2020/6	0	2.3	2.687	0.4	0.373	1.90 [0.82, 2.98]
Lendorf Maria Elisabeth, 2020	0	1.2	0.597	1.2	1.343	0.00 [-0.38, 0.38]
Lv Zhihua, 2020/5	1	11.97	25	4.104	9.159	7.87 [2.41, 13.32]
Ortiz-Brizuela Edgar, 2020/5	0	0.729	0.598	0.454	0.276	0.27 [0.05, 0.50]
Sun Ying, 2020/4	1	1.95	2.38	0.84	1.406	1.11 [-0.49, 2.71]
Wang Dawei et al, 2020/2	1	0.414	0.846	0.166	0.137	0.25 [-0.03, 0.53]
Wang Feng, 2020/4	1	6.9	8.4	2.84	5.207	4.06 [-0.43, 8.55]
Yan Xiquan, 2020/8	1	1.76	3.34	0.348	0.346	1.41 [-0.34, 3.16]
Yang Luhuan, 2020/5	1	1.1	0.884	0.497	0.328	0.60 [0.28, 0.93]
Zhao Zirun, 2020/7	0	2.138	1.716	1.663	1.225	0.47 [0.21, 0.74]
RE Model		1.186		0.617		0.57 [0.18, 0.96]

I^2 (total heterogeneity / total variability): 73.35%
Test for Heterogeneity: Q (df=12) = 38.3084, p = 0.0001

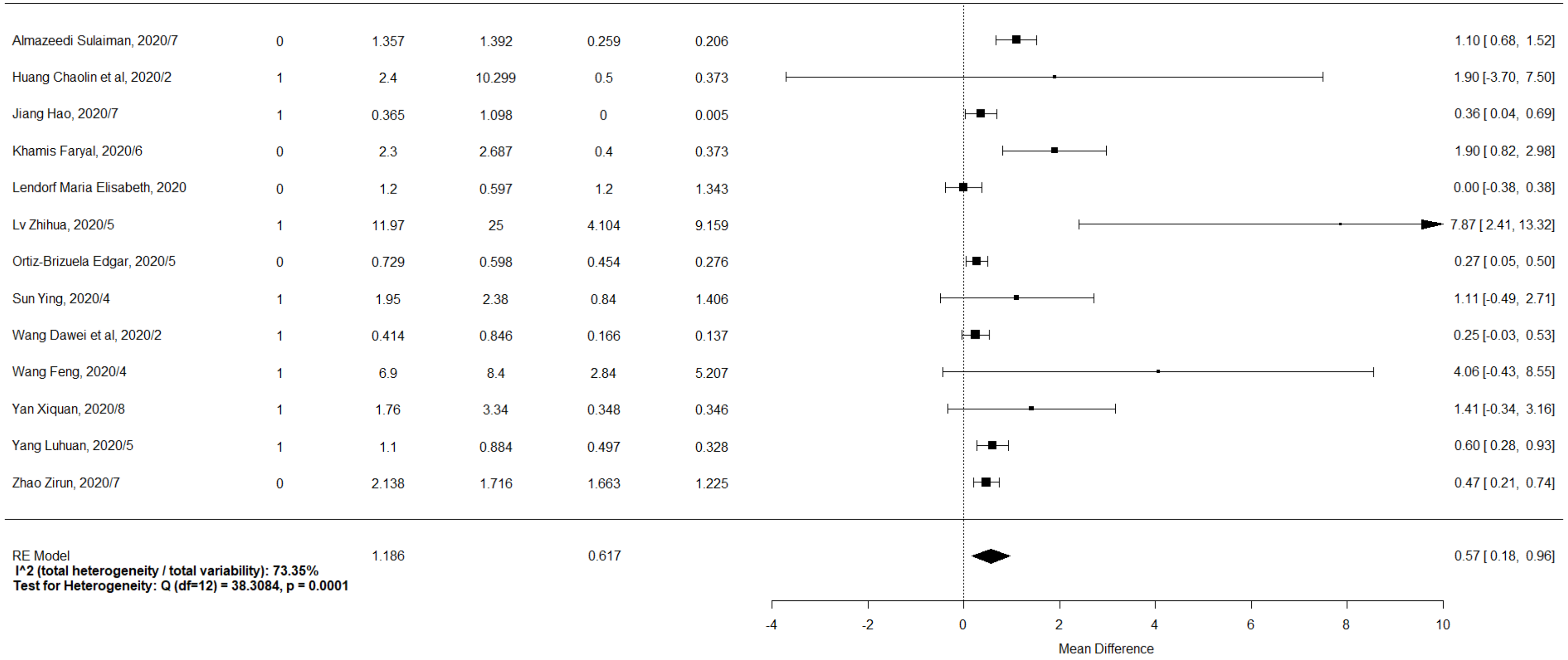

Figure S22. Forest plot of mean/median differences in C-reactive protein (CRP) (mg/L) between COVID-19 patients with critically severe or non-critically severe status

Random-Effects Meta-Analysis of C-Reactive Protein (CRP) with the Knapp and Hartung Adjustment

		Critically Severe		Non-Critically Severe		
Author(s) and Year	China	Mean or Median	SD or IQR/1.34	Mean or Median	SD or IQR/1.34	Mean/Median Difference [95% C.I.]
Almazeedi Sulaiman, 2020/7	0	121	120	17	31	104.00 [67.66, 140.34]
Hong Kyung Soo, 2020/4	0	177	95	42	67	135.00 [81.43, 188.57]
Israelsen Simone Bastrup et al, 2020/6	0	130	80.597	56	67.91	74.00 [41.69, 106.31]
Jiang Hao, 2020/7	1	9.9	134.627	8	57.985	1.90 [-47.53, 51.33]
Khamis Faryal, 2020/6	0	148	79.851	37	57.463	111.00 [74.31, 147.69]
Lendorf Maria Elisabeth, 2020	0	109	69.403	54	52.985	55.00 [22.69, 87.31]
Li Huan, 2020/3	1	97.44	58.6	49.064	38.848	48.38 [18.80, 77.95]
Lv Zhihua, 2020/5	1	65.23	54.33	41.63	49.127	23.60 [10.59, 36.61]
Ortiz-Brizuela Edgar, 2020/5	0	137.4	59.403	65	78.507	72.40 [46.31, 98.49]
Shahriarirad Reza, 2020	0	44	24.74	33.33	19.79	10.67 [-4.45, 25.79]
Sun Ying, 2020/4	1	30.96	29.46	17.699	33.809	13.26 [-7.99, 34.52]
Wang Feng, 2020/4	1	114.9	62.5	68.88	66.388	46.02 [9.43, 82.61]
Yan Xiquan, 2020/8	1	66.01	54.34	16.13	26.037	49.88 [21.16, 78.60]
Yang Luhuan, 2020/5	1	68.8	40.881	18.7	23.582	50.10 [34.81, 65.39]
Zhang Shan-Yan, 2020	1	45.5	52.388	8.773	13.418	36.73 [11.81, 61.65]
Zhao Zirun, 2020/7	0	132	102.239	60	69.403	72.00 [56.11, 87.89]
RE Model		91.31		37.183		54.13 [35.47, 72.78]

I^2 (total heterogeneity / total variability): 86.43%
Test for Heterogeneity: Q (df=15) = 94.7421, p < 0.0001

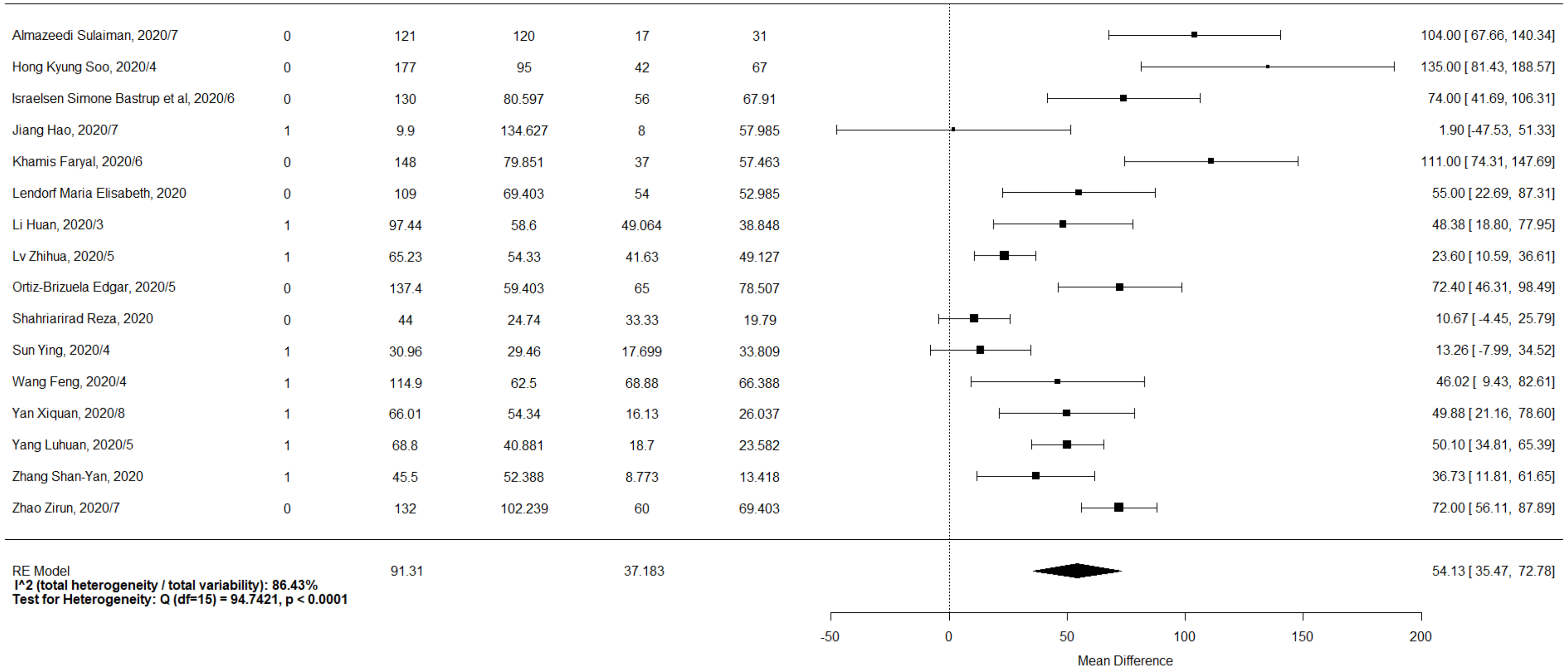

Figure S23. Forest plot of mean/median differences in procalcitonin (PCT) (×100 ng/mL) between COVID-19 patients with critically severe or non-critically severe status

Random-Effects Meta-Analysis of Procalcitonin (PCT) with the Knapp and Hartung Adjustment

Author(s) and Year	China	Critically Severe: Mean or Median	Critically Severe: SD or IQR/1.34	Non-Critically Severe: Mean or Median	Non-Critically Severe: SD or IQR/1.34	Mean/Median Difference [95% C.I.]
Almazeedi Sulaiman, 2020/7	0	0.79	1.9	0.41	5.2	0.38 [-0.27, 1.03]
Hong Kyung Soo, 2020/4	0	2.7	7	0.2	1.3	2.50 [-1.32, 6.32]
Huang Chaolin et al, 2020/2	1	0.1	0.224	0.1	0	0.00 [-0.12, 0.12]
Li Huan, 2020/3	1	0.44	0.512	0.109	0.317	0.33 [0.07, 0.59]
Lv Zhihua, 2020/5	1	0.22	0.38	0.213	0.738	0.01 [-0.11, 0.13]
Ortiz-Brizuela Edgar, 2020/5	0	0.56	0.239	0.05	0.045	0.51 [0.42, 0.60]
Wang Feng, 2020/4	1	0.8	1.2	0.18	0.489	0.62 [-0.00, 1.24]
Zhang Shan-Yan, 2020	1	0.1	0.149	0.1	0.075	0.00 [-0.07, 0.07]
Zhao Zirun, 2020/7	0	0.29	0.425	0.13	0.127	0.16 [0.10, 0.22]
RE Model		0.333		0.134		0.20 [0.01, 0.39]

I^2 (total heterogeneity / total variability): 92.07%
Test for Heterogeneity: Q (df=8) = 99.7276, p < 0.0001

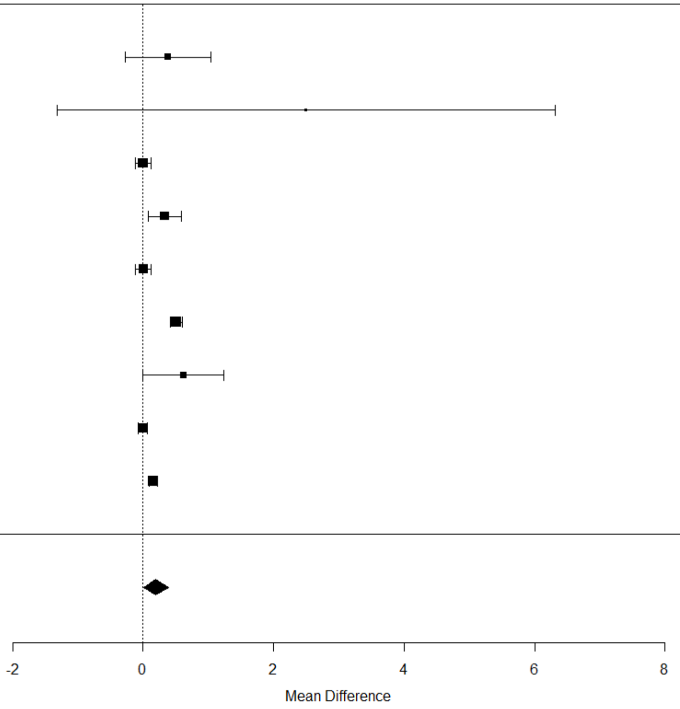

Figure S24. Forest plot of mean/median differences in hypersensitive troponin I (hs-cTnI) (×100 ng/mL) between COVID-19 patients with critically severe or non-critically severe status

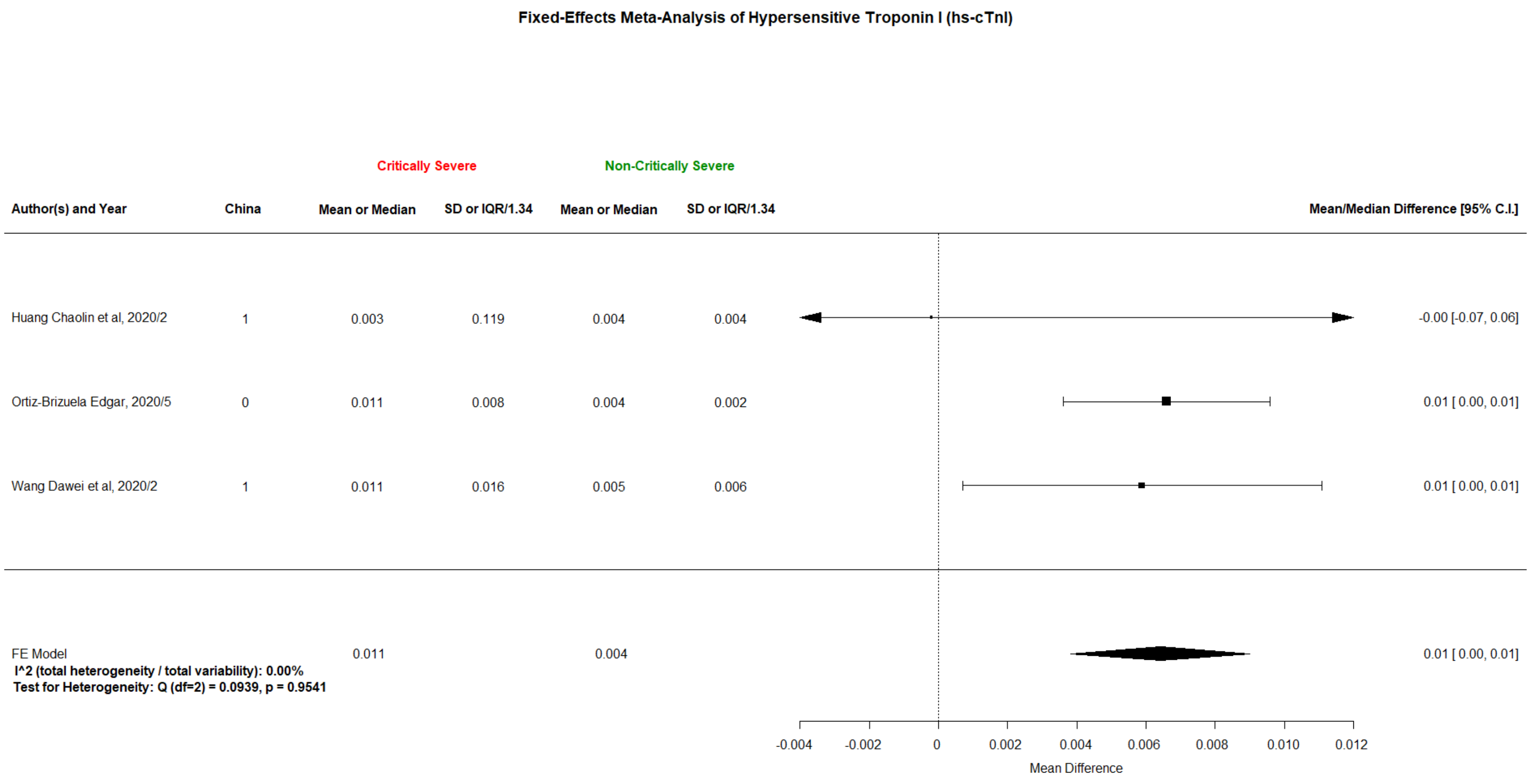

Figure S25. Forest plot of mean/median differences in white blood cell count ($\times 10^9$/L) between COVID-19 patients with dead or alive outcome

Random-Effects Meta-Analysis of White Blood Cell Count with the Knapp and Hartung Adjustment

Author(s) and Year	China	Dead: Mean or Median	Dead: SD or IQR/1.34	Alive: Mean or Median	Alive: SD or IQR/1.34	Mean/Median Difference [95% C.I.]
Almazeedi Sulaiman, 2020/7	0	8.4	3.1	6.1	2.1	2.30 [0.90, 3.70]
Bahl Amit, 2020/9	0	6.8	3.433	6.1	2.463	0.70 [0.30, 1.10]
Berenguer Juan, 2020/7	0	6.9	3.336	5.635	2.306	1.27 [1.05, 1.48]
Bonetti Graziella et al, 2020/4	0	6.64	4.873	5.605	2.403	1.03 [-0.23, 2.30]
Botero Diana Maria Ronderos, 2020	0	9.4	4.8	8.14	3.7	1.26 [-0.72, 3.24]
Chen Tao, 2020/3	1	10.2	5.522	5	1.94	5.20 [4.14, 6.26]
Ciceri Fabio, 2020/6	0	7.5	4.701	6.313	2.651	1.19 [0.20, 2.18]
Gayam Vijay, 2020/7	0	8.8	4.403	7.1	3.657	1.70 [0.83, 2.57]
Giacomelli Andrea, 2020/5	0	6.7	3.134	5.5	2.015	1.20 [0.27, 2.13]
Javanlian Mostafa, 2020	0	8.3	9.627	5.3	10.522	3.00 [-1.90, 7.90]
Lee Ji Yeon et al, 2020/6	0	7.855	5.847	5.135	1.933	2.72 [0.12, 5.32]
Liu Jiao, 2020	1	15.5	9.701	6	2.687	9.50 [7.97, 11.03]
Shahriarirad Reza, 2020	0	6.83	2.94	6	2.47	0.83 [-1.15, 2.81]
Sun Haiying, 2020	1	10.15	5.366	5.37	1.694	4.78 [3.78, 5.78]
Wang Ruoran, 2020/8	1	9.04	4.567	5.43	2.306	3.61 [2.57, 4.65]
Zerah Lorene, 2020/8	0	9.5	4.478	8	3.881	1.50 [0.86, 2.14]
Zhang Chi, 2020/5	1	4.94	2.81	4.721	1.537	0.22 [-2.98, 3.42]
Zhang Jin-jin, 2020/7	1	6.69	4.507	4.318	2.13	2.37 [1.08, 3.66]
RE Model		8.391		5.918		2.47 [1.36, 3.59]

I^2 (total heterogeneity / total variability): 96.44%
Test for Heterogeneity: Q (df=17) = 235.9144, p < 0.0001

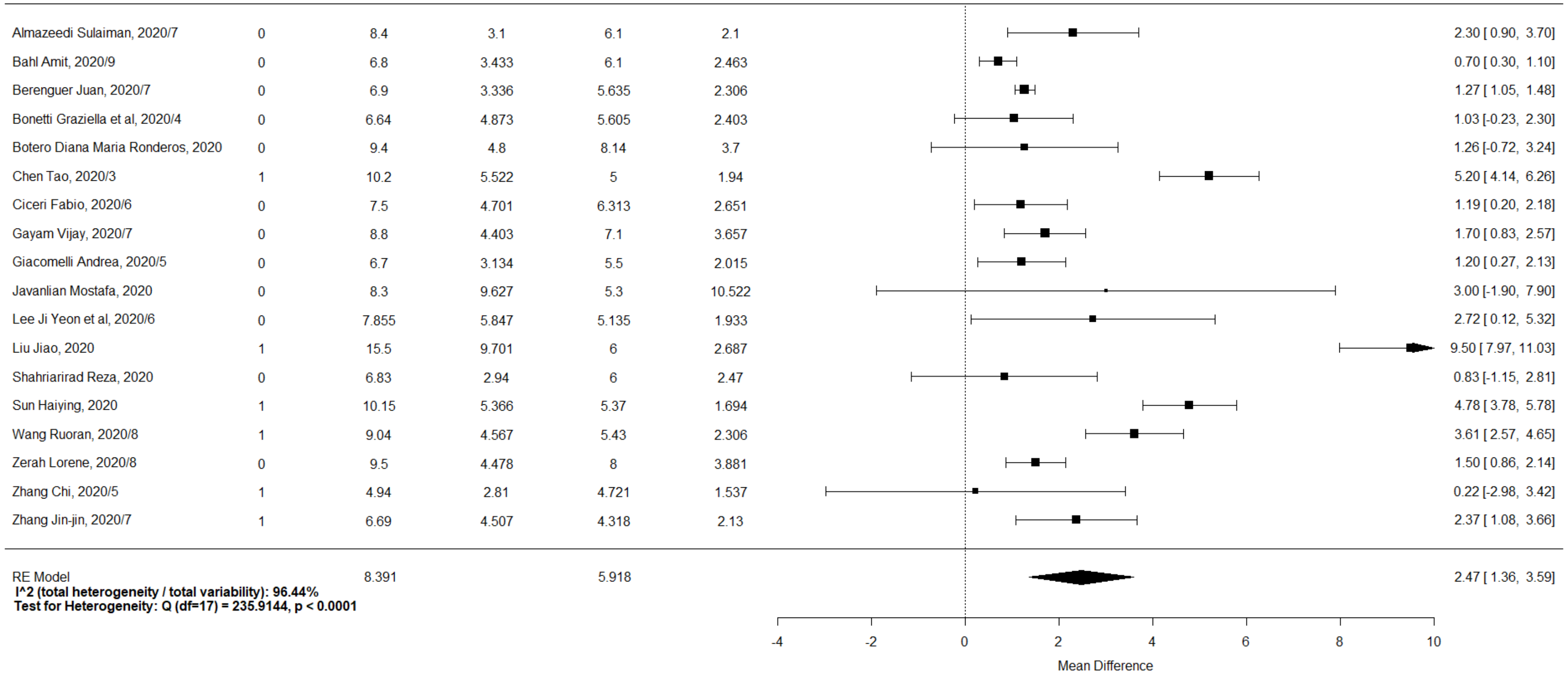

Figure S26. Forest plot of mean/median differences in neutrophil count ($\times 10^9$/L) between COVID-19 patients with dead or alive outcome

Random-Effects Meta-Analysis of Neutrophil Count with the Knapp and Hartung Adjustment

Author(s) and Year	China	Dead: Mean or Median	Dead: SD or IQR/1.34	Alive: Mean or Median	Alive: SD or IQR/1.34	Mean/Median Difference [95% C.I.]
Berenguer Juan, 2020/7	0	5.3	3.112	3.92	2.06	1.38 [1.18, 1.58]
Bonetti Graziella et al, 2020/4	0	5.59	3.933	4.13	2.291	1.46 [0.40, 2.52]
Botero Diana Maria Ronderos, 2020	0	8.1	4.5	6.5	3.7	1.60 [-0.27, 3.47]
Brill Simon E., 2020	0	6.6	4.158	5.32	3.239	1.28 [0.54, 2.02]
Chen Lei et al, 2020/6	1	7	4.478	3	1.493	4.00 [3.39, 4.61]
Chen Tao, 2020/3	1	9	5.448	3.2	1.567	5.80 [4.77, 6.83]
Ciceri Fabio, 2020/6	0	6.3	4.03	4.751	2.651	1.55 [0.69, 2.41]
Gayam Vijay, 2020/7	0	7.2	4.216	5.6	3.358	1.60 [0.78, 2.42]
Giacomelli Andrea, 2020/5	0	5.7	3.358	3.9	1.866	1.80 [0.81, 2.79]
Khalil K., 2020	0	5.3	3.582	4.7	2.537	0.60 [-0.40, 1.60]
Lee Ji Yeon et al, 2020/6	0	6.451	4.681	3.404	1.761	3.05 [0.96, 5.14]
Liu Jiao, 2020	1	14.7	7.761	4.1	2.537	10.60 [9.38, 11.82]
Wang Ruoran, 2020/8	1	7.41	4.746	3.39	1.896	4.02 [2.95, 5.09]
Zhang Chi, 2020/5	1	4.12	2.04	3.131	1.655	0.99 [-1.35, 3.33]
Zhang Jin-jin, 2020/7	1	5.97	4.299	2.896	2.015	3.07 [1.84, 4.30]
RE Model		6.995		4.134		2.86 [1.44, 4.29]

I^2 (total heterogeneity / total variability): 97.49%
Test for Heterogeneity: Q (df=14) = 352.3363, p < 0.0001

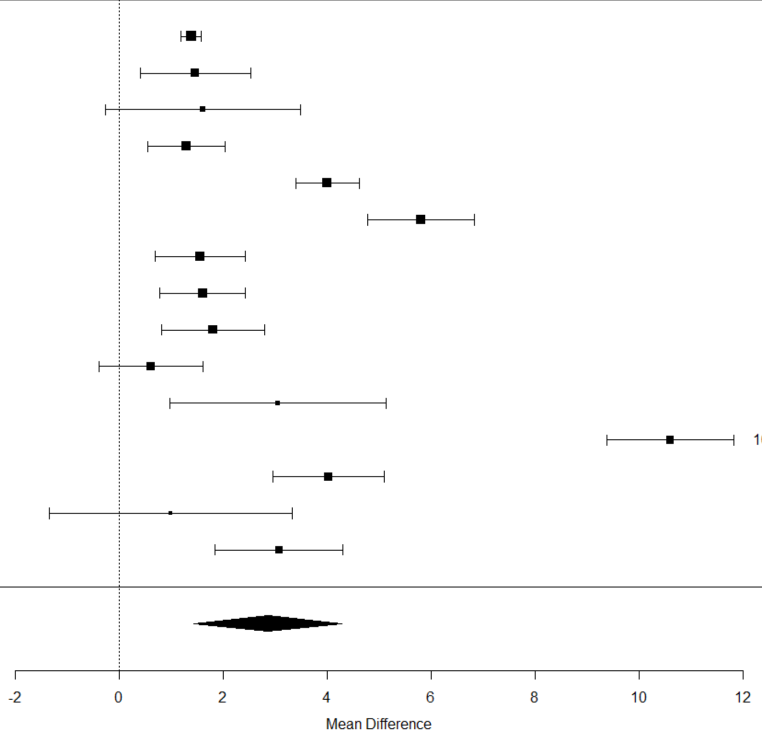

Figure S27. Forest plot of mean/median differences in neutrophil/lymphocyte ratio (NLR) between COVID-19 patients with dead or alive outcome

Random-Effects Meta-Analysis of Neutrophil/Lymphocyte Ratio (NLR) with the Knapp and Hartung Adjustment

		Dead		Alive		
**Author(s) and Year**	**China**	**Mean or Median**	**SD or IQR/1.34**	**Mean or Median**	**SD or IQR/1.34**	**Mean/Median Difference [95% C.I.]**
Berenguer Juan, 2020/7	0	6.6	5.746	3.9	2.985	2.70 [2.35, 3.05]
Chen Lei et al, 2020/6	1	11	10.448	3	1.493	8.00 [6.58, 9.42]
Ciceri Fabio, 2020/6	0	8.7	6.418	5.311	3.826	3.39 [2.03, 4.75]
Wang Ruoran, 2020/8	1	12.41	8.649	2.82	2.478	9.59 [7.65, 11.53]
Ye Wenjing, 2020	1	14.96	13.478	2.88	3.694	12.08 [8.39, 15.77]
Zhang Chi, 2020/5	1	7.85	4.14	3.934	4.77	3.92 [-0.89, 8.72]
Zhang Jin-jin, 2020/7	1	10.34	11.142	3.584	3.289	6.76 [3.61, 9.90]
RE Model		10.189		3.652		6.54 [3.32, 9.75]

I^2 (total heterogeneity / total variability): 94.80%
Test for Heterogeneity: Q (df=6) = 120.1687, p < 0.0001

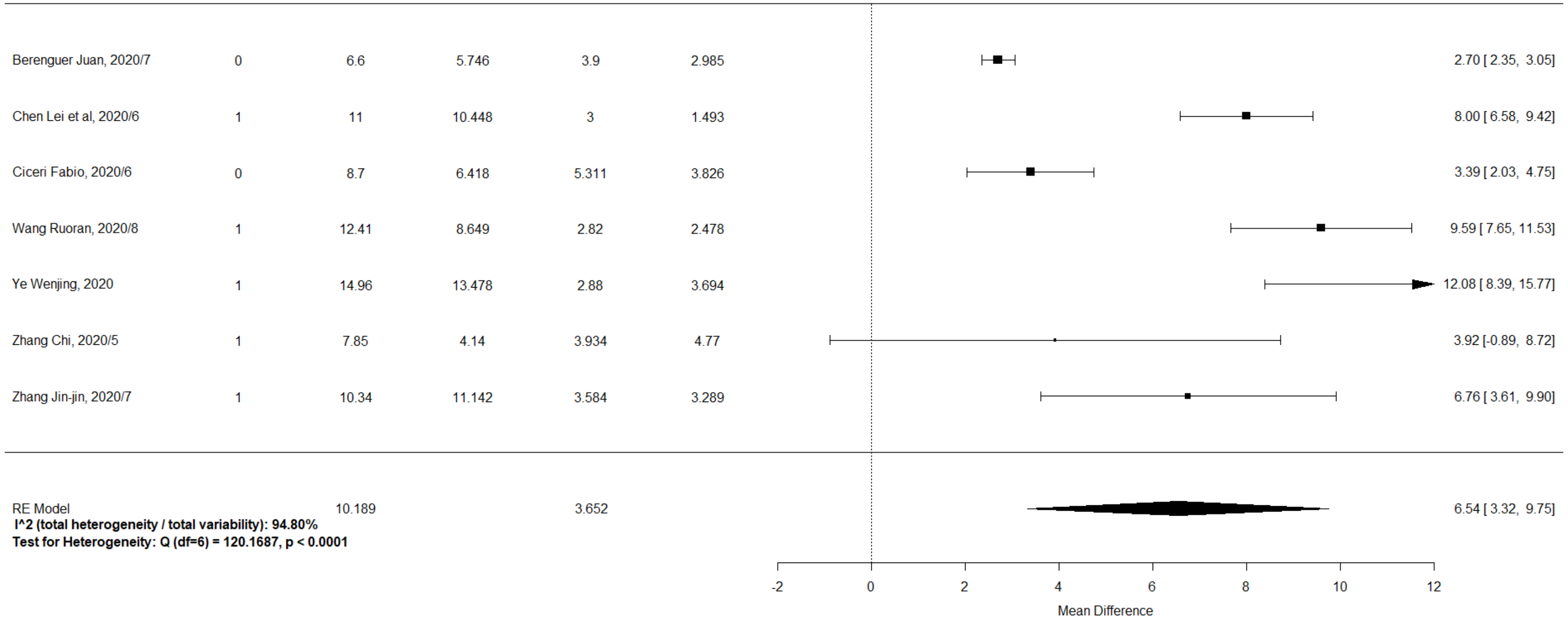

Figure S28. Forest plot of mean/median differences in platelet count ($\times10^9$/L) between COVID-19 patients with dead or alive outcome

Random-Effects Meta-Analysis of Platelet count with the Knapp and Hartung Adjustment

		Dead		Alive		
Author(s) and Year	China	Mean or Median	SD or IQR/1.34	Mean or Median	SD or IQR/1.34	Mean/Median Difference [95% C.I.]
Almazeedi Sulaiman, 2020/7	0	214	98	238	75	-24.00 [-68.29, 20.29]
Bahl Amit, 2020/9	0	182.5	63.433	199	69.776	-16.50 [-24.49, -8.51]
Berenguer Juan, 2020/7	0	168	67.91	181	64.179	-13.00 [-17.59, -8.41]
Bonetti Graziella et al, 2020/4	0	178	62.687	189	61.194	-11.00 [-31.25, 9.25]
Botero Diana Maria Ronderos, 2020	0	219.3	111	218.4	96.2	0.90 [-45.60, 47.40]
Chen Lei et al, 2020/6	1	163	82.09	207	79.851	-44.00 [-55.80, -32.20]
Chen Tao, 2020/3	1	156	80.224	198	71.642	-42.00 [-60.47, -23.53]
Ciceri Fabio, 2020/6	0	182	100.746	194.01	82.779	-12.01 [-34.24, 10.22]
Gayam Vijay, 2020/7	0	199	82.836	226	89.552	-27.00 [-44.64, -9.36]
Giacomelli Andrea, 2020/5	0	181	63.433	174	62.687	7.00 [-13.09, 27.09]
Javanlian Mostafa, 2020	0	210	247.015	183.5	258.209	26.50 [-97.99, 150.99]
Khalil K., 2020	0	183.5	75.746	214	76.119	-30.50 [-53.25, -7.75]
Liu Jiao, 2020	1	90.5	85.821	201	76.119	-110.50 [-124.70, -96.30]
Shahriarirad Reza, 2020	0	199.55	104.24	222.85	106.09	-23.30 [-94.39, 47.79]
Wang Ruoran, 2020/8	1	172	76.866	220	88.06	-48.00 [-67.26, -28.74]
Zerah Lorene, 2020/8	0	197	143.284	208	102.985	-11.00 [-30.67, 8.67]
Zhang Chi, 2020/5	1	204	121.87	215.781	96.883	-11.78 [-151.37, 127.81]
Zhang Jin-jin, 2020/7	1	156	64.179	173.613	64.478	-17.61 [-37.35, 2.12]
RE Model		175.046		201.869		-26.82 [-40.97, -12.67]

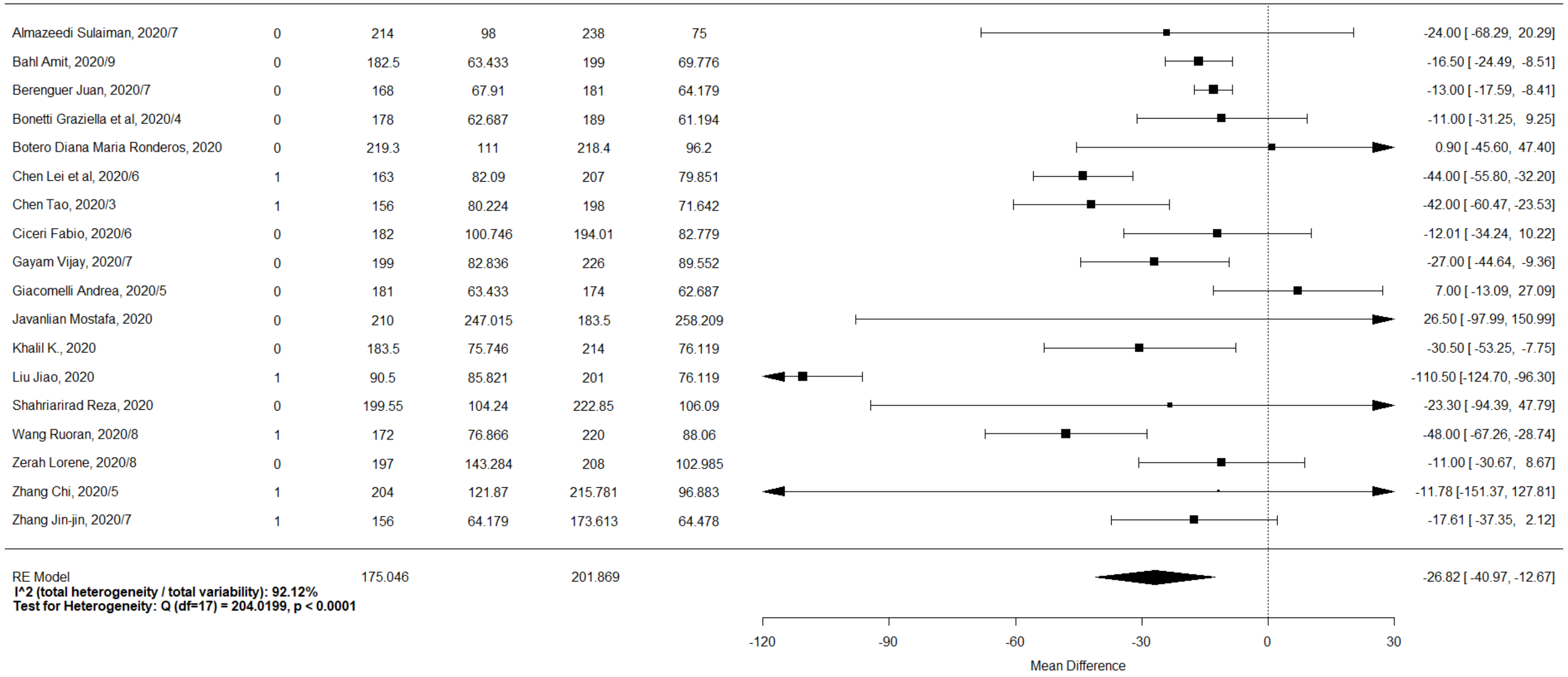

I^2 (total heterogeneity / total variability): 92.12%
Test for Heterogeneity: Q (df=17) = 204.0199, p < 0.0001

Figure S29. Forest plot of mean/median differences in alanine aminotransferase (ALT) (U/L) between COVID-19 patients with dead or alive outcome

Random-Effects Meta-Analysis of Alanine Aminotransferase (ALT) with the Knapp and Hartung Adjustment

		Dead		Alive		
**Author(s) and Year**	**China**	**Mean or Median**	**SD or IQR/1.34**	**Mean or Median**	**SD or IQR/1.34**	**Mean/Median Difference [95% C.I.]**
Almazeedi Sulaiman, 2020/7	0	68	98	32	32	36.00 [-8.11, 80.11]
Bahl Amit, 2020/9	0	30	27.612	28	20.522	2.00 [-1.22, 5.22]
Berenguer Juan, 2020/7	0	25	15.672	27	17.91	-2.00 [-3.12, -0.88]
Bonetti Graziella et al, 2020/4	0	34	19.403	31.5	18.657	2.50 [-3.72, 8.72]
Botero Diana Maria Ronderos, 2020	0	51.6	61	49.7	38.8	1.90 [-22.91, 26.71]
Chen Lei et al, 2020/6	1	58	81.343	36	31.343	22.00 [10.84, 33.16]
Chen Tao, 2020/3	1	28	21.642	20	12.836	8.00 [3.54, 12.46]
Ciceri Fabio, 2020/6	0	32	25.373	37.762	23.937	-5.76 [-11.51, -0.02]
Gayam Vijay, 2020/7	0	31.5	23.134	26	23.881	5.50 [0.65, 10.35]
Giacomelli Andrea, 2020/5	0	40	23.134	30	21.642	10.00 [2.75, 17.25]
Khalil K., 2020	0	27.5	24.403	30	23.881	-2.50 [-9.78, 4.78]
Liu Jiao, 2020	1	47	39.552	40	28.358	7.00 [0.58, 13.42]
Shahriarirad Reza, 2020	0	32	0	36.42	39.36	-4.42 [-11.98, 3.14]
Sun Haiying, 2020	1	28	17.164	22	16.418	6.00 [1.78, 10.22]
Wang Ruoran, 2020/8	1	27	19.403	24	17.164	3.00 [-1.65, 7.65]
Zhang Chi, 2020/5	1	13	6.08	39.309	36.876	-26.31 [-37.04, -15.58]
Zhang Jin-jin, 2020/7	1	46	26.866	20.6	15.28	25.40 [17.63, 33.17]
RE Model		33.972		30.239		3.73 [-2.29, 9.76]

I^2 (total heterogeneity / total variability): 94.87%
Test for Heterogeneity: Q (df=16) = 140.0373, p < 0.0001

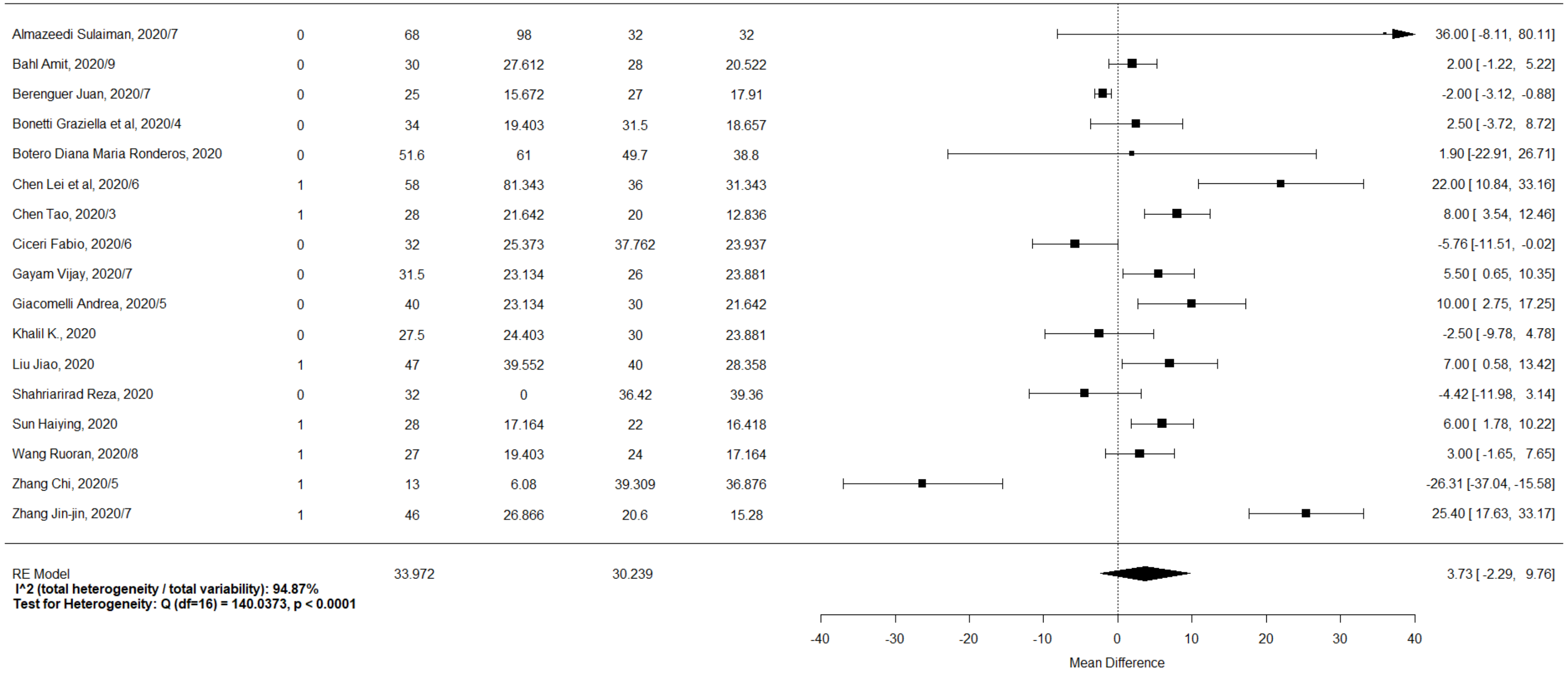

Figure S30. Forest plot of mean/median differences in aspartate aminotransferase (AST) (U/L) between COVID-19 patients with dead or alive outcome

Random-Effects Meta-Analysis of Aspartate Aminotransferase (AST) with the Knapp and Hartung Adjustment

Author(s) and Year	China	Dead: Mean or Median	Dead: SD or IQR/1.34	Alive: Mean or Median	Alive: SD or IQR/1.34	Mean/Median Difference [95% C.I.]
Almazeedi Sulaiman, 2020/7	0	31	23	68	98	-37.00 [-48.88, -25.12]
Bahl Amit, 2020/9	0	53	38.806	40	24.627	13.00 [8.56, 17.44]
Berenguer Juan, 2020/7	0	34	21.642	31	16.418	3.00 [1.60, 4.40]
Bonetti Graziella et al, 2020/4	0	61.5	29.104	45	25.373	16.50 [7.56, 25.44]
Botero Diana Maria Ronderos, 2020	0	80.3	90.6	60.6	42.7	19.70 [-16.55, 55.95]
Chen Lei et al, 2020/6	1	64	74.627	30	16.418	34.00 [23.83, 44.17]
Chen Tao, 2020/3	1	45	26.866	25	9.925	20.00 [14.81, 25.19]
Ciceri Fabio, 2020/6	0	50	35.821	44.381	23.987	5.62 [-2.06, 13.29]
Gayam Vijay, 2020/7	0	57.5	37.313	41	31.343	16.50 [9.14, 23.86]
Liu Jiao, 2020	1	58	48.507	33	15.672	25.00 [17.35, 32.65]
Shahriarirad Reza, 2020	0	47	0	47	33.33	0.00 [-6.41, 6.41]
Sun Haiying, 2020	1	45	25.373	28	14.179	17.00 [11.83, 22.17]
Wang Ruoran, 2020/8	1	43	24.627	24	12.687	19.00 [13.38, 24.62]
Zhang Chi, 2020/5	1	35	22.11	38.566	31.066	-3.57 [-29.53, 22.40]
Zhang Jin-jin, 2020/7	1	39	21.642	27.6	13.461	11.40 [5.11, 17.69]
RE Model		48.642		37.724		10.92 [1.96, 19.87]

I^2 (total heterogeneity / total variability): 96.68%
Test for Heterogeneity: Q (df=14) = 214.0638, p < 0.0001

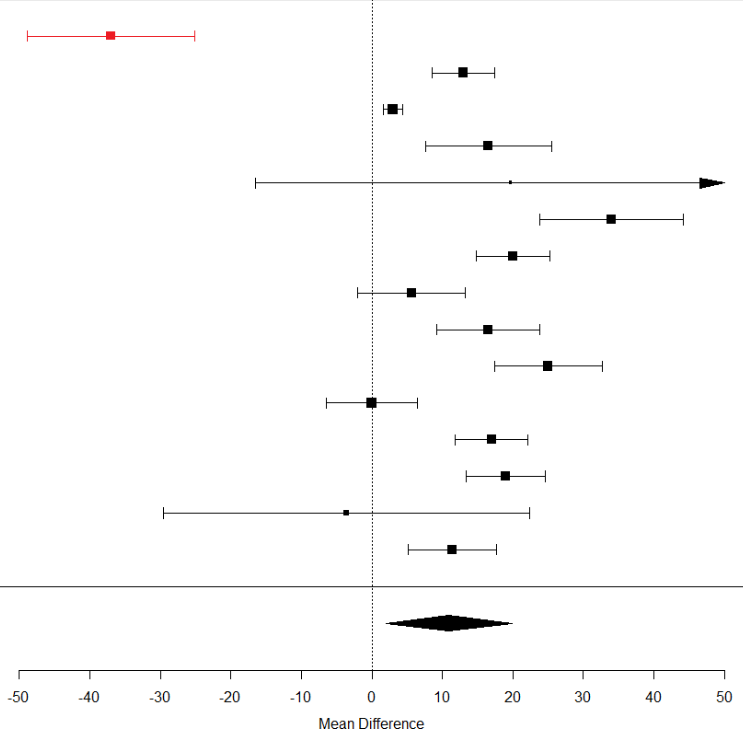

Figure S31. Forest plot of mean/median differences in total bilirubin (µmol/L) between COVID-19 patients with dead or alive outcome

Random-Effects Meta-Analysis of Total Bilirubin with the Knapp and Hartung Adjustment

		Dead		Alive		
Author(s) and Year	China	Mean or Median	SD or IQR/1.34	Mean or Median	SD or IQR/1.34	Mean/Median Difference [95% C.I.]
Almazeedi Sulaiman, 2020/7	0	21.7	22	12.8	6.4	8.90 [-1.00, 18.80]
Berenguer Juan, 2020/7	0	9.576	6.125	8.55	4.339	1.03 [0.64, 1.42]
Bonetti Graziella et al, 2020/4	0	11.7	8.06	10.6	3.881	1.10 [-0.98, 3.18]
Botero Diana Maria Ronderos, 2020	0	0.65	0.33	0.68	1.28	-0.03 [-0.28, 0.22]
Chen Lei et al, 2020/6	1	24	15.672	13	5.97	11.00 [8.85, 13.15]
Chen Tao, 2020/3	1	12.6	5.448	8.4	4.03	4.20 [3.02, 5.38]
Ciceri Fabio, 2020/6	0	9.576	5.998	9.063	4.729	0.51 [-0.80, 1.83]
Khalil K., 2020	0	10	5.97	9	4.478	1.00 [-0.68, 2.68]
Liu Jiao, 2020	1	24.9	14.552	12.4	4.701	12.50 [10.21, 14.79]
Shahriarirad Reza, 2020	0	0.38	0.21	0.73	0.29	-0.35 [-0.50, -0.20]
Zhang Chi, 2020/5	1	18.6	7.35	10.553	5.036	8.05 [-0.35, 16.44]
RE Model		12.212		8.334		3.88 [0.64, 7.12]

I^2 (total heterogeneity / total variability): 99.66%
Test for Heterogeneity: Q (df=10) = 320.8595, p < 0.0001

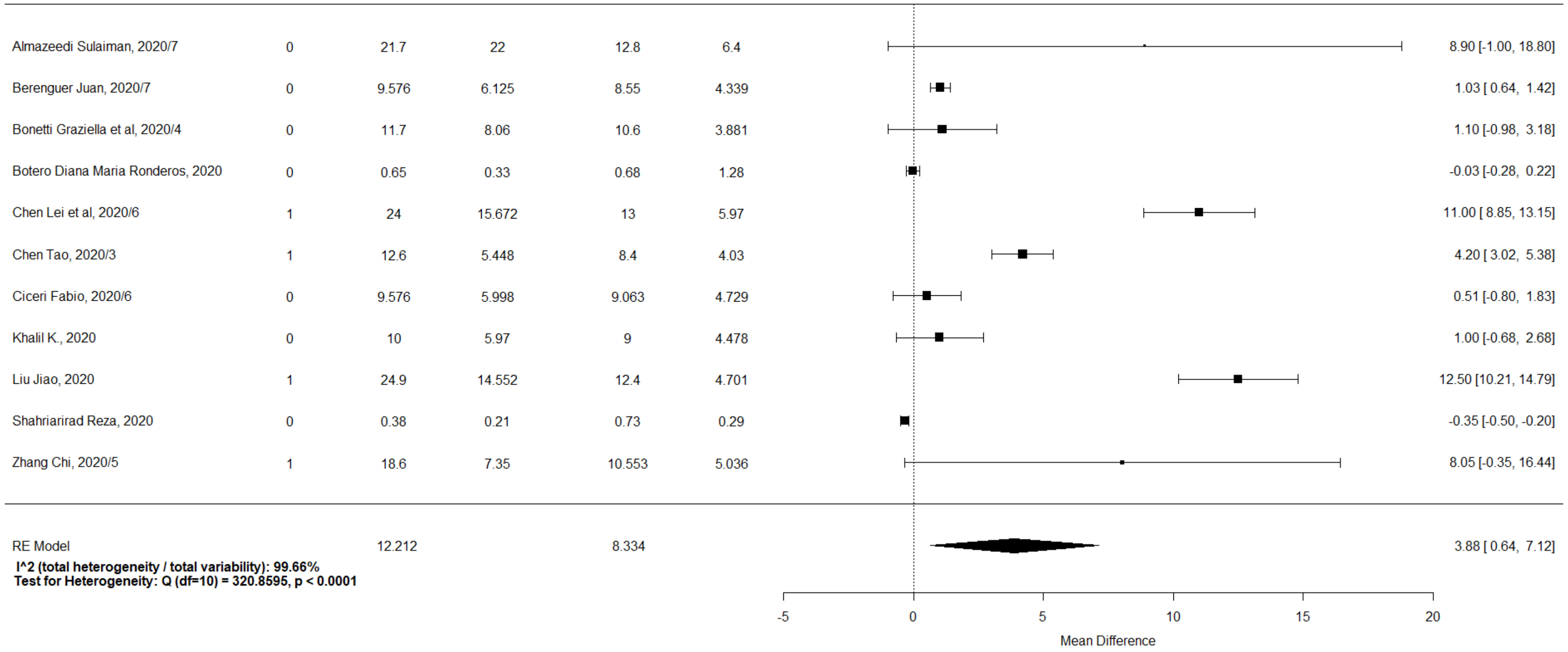

Figure S32. Forest plot of mean/median differences in lactate dehydrogenase (LDH) (U/L) between COVID-19 patients with dead or alive outcome

Random-Effects Meta-Analysis of Lactate Dehydrogenase with the Knapp and Hartung Adjustment

		Dead		Alive		
**Author(s) and Year**	**China**	**Mean or Median**	**SD or IQR/1.34**	**Mean or Median**	**SD or IQR/1.34**	**Mean/Median Difference [95% C.I.]**
Bahl Amit, 2020/9	0	511	271.642	396	169.403	115.00 [83.95, 146.05]
Berenguer Juan, 2020/7	0	318	158.955	281	124.627	37.00 [26.69, 47.31]
Bonetti Graziella et al, 2020/4	0	521	164.179	316	112.127	205.00 [158.83, 251.17]
Botero Diana Maria Ronderos, 2020	0	730	380	456	214	274.00 [120.63, 427.37]
Chen Lei et al, 2020/6	1	412	190.299	201	71.642	211.00 [184.91, 237.09]
Chen Tao, 2020/3	1	564.5	212.537	268	76.269	296.50 [255.58, 337.42]
Ciceri Fabio, 2020/6	0	521	221.642	373.714	116.88	147.29 [100.88, 193.69]
Cipriani Alberto, 2020/9	0	630	458.209	366	166.418	264.00 [60.23, 467.77]
Gayam Vijay, 2020/7	0	582	256.716	398	173.134	184.00 [135.68, 232.32]
Giacomelli Andrea, 2020/5	0	438	251.493	305	117.91	133.00 [59.85, 206.15]
Khalil K., 2020	0	499.5	148.284	465	263.06	34.50 [-21.15, 90.15]
Lee Ji Yeon et al, 2020/6	0	599	139.552	507	170.149	92.00 [20.12, 163.88]
Shahriarirad Reza, 2020	0	1116.5	1059.95	628.86	431.66	487.64 [-209.80, 1185.08]
Wang Ruoran, 2020/8	1	544	258.209	229	76.119	315.00 [257.18, 372.82]
RE Model		518.726		346.531		172.19 [116.60, 227.79]

I^2 (total heterogeneity / total variability): 95.28%
Test for Heterogeneity: Q (df=13) = 401.4016, p < 0.0001

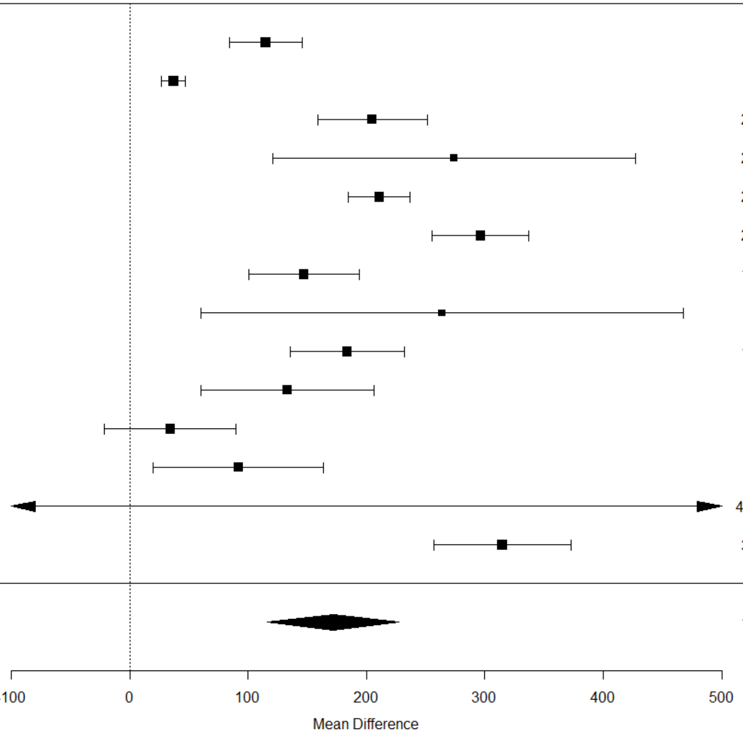

Figure S33. Forest plot of mean/median differences in D-dimer (mg/L or µg/mL) between COVID-19 patients with dead or alive outcome

Random-Effects Meta-Analysis of D-dimer with the Knapp and Hartung Adjustment

		Dead		Alive		
**Author(s) and Year**	**China**	**Mean or Median**	**SD or IQR/1.34**	**Mean or Median**	**SD or IQR/1.34**	**Mean/Median Difference [95% C.I.]**
Bahl Amit, 2020/9	0	1.442	3.088	0.885	0.838	0.56 [0.22, 0.89]
Berenguer Juan, 2020/7	0	0.74	0.881	0.548	0.452	0.19 [0.14, 0.25]
Bonetti Graziella et al, 2020/4	0	1.99	1.761	0.91	1.067	1.08 [0.60, 1.56]
Botero Diana Maria Ronderos, 2020	0	8.705	17.608	1.682	4.759	7.02 [0.07, 13.97]
Brill Simon E., 2020	0	1.577	1.294	1.186	1.347	0.39 [0.13, 0.65]
Chen Lei et al, 2020/6	1	2.5	5.448	0.4	0.448	2.10 [1.36, 2.84]
Chen Tao, 2020/3	1	4.6	14.701	0.6	0.746	4.00 [1.29, 6.71]
Ciceri Fabio, 2020/6	0	3.15	12.649	1.223	1.706	1.93 [-0.62, 4.48]
Cipriani Alberto, 2020/9	0	2.846	2.192	0.58	1.353	2.27 [1.27, 3.27]
Gayam Vijay, 2020/7	0	3.847	6.026	1.833	1.521	2.01 [0.97, 3.06]
Giacomelli Andrea, 2020/5	0	1.74	2.917	0.808	0.736	0.93 [0.10, 1.76]
Khalil K., 2020	0	2.062	1.421	1.019	0.885	1.04 [0.65, 1.43]
Liu Jiao, 2020	1	17.8	38.806	0.8	0.896	17.00 [10.93, 23.07]
Sun Haiying, 2020	1	4.16	14.56	0.79	0.866	3.37 [0.77, 5.97]
Vena Antonio, 2020/7	0	1.317	1.006	0.867	0.634	0.45 [0.24, 0.66]
Wang Ruoran, 2020/8	1	4.98	12.396	0.63	1.075	4.35 [1.60, 7.10]
Ye Wenjing, 2020	1	1.81	6.582	0.35	0.351	1.46 [-0.33, 3.25]
Zhang Jin-jin, 2020/7	1	0.9	2.261	0.182	0.17	0.72 [0.08, 1.35]
RE Model		2.251		0.814		1.44 [0.56, 2.31]

I^2 (total heterogeneity / total variability): 96.03%
Test for Heterogeneity: Q (df=17) = 151.6949, p < 0.0001

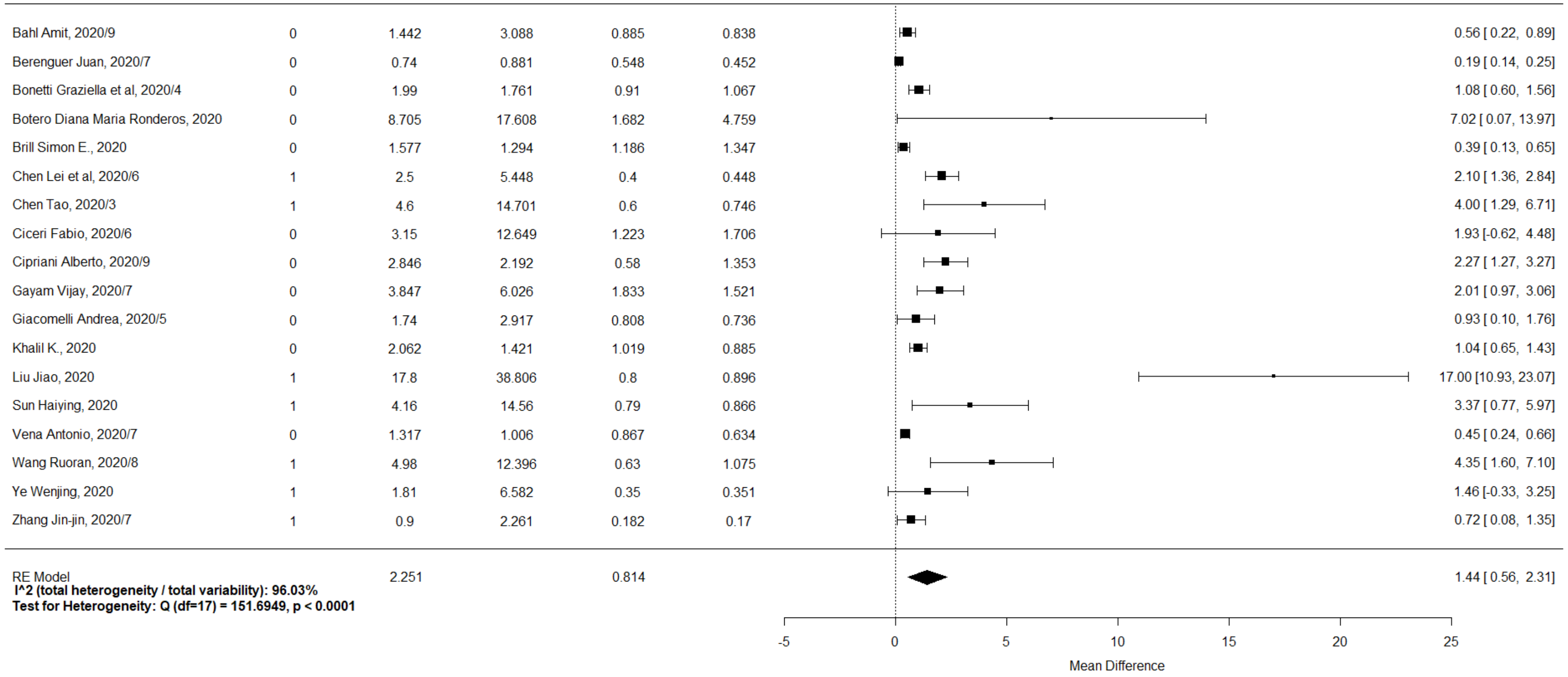

Figure S34. Forest plot of mean/median differences in C-reactive protein (CRP) (mg/L) between COVID-19 patients with dead or alive outcome

Random-Effects Meta-Analysis of C-Reactive Protein (CRP) with the Knapp and Hartung Adjustment

		Dead		Alive		
Author(s) and Year	China	Mean or Median	SD or IQR/1.34	Mean or Median	SD or IQR/1.34	Mean/Median Difference [95% C.I.]
Almazeedi Sulaiman, 2020/7	0	162	151	18	34	144.00 [76.07, 211.93]
Bahl Amit, 2020/9	0	155.9	96.493	94.8	75.597	61.10 [49.75, 72.45]
Berenguer Juan, 2020/7	0	87	97.015	44	58.955	43.00 [36.95, 49.05]
Bonetti Graziella et al, 2020/4	0	165.65	118.619	60.3	97.612	105.35 [69.76, 140.94]
Brill Simon E., 2020	0	131	93.284	68	81.343	63.00 [45.67, 80.33]
Chen Lei et al, 2020/6	1	10	52.239	3	6.716	7.00 [-0.11, 14.11]
Chen Tao, 2020/3	1	113	74.104	26.2	35.149	86.80 [72.10, 101.50]
Ciceri Fabio, 2020/6	0	126	119.403	82.79	69.282	43.21 [18.01, 68.41]
Cipriani Alberto, 2020/9	0	185	105.224	120	71.642	65.00 [16.54, 113.46]
Gayam Vijay, 2020/7	0	148	102.239	88	91.791	60.00 [39.47, 80.53]
Giacomelli Andrea, 2020/5	0	130	122.015	40.9	62.761	89.10 [53.42, 124.78]
Javanlian Mostafa, 2020	0	92	172.388	47.15	128.433	44.85 [-37.56, 127.26]
Khalil K., 2020	0	120	79.776	84.7	67.91	35.30 [12.26, 58.34]
Lee Ji Yeon et al, 2020/6	0	12.8	6.716	2.6	4.851	10.20 [7.07, 13.33]
Liu Jiao, 2020	1	160	39.478	22.5	46.94	137.50 [130.69, 144.31]
Shahriarirad Reza, 2020	0	35	25.27	34.28	20.23	0.72 [-16.24, 17.68]
Vena Antonio, 2020/7	0	109	71.567	57.2	68.284	51.80 [35.08, 68.52]
Wang Ruoran, 2020/8	1	92.2	84.925	11.2	32.015	81.00 [61.87, 100.13]
Ye Wenjing, 2020	1	65.45	54.545	12.31	34.507	53.14 [37.80, 68.48]
Zerah Lorene, 2020/8	0	189	123.881	86	85.075	103.00 [86.13, 119.87]
Zhang Chi, 2020/5	1	87.3	73.4	29.624	35.201	57.68 [-25.75, 141.11]
Zhang Jin-jin, 2020/7	1	105.2	79.851	22.53	30.071	82.67 [59.99, 105.35]
RE Model		110.82		47.714		63.11 [46.58, 79.63]

I^2 (total heterogeneity / total variability): 97.27%
Test for Heterogeneity: Q (df=21) = 1418.3785, p < 0.0001

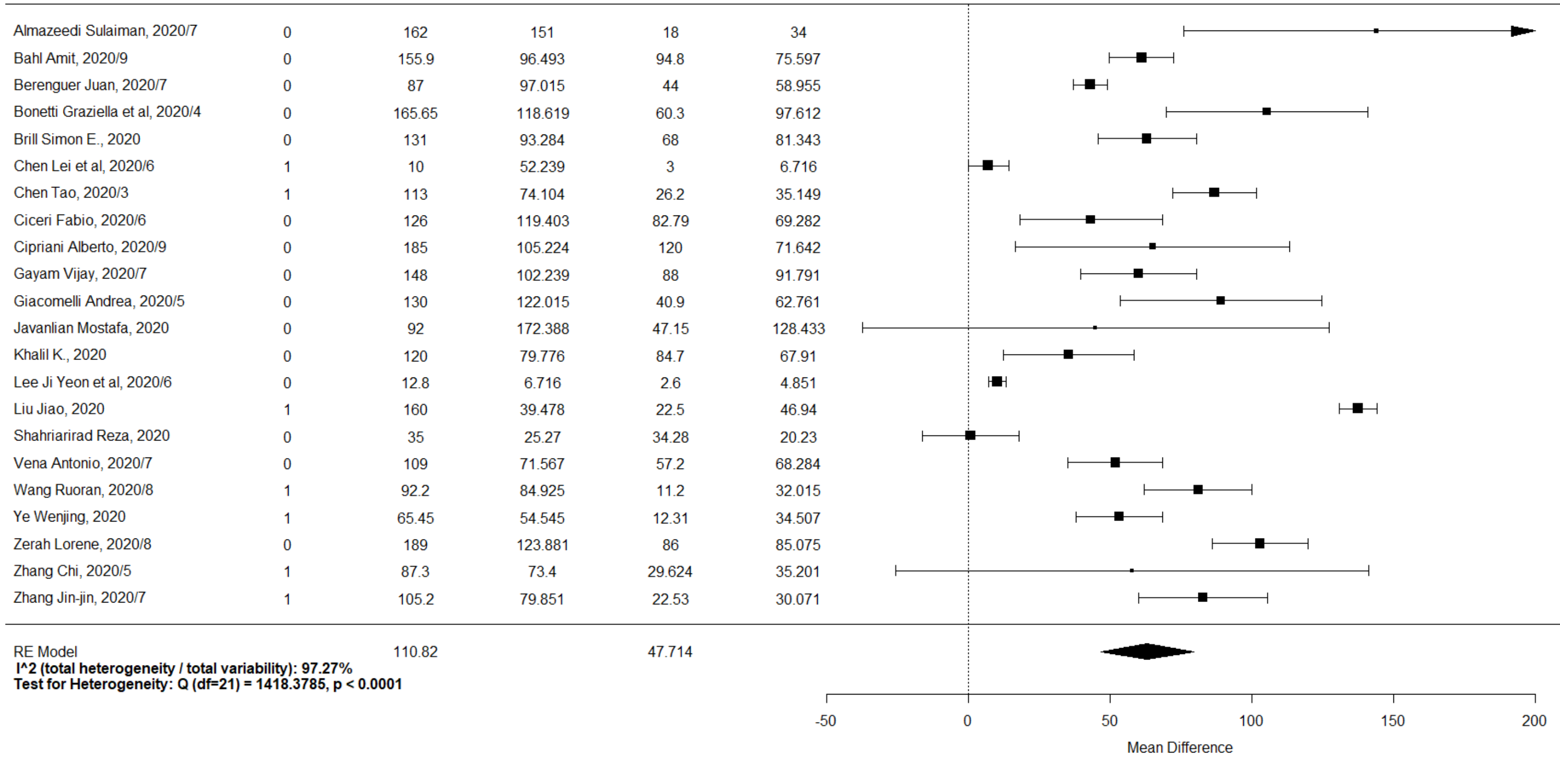

Figure S35. Forest plot of mean/median differences in procalcitonin (PCT) (×100 ng/mL) between COVID-19 patients with dead or alive outcome

Random-Effects Meta-Analysis of Procalcitonin (PCT) with the Knapp and Hartung Adjustment

Author(s) and Year	China	Dead: Mean or Median	Dead: SD or IQR/1.34	Alive: Mean or Median	Alive: SD or IQR/1.34	Mean/Median Difference [95% C.I.]
Bahl Amit, 2020/9	0	0.47	0.91	0.12	0.179	0.35 [0.25, 0.45]
Berenguer Juan, 2020/7	0	0.22	0.343	0.09	0.082	0.13 [0.11, 0.15]
Botero Diana Maria Ronderos, 2020	0	3.3	9.2	0.39	0.45	2.91 [-0.70, 6.52]
Brill Simon E., 2020	0	0.37	0.881	0.2	0.246	0.17 [0.04, 0.30]
Chen Lei et al, 2020/6	1	0.3	0.373	0.05	0.045	0.25 [0.20, 0.30]
Chen Tao, 2020/3	1	0.33	0.381	0.05	0.037	0.28 [0.21, 0.35]
Ciceri Fabio, 2020/6	0	1.18	2.649	0.475	0.446	0.71 [0.17, 1.24]
Gayam Vijay, 2020/7	0	1.57	2.187	0.58	0.858	0.99 [0.60, 1.38]
Sun Haiying, 2020	1	0.36	0.649	0.05	0.045	0.31 [0.19, 0.43]
Wang Ruoran, 2020/8	1	0	0.002	0.002	0.003	-0.00 [-0.00, -0.00]
Ye Wenjing, 2020	1	0.12	0.261	0.04	0	0.08 [0.01, 0.15]
Zhang Chi, 2020/5	1	0.27	0.18	0.12	0.052	0.15 [-0.05, 0.35]
Zhang Jin-jin, 2020/7	1	0.26	0.373	0.05	0.074	0.21 [0.11, 0.32]
RE Model		0.341		0.104		0.24 [0.10, 0.37]

I^2 (total heterogeneity / total variability): 98.20%
Test for Heterogeneity: Q (df=12) = 459.7979, p < 0.0001

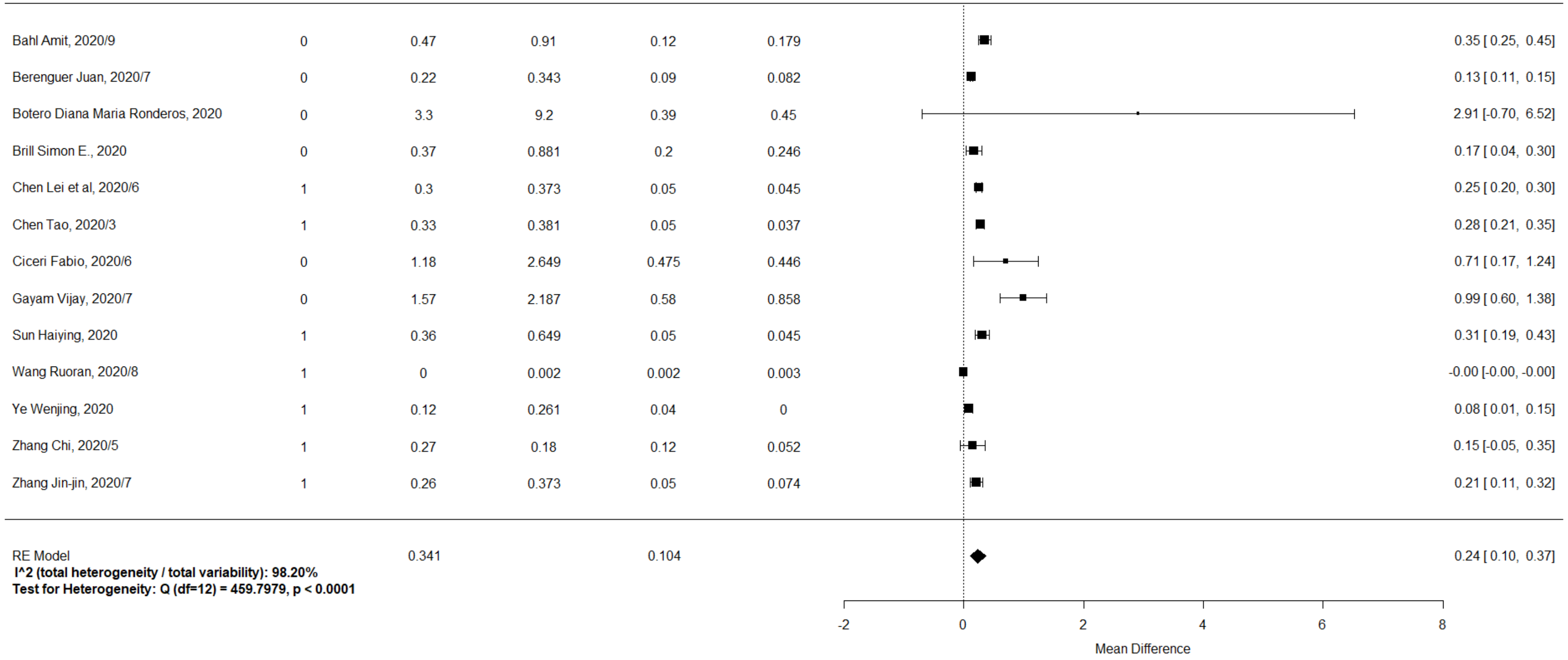

Figure S36. Forest plot of mean/median differences in hypersensitive troponin I (hs-cTnI) (×100 ng/mL) between COVID-19 patients with dead or alive outcome

Random-Effects Meta-Analysis of Hypersensitive Troponin I (hs-cTnI) with the Knapp and Hartung Adjustment

Author(s) and Year	China	Dead		Alive		Mean/Median Difference [95% C.I.]
		Mean or Median	SD or IQR/1.34	Mean or Median	SD or IQR/1.34	
Bahl Amit, 2020/9	0	0.04	0.075	0.01	0.015	0.03 [0.02, 0.04]
Bonetti Graziella et al, 2020/4	0	0.048	0.215	0.008	0.007	0.04 [-0.01, 0.09]
Botero Diana Maria Ronderos, 2020	0	0.165	0.075	0.133	0.109	0.03 [-0.00, 0.07]
Brill Simon E., 2020	0	0.042	0.038	0.012	0.02	0.03 [0.02, 0.04]
Chen Lei et al, 2020/6	1	0.161	0.497	0.003	0.005	0.16 [0.09, 0.23]
Chen Tao, 2020/3	1	0.041	0.107	0.003	0.004	0.04 [0.02, 0.06]
Ciceri Fabio, 2020/6	0	0.025	0.036	0.01	0.011	0.02 [0.01, 0.02]
Cipriani Alberto, 2020/9	0	0.279	0.773	0.016	0.023	0.26 [-0.08, 0.60]
Gayam Vijay, 2020/7	0	0.065	0.093	0.03	0.015	0.04 [0.02, 0.05]
Khalil K., 2020	0	0.034	0.057	0.01	0.016	0.02 [0.01, 0.04]
Sun Haiying, 2020	1	0.04	0.169	0.006	0.007	0.03 [0.00, 0.06]
Zhang Chi, 2020/5	1	0.18	0.2	0.021	0.042	0.16 [-0.07, 0.39]
RE Model		0.045		0.016		0.03 [0.02, 0.04]

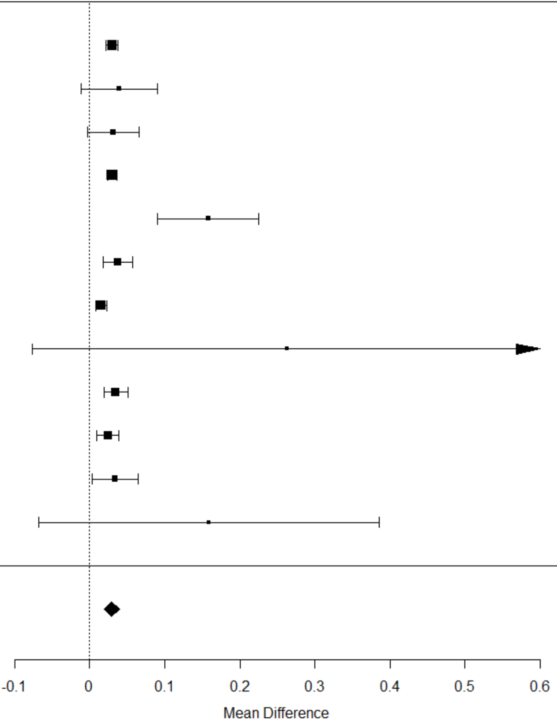

I^2 (total heterogeneity / total variability): 48.90%
Test for Heterogeneity: Q (df=11) = 31.2498, p = 0.0010
